# Microhydration Dynamics in Molecular Photoswitches: Equilibrium State Reconfiguration in Imine‐Based Architectures

**DOI:** 10.1002/anie.202506531

**Published:** 2025-07-07

**Authors:** Nuno M. Campos, Rita J. C. Roque, Pablo Pinacho, Corina H. Pollok, Christian Merten, Pedro S. P. Silva, Manuela R. Silva, Melanie Schnell, Sérgio R. Domingos

**Affiliations:** ^1^ CFisUC, Department of Physics University of Coimbra, 3004‐516 Coimbra Portugal; ^2^ Deutsches Elektronen‐Synchrotron DESY Notkestr. 85, 22607 Hamburg Germany; ^3^ Department of Physical Chemistry and Inorganic Chemistry IU‐CINQUIMA University of Valladolid Paseo Belen 7, 47011 Valladolid Spain; ^4^ Ruhr‐Universität Bochum Fakultät für Chemie und Biochemie Organische Chemie II Universitätsstraße 150, 44801 Bochum Germany; ^5^ Institut für Physikalische Chemie Christian‐Albrechts‐Universität zu Kiel Max‐Eyth‐Str. 1, 24118 Kiel Germany

**Keywords:** Artificial molecular motors, Microsolvation, Noncovalent interactions, Quantum chemistry calculations, Rotational spectroscopy

## Abstract

The functional performance of a molecular photoswitch relies strongly on its ability to undergo structural changes in solution. In this context, microsolvation studies in the gas phase provide access to the conformational panorama of these systems in a size‐controlled hydrated environment. Here, we exploit this gas‐phase vantage point alongside quantum‐chemistry calculations to study the structural properties and microhydration dynamics of camphorquinone imine, a chiral molecule holding the functionality to engage in a motor‐like function upon light activation. Using molecular rotational resonance spectroscopy with supersonic jets, we detect and analyze the first‐ and second‐order water complexes of the chiral imine. Our findings reveal that initial hydration steps significantly impact the equilibrium between open (E) and closed (Z) forms, culminating in a reversal of relative stability for the switch states. Despite being captured at rotational temperatures near 1 K, we find that water molecules exhibit notable mobility due to the lack of prominent stabilizing secondary interactions. Additionally, the assignment of a key higher‐energy closed (Z) water complex provides insights into the energy required for switching between (E) and (Z) states during collisional cooling. We discuss these effects and rationalize them in terms of molecular forces and internal dynamics governing early solvation.

## Graphical abstract

The performance of molecular photoswitches relies on their structural adaptability in solution. Employing gas‐phase rotational spectroscopy and quantum chemistry, we explore the microhydration of camphorquinone imine and show that water binding shifts the E/Z equilibrium, even reversing relative stabilities, while enabling dynamic motion at low temperatures. These insights reveal the interplay of structure, solvation, and switching energetics.

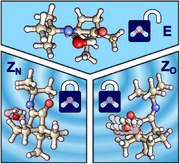

## Introduction

The clever and directed design of molecular architectures that are able to alter their functionality when driven by external stimuli is a quest of modern synthetic chemistry.^[^ ^1^ ^]^ A great focus has been put into the development of novel molecular motors and switches that are able to perform motion at the molecular scale in a controlled manner using light as an energy source.^[^ ^2^ ^]^ A particular class of these molecular motors/switches are those including C═C,^[^ ^3^, ^4^, ^5^ ^]^ C═N,^[^ ^6^, ^7^, ^8^, ^9^ ^]^ and N═N^[^ ^10^, ^11^, ^12^, ^13^, ^14^ ^]^ chemical groups. Compounds with C═C functionality, such as Feringa's motors based on overcrowded alkenes, have evolved far beyond mere scientific curiosities in the last two decades, and are now at the core of major breakthroughs in molecular nanotechnology. Such molecular architectures are able to convert energy into controlled motion to drive a system out of equilibrium. As examples, such systems have been used to control biological molecules,^[^ ^15^, ^16^ ^]^ have been incorporated into metal organic frameworks,^[^ ^17^, ^18^ ^]^ have been used to control the assembly and disassembly of supramolecular complexes^[^ ^19^, ^20^ ^]^ and even used to create a four‐wheeled nanocar.^[^ ^21^ ^]^

The class of motors including the C═N group is an intriguing addition to the molecular machinery toolbox because of their potential to perform both a four‐step or two‐step unidirectional rotation.^[^ ^7^ ^]^ Although their properties remain largely unexplored, they are arguably the simplest examples of synthetic molecular motors. These motors can undergo a photochemical out‐of‐plane C═N bond rotation, confirmed only theoretically, and a thermally driven in‐plane N inversion, demonstrated both experimentally and theoretically.^[^ ^6^ ^]^ The assumption is that in the presence of chirality, the photochemical C═N bond rotation should exhibit a preferred directionality due to displacement through chiral subspaces in the molecule, which guarantee asymmetrical excited‐state surfaces. The two‐step mode of action, in which these processes occur one after the other, should thus impart a motor‐like motion to the system. Due to the orthogonality of the photochemical and thermal pathways, and assuming that imines with chirality at a suitable distance exhibit preferred directionality in the double bond rotation, any chiral imine can be considered, in principle, a molecular motor.^[^ ^6^ ^]^

Regardless of their inherent constitution, the functionality of any molecular motor should be assessed when in the presence of a solvating agent since the interactions of the solvent with the motor and its influence on the interactions with other systems may alter the functionality of the motor.^[^ ^22^, ^23^, ^24^, ^25^ ^]^ Molecular architectures like these are commonly investigated using an arsenal of spectroscopic techniques, typically in condensed‐phase scenarios.^[^ ^26^, ^27^ ^]^ However, in the first stages of conceptual design of functional molecular machines, an investigation of the structural dynamics of local sites is highly pertinent, especially if one is able to do so using a “magnifying glass.” Microsolvation studies in the gas phase can do just that. The stepwise growth of an interacting layer around a target system provides the canvas to study the first steps of solvation and its effect on the structure and conformational landscape.^[^ ^28^, ^29^, ^30^, ^31^, ^32^, ^33^, ^34^, ^35^, ^36^ ^]^ This information can be invaluable to tweak molecular design concepts and revise synthesis routes.

**Figure 1:**
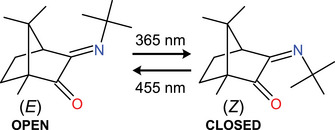
Chemical structures and interconversion wavelengths for the two isomers (E) and (Z) of the camphorquinone imine switch (**1**).

In this paper, we report an in‐depth investigation of the microsolvation induced structural properties of a camphorquinone derived imine‐based molecular switch, whose configurations are presented in Figure 1. Previous studies^[^ ^6^ ^]^ found that in an acetonitrile solution the E isomer is favored over the Z isomer, in the following ratio E:Z → 99:1. This changes to a ratio of E:Z → 21:79 in the photostationary state when the switch is exposed to light with a wavelength of 365 nm. The Z→E back‐isomerization in this solution was shown to have a half‐life of 10 min, due to an energy barrier $\Delta G_{298K}^{*}$ of about 92 kJ ${\mathrm{mol}}^{-1}$, but can also be induced by exposure to visible light with a wavelength of 455 nm. In cryogenically cooled matrices, it has also been shown that the E isomer is favored over the Z isomer.^[^ ^37^ ^]^

Interestingly, the authors found a discrepancy between matrix‐isolation results and vacuum predictions, where Z seems to be greatly favored over E. Here, and to shed light into the apparent environment‐dependent conformational preferences and underlying structural dynamics at play, we employ molecular rotational resonance (MRR) spectroscopy in combination with quantum chemistry calculations.^[^ ^38^, ^39^, ^40^, ^41^, ^42^ ^]^ Broadband high‐resolution rotational data is used to construct and validate structural models obtained from quantum‐chemistry, which elucidate the effects of dynamic microhydration on the energetic balance between open and closed configurations. Based on those models we achieve a comprehensive picture of the intermolecular forces at work in the mediation of the energetic balance between switch states.

## Results and Discussion

**Figure 2:**
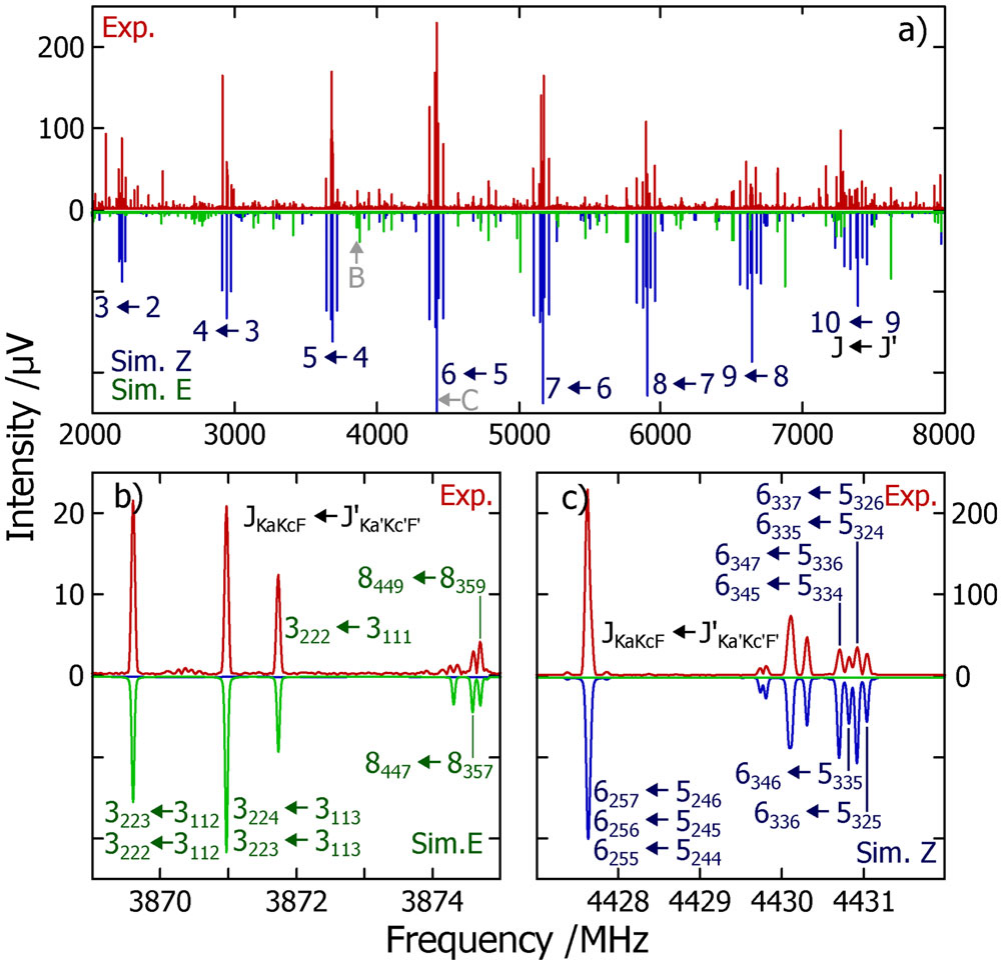
a) Full experimental spectrum of camphorquinone imine (upper trace, in red) and fitted (lower trace, in blue and green) rotational transitions for the Z (blue) and E (green) isomers. b) Portion of the experimental spectrum and fitted rotational transitions for the E isomer. c) Portion of the experimental spectrum and fitted rotational transitions for the Z isomer. In panel (a), only the J quantum number is used to identify groups of transitions for the Z isomer.

**Figure 3:**
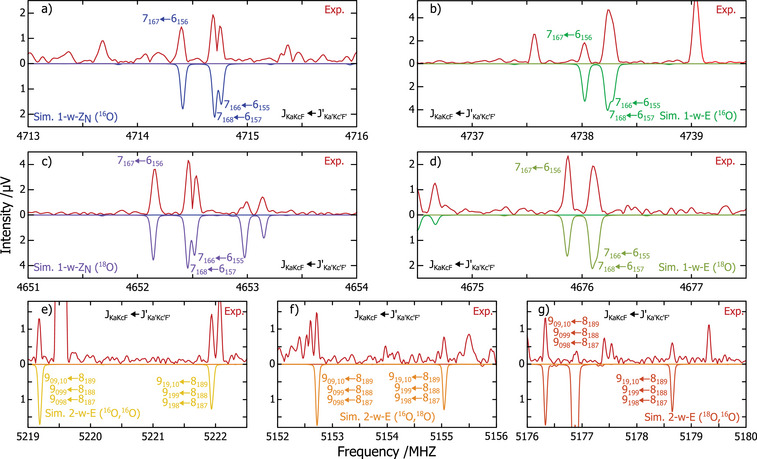
a), c) Portions of the broadband spectrum showing rotational transitions of two isotopologues of 1‐w‐$Z_{N}$. b), d) Portions of the broadband spectrum showing rotational transitions of two isotopologues of 1‐w‐E. e), f), and g) Portions of the broadband spectrum showing rotational transitions of three isotopologues of 2‐w‐E. The extra transitions in panel (e) are from the isolated Z isomer.

A detailed account of the experimental methods used in this work is given in the Supporting Information. In short, broadband rotational spectra were collected using the COMPACT chirped‐pulse Fourier transform microwave spectrometer operating in Hamburg.^[^ ^43^ ^]^ This type of instrument has been described in detail by Brown et al.,^[^ ^44^ ^]^ and it will be referred to hereon as MRR spectrometer. In Figures 2, 3, and 5 (upper traces, in red), we show spectral portions of experimental rotational spectra. The lower traces are simulated spectra obtained from recurrent fits using Watson's A‐reduced semirigid rotor Hamiltonian^[^ ^45^ ^]^ in the $I^{r}$ representation as implemented in PGOPHER.^[^ ^46^ ^]^ The rotational transitions $J_{K_{a}K_{c}F}\leftarrow J_{K_{a}^{\prime }K_{c}^{\prime }F^{\prime }}^{\prime }$ are denoted by the rotational quantum numbers J, $K_{a}$, $K_{c}$ and F with J being the rotational angular momentum quantum number, $K_{a}$ and $K_{c}$ being the projections of J onto the principal axes at the prolate and oblate symmetric top limits, respectively, and F being the total angular momentum quantum number (including the nuclear spin, I(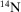) = 1).

**Figure 4:**
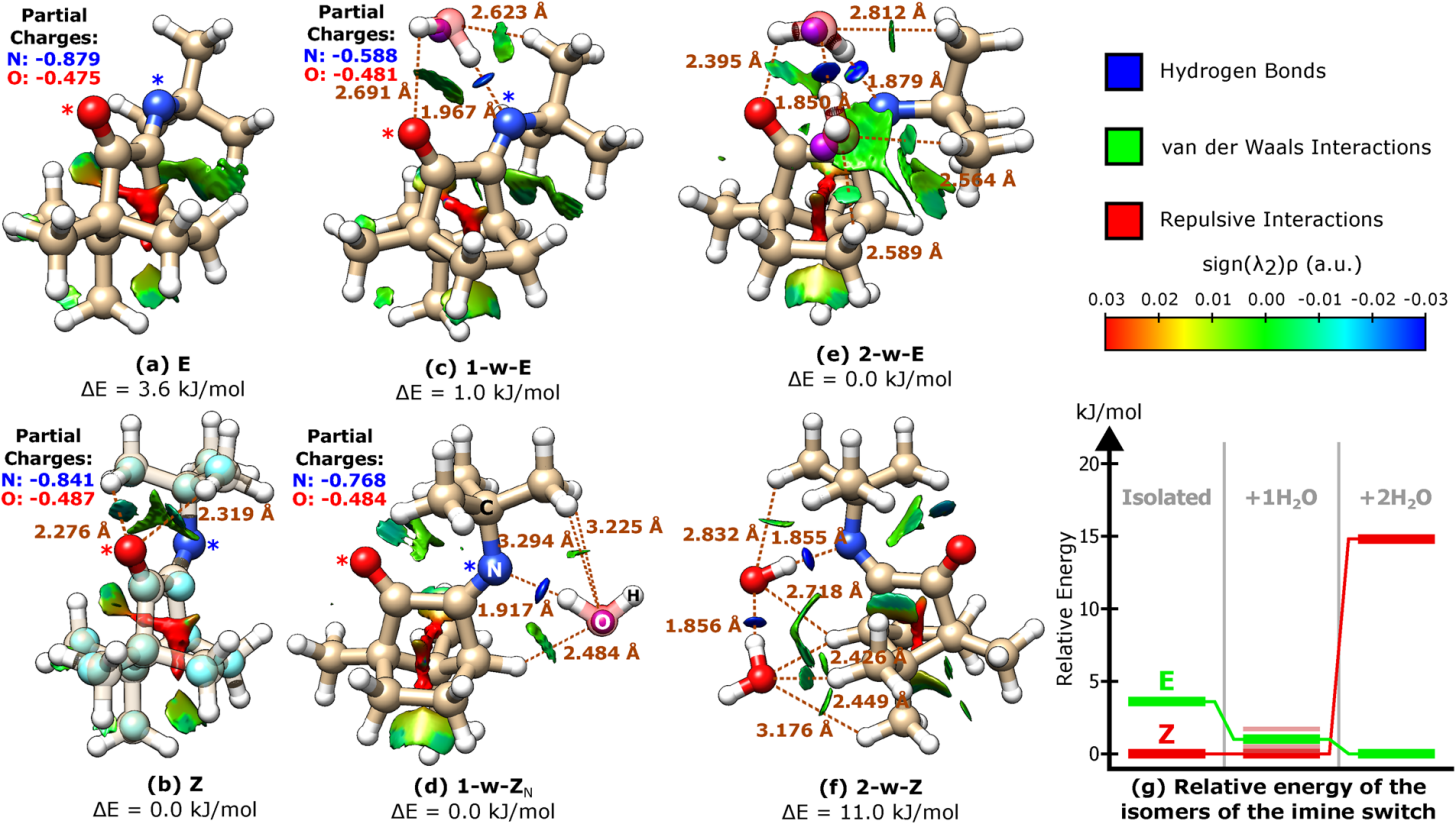
a)–f) Molecular structures of all isomers of **1** and their corresponding first‐ and second‐order microsolvated species calculated at the B3LYP‐D3(BJ)/def2‐TZVP level of theory. Relevant intermolecular and intramolecular interactions (distances in Å) are depicted and the NCI surfaces plotted. Relative zero‐point corrected energies for each configuration are given (in kJ ${\mathrm{mol}}^{-1}$) as reported in Tables 1 and 2. In (b) the light blue spheres represent the experimentally determined ($r_{s}$ structure) carbon atom positions, while in (c)–(e) the pink spheres represent the experimentally determined oxygen atom positions. In (a)–(d) the partial charges of the N and O atoms, marked by asterisks, are also presented. (g) Relative energy of the isomers of the imine switch. The energies represented are up to scale. For the monohydrated Z isomer, the energies of the multiple minima found in the flat region of the relaxed energy scan (Figure 6) are presented by the faded red traces.

**Figure 5:**
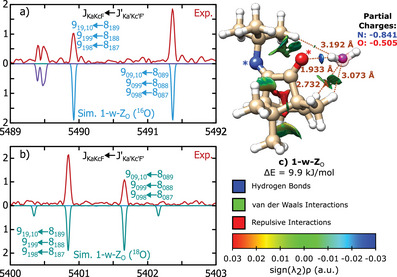
a), b) Portions of the broadband spectrum showing rotational transitions of the two isotopologues of 1‐w‐$Z_{O}$. c) Molecular structure of 1‐w‐$Z_{O}$ calculated at the B3LYP‐D3(BJ)/def2‐TZVP level of theory. Relevant intermolecular and intramolecular interactions (distances in Å) are depicted and the NCI surfaces plotted along with partial charges for the O and N atoms of the imine. The pink sphere represents the experimentally determined oxygen atom position.

The spectroscopic parameters extracted from the experimental data are given in Tables 1, 2, 3 and include primary rotational constants (A,B,C), quartic centrifugal distortion constants ($\Delta_{J},\Delta_{JK},\Delta_{K},\delta_{J},\delta_{K}$), as well as nuclear quadrupole coupling constants ($\chi_{aa},\chi_{bb}-\chi_{cc}$) arising due to the nitrogen atom (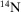) of the imine moiety. The non‐diagonal elements of the quadrupole coupling tensor were not determined or used in the fitting procedure.

**Table 1:** Experimental and calculated (B3LYP‐D3(BJ)/def2‐TZVP) spectroscopic parameters for the isomers of **1**; types of transitions observed (a‐type, b‐type, c‐type) and predicted electric dipole moment components (in Debye); number of lines (N) included in the fit and its standard deviation (σ, in kHz). Calculated relative zero‐point corrected energies with implicit solvation using the PCM model (ε=80.4) and without are given in kJ ${\mathrm{mol}}^{-1}$.

	E		Z	
Parameters	Theory	Experiment	Theory	Experiment
A (MHz)	924.27	929.138073(90)	976.45	980.63596(14)
B (MHz)	383.97	385.841608(37)	374.61	377.197046(43)
C (MHz)	359.29	361.085018(41)	358.68	360.984655(41)
$\Delta_{J}$ (kHz)	–	0.00389(18)	–	0.00585(25)
$\Delta_{JK}$ (kHz)	–	–	–	0.02106(80)
$\Delta_{K}$ (kHz)	–	−0.0195(21)	–	−0.1685(56)
$\delta_{J}$ (kHz)	–	–	–	–
$\delta_{K}$ (kHz)	–	–	–	–
$\chi_{aa}$ (MHz)	1.25	1.0837(30)	1.29	1.1056(22)
$\chi_{bb}-\chi_{cc}$ (MHz)	−8.15	−7.4471(35)	−9.01	−8.0844(38)
$|\mu_{a}|$ (D)	0.7	no	2.3	yes
$|\mu_{b}|$ (D)	4.6	yes	0.9	yes
$|\mu_{c}|$ (D)	0.7	no	0.2	no
N	–	387	–	594
σ /(kHz)	–	4.55	–	4.55
$\Delta E_{ZPC}$ (kJ ${\mathrm{mol}}^{-1}$)	3.6	–	0.0	–
ΔE (PCM) (kJ ${\mathrm{mol}}^{-1}$)	0.0	–	13.8	–

**Table 2:** Experimental and calculated (B3LYP‐D3(BJ)/def2‐TZVP) spectroscopic parameters for the first‐ and second‐order microsolvated species of **1**; types of transitions observed (a‐type, b‐type, c‐type) and predicted electric dipole moment components (in Debye); number of lines (N) included in the fit and its standard deviation (σ, in kHz). Calculated relative zero‐point corrected energies with implicit solvation using the PCM model (ε=80.4) and without are given in kJ ${\mathrm{mol}}^{-1}$.

	1‐w‐E		1‐w‐$Z_{N}$		2‐w‐E		2‐w‐$Z_{N}$
Parameters	Theory	Experiment	Theory	Experiment	Theory	Experiment	Theory
A (MHz)	687.37	681.56455(21)	685.61	677.40586(22)	559.77	550.88858(20)	495.42
B (MHz)	358.02	359.42981(14)	362.66	363.18760(19)	331.42	334.45608(13)	353.53
C (MHz)	305.95	305.92344(12)	301.69	300.59866(13)	282.93	283.69184(15)	259.67
$\Delta_{J}$ (kHz)	–	0.03955(50)	–	0.00937(62)	–	0.00703(48)	–
$\Delta_{JK}$ (kHz)	–	0.2530(16)	–	–	–	0.01580(49)	–
$\Delta_{K}$ (kHz)	–	–	–	–	–	–	–
$\delta_{J}$ (kHz)	–	−0.00812(34)	–	0.00305(37)	–	0.002279(62)	–
$\delta_{K}$ (kHz)	–	–	–	−0.0664(31)	–	–	–
$\chi_{aa}$ (MHz)	0.58	0.507(11)	0.62	0.670(12)	0.44	0.719(26)	0.66
$\chi_{bb}-\chi_{cc}$ (MHz)	−7.94	−6.121(15)	−7.47	−7.013(24)	−0.19	−0.659(50)	−5.30
$|\mu_{a}|$ (D)	3.4	yes	1.1	yes	2.5	yes	0.6
$|\mu_{b}|$ (D)	5.1	yes	0.6	no	3.1	yes	0.5
$|\mu_{c}|$ (D)	0.0	no	0.2	no	1.6	yes	2.1
(N)	–	355	–	168	–	166	–
σ (kHz)	–	8.61	–	6.43	–	10.53	–
$\Delta E_{ZPC}$ (kJ ${\mathrm{mol}}^{-1}$)	1.1	–	0.0	–	0.0	–	11.0
ΔE (PCM) (kJ ${\mathrm{mol}}^{-1}$)	0.0	–	6.2	–	0.0	–	14.8

**Table 3:** Experimental and calculated (B3LYP‐D3(BJ)/def2‐TZVP) spectroscopic parameters for 1‐w‐$Z_{O}$; types of transitions observed (a‐type, b‐type, c‐type) and predicted electric dipole moment components (in Debye); number of lines (N) included in the fit and its standard deviation (σ, in kHz). Calculated relative zero‐point corrected energies (relative to 1‐w‐$Z_{N}$) with implicit solvation using the PCM model (ε=80.4) and without are given in kJ ${\mathrm{mol}}^{-1}$.

	1‐w‐$Z_{O}$	
Parameters	Theory	Experiment
A (MHz)	629.54	596.1688(18)
B (MHz)	370.96	369.14709(29)
C (MHz)	301.99	296.84249(19)
$\Delta_{J}$ (kHz)	–	0.01672(95)
$\Delta_{JK}$ (kHz)	–	0.5716(60)
$\Delta_{K}$ (kHz)	–	–
$\delta_{J}$ (kHz)	–	−0.00680(55)
$\delta_{K}$ (kHz)	–	0.165(10)
$\chi_{aa}$ (MHz)	1.02	1.171(25)
$\chi_{bb}-\chi_{cc}$ (MHz)	−3.49	−0.654(53)
$|\mu_{a}|$ (D)	1.8	yes
$|\mu_{b}|$ (D)	0.7	yes
$|\mu_{c}|$ (D)	0.9	no
(N)	–	184
σ (kHz)	–	8.50
$\Delta E_{ZPC}$ (kJ ${\mathrm{mol}}^{-1}$)	9.9	–
ΔE (PCM) (kJ ${\mathrm{mol}}^{-1}$)	8.3	–

### Monomeric E and Z Forms

The full broadband spectrum (2–8 GHz) is shown in panel (a) of Figure 2 and depicts rotational transitions from two molecular species that dominate the spectral window at this level of the dynamic range. Panels (b) and (c) of Figure 2 display narrow portions of this spectral range, highlighting signatures of the E (open) and Z (closed) isomers, **1**‐E and **1**‐Z, respectively (as illustrated in Figure 1). The structures of the Z and E isomers in isolation are shown in panels (a) and (b) of Figure 4.

Table 1 summarizes the fitted and calculated rotational parameters for these molecular species. For the monomeric species, the B3LYP‐D3(BJ)/def2‐TZVP^[^ ^47^, ^48^, ^49^, ^50^, ^51^, ^52^, ^53^, ^54^ ^]^ method captures the fitted primary rotational constants (A, B, C) within 1% accuracy. In panel (a) of Figure 2, a strong a‐type pattern spanning eight J levels is present (in blue), which we can confidently assign to **1**‐Z, with predicted $\mu_{a}$ = 2.3 D. A strong b‐type pattern is also present (in green), which can be confidently assigned to **1**‐E that is not surprising given the predicted $\mu_{b}=4.6D$ and the much weaker dipole moment components anticipated for a‐ and c‐type transitions. For **1**‐Z it was also possible to assign satellite transitions emerging from singly‐substituted 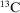 isotopologues in natural abundance and consequently derive the atomic coordinates of carbon (light‐blue spheres in Figure 4) using a Kraitchman analysis.^[^ ^55^, ^56^ ^]^

It can be observed that the a‐type pattern of **1**‐Z is significantly more intense than the b‐type pattern of **1**‐E, despite the former having a lower predicted electric dipole moment when compared to the latter. Based on these relative intensities and on the predicted dipole moments, the relative abundances of the Z and E isomers are estimated to be 88% and 12%, respectively. Considering the energy difference predicted at this level of theory after employing a zero point correction to the electronic energies – ΔE=+3.6 kJ ${\mathrm{mol}}^{-1}$ –, and assuming the relative abundances previously referenced, we can estimate that the conformational distribution froze at a temperature of about 210 K.

At this point, rotational spectroscopy validates a plausible preference for Z over E, in full agreement with the current and previous vacuum calculations, and in opposition with both solution and matrix‐isolation observations that capture the E form in superior abundance.^[^ ^6^, ^37^ ^]^ All this information suggests that a strong influence of the solvating agents on the conformational landscape is at play. Therefore, in an attempt to break down the role of the local solvent interactions in the relative stability of both switchable configurations, we performed a second set of MRR experiments where we co‐expanded **1** with $H_{2}O$ (the experimental details are given in the Supporting Information). With this approach, we created favorable experimental conditions for the observation of water aggregates in the gas phase, enabling structural studies of microhydrates.

### Low‐Energy Hydrated Species

Portions of the rotational spectrum of **1** obtained with a wet carrier gas are shown in Figure 3. Selected assignments of rotational transitions are depicted in panels (a) and (b) for the one‐water complexes, 1‐w‐$Z_{N}$ and 1‐w‐E, respectively. Panel E displays selected assignments for the two‐water complex, 2‐w‐E. Figure 4 shows the structures of both isomers of **1** in isolation (a, b), mono‐hydrated (c, d) and doubly‐hydrated (e, f), obtained using ORCA^[^ ^57^, ^58^, ^59^ ^]^ at the B3LYP‐D3(BJ)/def2‐TZVP^[^ ^47^, ^48^, ^49^, ^50^, ^51^, ^52^, ^53^, ^54^ ^]^ level of theory. Predictions for micro‐solvated species were first tracked down using CREST,^[^ ^60^, ^61^, ^62^, ^63^ ^]^ and a selection of candidate isomers, sorted by their energy, were re‐optimised at the DFT level for further analysis. The complete conformational analysis is given in the Supporting Information.

The rotational parameters for these species are well determined, with low standard deviation and theory predicting primary constants close to 1% accuracy, as can be seen in Table 2. The primary rotational constants of 1‐w‐E and 1‐w‐$Z_{N}$ are rather similar (Table 2), but the relative magnitudes of the a‐ and b‐type transitions clearly define the assignment. Although our conformational search predicted several minimum structures for 1‐w‐$Z_{N}$ (see full details in Supporting Information), only the spectroscopic constants predicted for the global minimum geometry are presented in Table 2. We will expand on this in the last section of the paper.

As an additional validation step, we performed auxiliary measurements using isotopically‐enriched $H_{2}$ 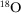 water. The assignment of new satellite rotational transitions for the singly substituted isotopologues (spectral portions in panels (c), (d), (f), and (g) in Figure 3) were used to determine new rotational parameters and solve for the coordinates of the oxygen atoms of the solvating water molecules (pink spheres in Figure 4) using the Kraitchman equations.^[^ ^55^, ^56^ ^]^ This analysis confirmed the predicted hydrated topologies for both hydrates, as well as for the larger clusters discussed later.

Interestingly, theory predicts that when a single water molecule is in play, forming either of the monohydrated species, the energy difference between the two isomers becomes marginally small when compared to that of the isolated forms, decreasing from 3.6  to 1.1 kJ ${\mathrm{mol}}^{-1}$ (Tables 1 and 2). This effect is enhanced by the presence of two water molecules to the point of driving an equilibrium‐state reversal, where the E isomer is now more stable than Z by 11.0 kJ ${\mathrm{mol}}^{-1}$ (Table 2). This gradual stability reversal with the growth of the micro‐hydration layer is clearly illustrated in the energy level scheme shown in panel (g) of Figure 4.

Furthermore, for a qualitative comparison between theory and experiment, we performed additional calculations, but instead of conventional vacuum predictions, we computed the equilibrium state energies for both E and Z isomers employing a polarizable continuum model (PCM) with ε=80.4, emulating thus the dielectric micro‐environment generated by water (see Tables 1 and 2). The results are very informative, revealing a complete reversal of relative stabilities for Z/E, in agreement with previous solution and matrix‐isolation experiments. As for the two‐water complexes, using the PCM further augments the energy gap between Z and E.

Structurally, while both 1‐w‐$Z_{N}$ and 1‐w‐E use the N atom as the primary docking site for the water molecule, the topologies of the E and Z micro‐hydrated complexes are fundamentally different. The *open* 1‐w‐E complex (Figure 4) has the tert‐butyl group detached from the carbonyl docking site, and thus the incoming water molecule uses that docking bay as expected, anchoring more strongly to the N and O atoms via hydrogen bonding, and establishing secondary interactions with the tert‐butyl hydrogen atoms, as confirmed by the auxiliary non‐covalent interaction (NCI) analysis.^[^ ^64^, ^65^ ^]^ In the 1‐w‐$Z_{N}$ complex (Figure 4), the switch is in its *closed* position, and so the tert‐butyl group engages with the carbonyl group via two CH–O interactions. Consequently, and presuming that the water molecule is not able to disrupt this intramolecular interaction, the $H_{2}O$ molecule must find an alternative binding site. Indeed, this occurs at the opposite orbital side of the N atom, with the water forming a single primary OH–N interaction. In this position, the water molecule is unable to establish secondary interactions with an electron donating group, remaining only partially locked in place. The consequences of this incomplete docking, and how it triggers the need for a dynamic description of microhydration, is discussed in detail in the last section of the paper.

The predicted structures of the dihydrated forms 2‐w‐Z (not detected) and 2‐w‐E (observed) emerge as an extension of the binding networks of 1‐w‐$Z_{N}$ and 1‐w‐E, respectively. For both, one of the water molecules is bound to a similar location as in the corresponding singly hydrated species, exhibiting similar interactions with the imine switch. The complete double‐water network is formed with a strong hydrogen bond (OH ─ O) between the two waters, as in the pure water dimer, as well as several additional secondary contacts. As can be visually inspected in panels (e) and (f) of Figure 4, the secondary interactions now confer additional grip, establishing contacts between the tert‐butyl and camphor‐moiety hydrogens with the water oxygen lone‐pairs, as well as interactions depicting purely dispersive character.

Inspired by recent findings emerging from studies on the dynamics of water molecules solvating polyethers,^[^ ^66^ ^]^ we attempted to track down trends in the interaction between the first water molecule and the docking site of E and Z isomers, taking into consideration the local charges of the N and O atoms in the docking bay. In the case of polyethers, solubilities can be extremely different depending not on steric effects, but on the local charges of the O atoms. Inductive effects were shown to have a major impact on aqueous solubilities, and consequently they may also affect photo‐stationary states of a molecular switch with a C = N functionality. To expand on this, the local charges of the O and N atoms in **1** were computed using ORCA at the B3LYP‐D3(BJ)/def2‐TZVP level of theory and found to be similar for E and Z in their isolated form. However, when we computed them for the hydrated species, we found that while changes in the local charges (isolated vs. complex) for the O atom are negligible, for the N atom that change is four times larger in 1‐w‐E when compared to 1‐w‐$Z_{N}$. These results, which are included in Figure 4, support OH–N being the primary link establishing the complex for both E and Z and inform on the looser intermolecular bond created in 1‐w‐$Z_{N}$.

A more comprehensive picture of the effect of microhydration on the energetic ordering of species is emerging at this point: discarding for the moment any uncertainties arising from local dynamics, we are presented with similar transition intensities observed for 1‐w‐E and 1‐w‐$Z_{N}$, confirming the small energy difference predicted in the calculations. This is the tipping point in the energetic reordering. Thereon, the considerable increase in energy difference between isomers predicted for the two‐water complex is confirmed by the lack of observable lines for the 2‐w‐Z species. This inversion that is predicted and also observed experimentally triggers the question: can a single water molecule induce a conformational change during the course of one or multiple collision events occurring in the supersonic jet expansion? We have seen occasionally studies where comparable occurrences take place, such as in kinetically trapped states, where a first‐order hydrate is captured, taking the monomeric species “hostage” in a high‐energy conformation without sufficient energy to undergo conformational relaxation. In the following section, we unveil an important piece of information that allows one to discuss this hypothesis from a higher vantage point.

### High‐Energy Monohydrated Species

Typically water complexes are formed from all conformers of a molecule, following their respective abundances. However, in most cases, only those formed from the most abundant conformers are formed in sufficient quantity to be observable. Literature data outlines this tendency, and few are the reported cases where this trend is challenged, a notable example being the 12‐crown‐4 ether reported by Pérez et al.^[^ ^30^ ^]^

In the present study, an intriguing spectral misfit led us to evaluate the predicted topologies for hydrates and the population of conformations in a different way. A spectral fit was obtained for an “unknown” species, meaning that the rotational parameters extracted from the fit could not be matched to any theoretical prediction from DFT. To solve this assignment problem, we implemented a computational method, developed in‐house (and thoroughly explained in the Supporting Information), where we reverse‐engineered the problem. Starting from the fitted rotational constants and the geometries of E and Z in their monomeric form, we derived likely positions in the principal axis frame where the water molecule should be placed in order to generate a set of rotational constants that would match those extracted from the spectral fit. Strikingly, our method produced results consistent with a closed Z‐type water complex, with the water molecule bound to the O atom, instead of the N atom, as seen for the previously assigned 1‐w‐Z species. Our assignment is confirmed with the identification of the corresponding singly substituted 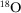 isotopologue spectrum. A portion of the spectrum, the corresponding structure and NCI analysis for 1‐w‐$Z_{O}$ are shown in Figure 5.

A close comparison of the structural and rotational parameters predicted for this new species, which we dubbed 1‐w‐$Z_{O}$, led us to conclude that an equivalent prediction was captured by DFT in our initial survey, but discarded from active spectral searches because of its relative energy of ca. 10 kJ ${\mathrm{mol}}^{-1}$ with respect to the global minimum.

An inspection of the 1‐w‐$Z_{O}$ geometry reveals a water docking occurring at the O‐site, instead of the predictably favorable N‐site, through a single hydrogen bond interaction. The lack of prevailing secondary interactions and the consequences it has on the local dynamics will be discussed in the next section. Of note is that from the point of view of the water molecule, conversion to 1‐w‐$Z_{N}$ via conformational relaxation is highly unfavorable, since we find that such a trajectory meets an energy barrier of 30 kJ ${\mathrm{mol}}^{-1}$. The water‐docking positioning in 1‐w‐$Z_{O}$ leads, in fact, to a scenario where the water becomes trapped in the course of the jet expansion, rendering it detectable in the experiment despite its high relative energy.

Consequently, this observation is very enlightening, as it strongly refutes the previous hypothesis that the water molecules could actively influence the outcome of the population distribution of open and closed conformations of the switch during the supersonic expansion. If such water‐induced conversion would take place in the jet, 1‐w‐$Z_{O}$ would relax into 1‐w‐E promptly, leaving 1‐w‐$Z_{O}$ undetectable. We found no experimental evidence for a similar occurrence in the 2‐w‐Z complex.

### Microhydration Dynamics

As noted, in both the 1‐w‐$Z_{N}$ and 1‐w‐$Z_{O}$ complexes, the intermolecular contact is established by a single dominant hydrogen bond, with no prevailing secondary interactions to lock the water molecule in a fixed and steady position. As such, the reorientation of the water molecule is possible for both topologies and expected to be enabled at a low energetic cost.

**Figure 6:**
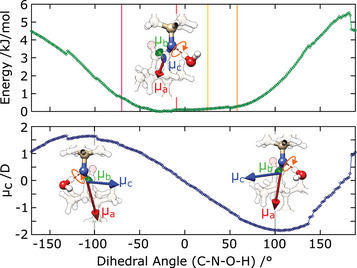
Internal dynamics of $H_{2}O$ in 1‐w‐$Z_{N}$. Relaxed energy scan and variation of the c‐component of the dipole moment with the CN–OH dihedral angle. The vertical colored lines mark the positions, in regards to the dihedral angle, of the multiple minimum structures found for 1‐w‐$Z_{N}$ (see Table S7).

In an attempt to understand the energetic landscape surrounding the docking site and to investigate the possible microhydration dynamics for 1‐w‐$Z_{N}$, we performed a relaxed energy scan (scan of CN−OH dihedral angle, 360 in 1.5 steps, top panel of Figure 6). At first, we expected to find several equilibrium states in a multi‐well potential surface surrounding the CN moiety. Surprisingly, the scan unveiled a notably wide potential well, where the reaction coordinate spans over 100 without a significant change in electronic energy, as seen in Figure 6, top panel, suggesting that the water molecule may easily engage in a dynamic re‐orientational motion at the site.

It is important to note that the fully relaxed scan only restrains the CN−OH dihedral angle (Fig 4, panel (d), labeled atoms used for the dihedral angle) and thus the molecule is free to roam and survey neighbouring locations. This motion is captured in the video of the scan trajectory that is given in the Supporting Information. The information revealed by the relaxed scan is thus incomplete, delivering only a partial picture of the energy surface at the docking site. However, it functions as strong evidence for a microsolvation dynamic behavior at the imine docking bay.

Simultaneously, we investigated the variation of the c‐component of the electric dipole moment, $\mu_{c}$, with the reaction coordinate defined in the relaxed scan, Figure 6, bottom panel. We found a pronounced change from +1.5 to −1.5 Debye as the dihedral coordinate varies from $-100^{\circ }$ to $+100^{\circ }$, crossing zero approximately at the center of the potential well, in a sinusoidal‐like behavior. Although there are multiple equilibrium structures of 1‐w‐$Z_{N}$ that yield a large $\mu_{c}$ (Table S7), there are no observable c‐type lines in the corresponding experimental spectrum. This strongly suggests that our experiment captures the rotational signatures of the vibrationally averaged structure within this wide potential well, instead of the individual signatures of the several water positions. This is further confirmed when we solve the Kraitchman equations using isotopic information (see Tables S3 and S4 in Supporting information and panel (d) of Figure 4). The best match for the experimentally derived water‐oxygen position is the conformer that is closest to the center of the potential energy well (Table S7), with $\mu_{c}∼0$ D.

The topology of 1‐w‐$Z_{O}$ also has the appropriate features that suggest that a dynamic microsolvated description is required to model the observed spectral lines. In fact, when we study the spectral characteristics for the 1‐w‐$Z_{O}$ hydrate, we conclude that c‐type lines are also absent, despite theoretical predictions anticipating a considerable electric dipole moment for that inertial axis. Additionally, there is considerable disagreement between theory and experiment with respect to the quadrupole coupling constants. This discrepancy is primarily for the constant involving the cc‐component of the tensor and, as such, is likely to emerge from the same dynamical process which is causing the absence of c‐type transitions in the rotational spectrum.

**Figure 7:**
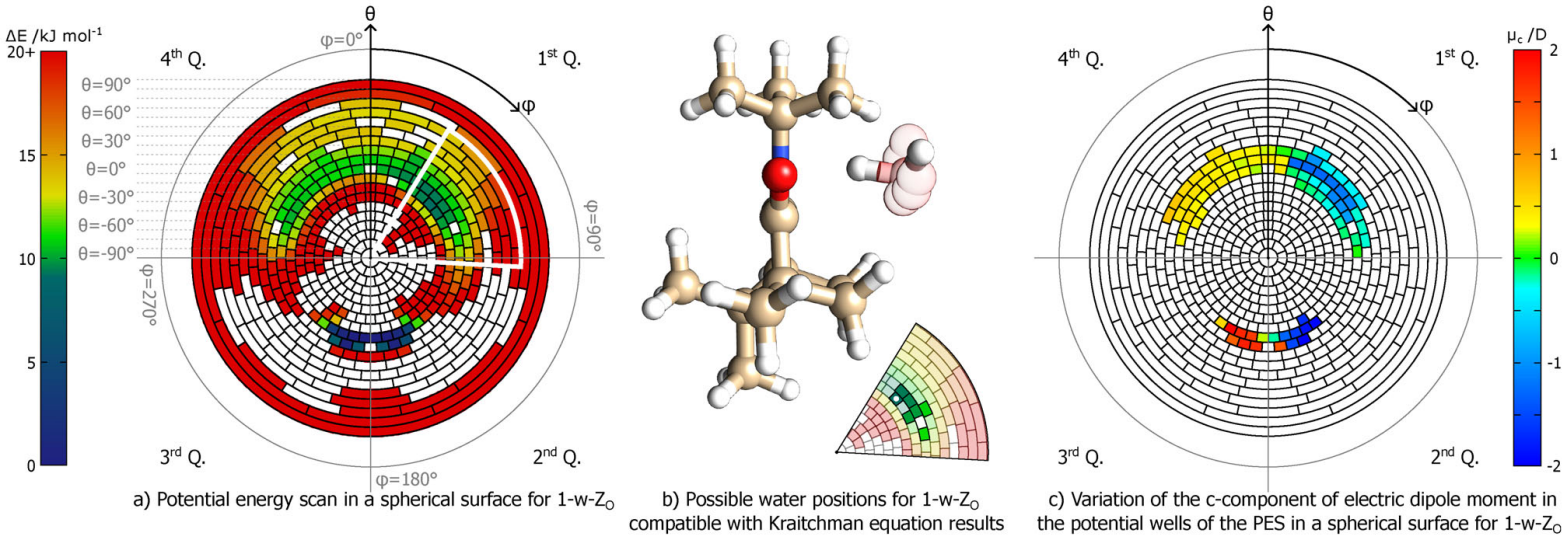
Internal dynamics of $H_{2}O$ in 1‐w‐$Z_{O}$. The angles specify the two rotations around the O atom of the imine applied to the water molecule. The first, characterized by θ, is a rotation within the plane containing the O and N atoms of the imine along the C atoms, which link them. The second, characterized by φ, is in the perpendicular plane to the first, which is parallel to the N−O line. The four quadrants of the reference system presented are identified. a) Potential energy surface sampled on a spherical surface around the O atom of the imine. The energy values presented are relative to the minimum value obtained during the scan. White regions of the scan indicate points where the water molecule intersects with the imine or where the calculations failed during the constraint imposition. The sector presented in (b) is also marked in the scan. b) Theoretical positions of the water molecule compatible with the Kraitchman equations results. A portion of the scan is shown indicating the compatible structures (non‐transparent sectors) along with the minimum energy structure of 1‐w‐$Z_{O}$ (marked by a white dot). c) Variation of the c‐component of the electric dipole moment as a function of the two angles, for the structures which are within the potential wells presented in the scan (all structures below 12.5 kJ ${\mathrm{mol}}^{-1}$).

A relaxed energy scan for 1‐w‐$Z_{O}$ was performed using a similar strategy as previously, and the resulting energetic map is depicted in Figure 7. We chose to plot the energy as a function of the angles of two applied rotations (see full description in the Supporting Information) to better visualize the energy landscape for this isomer, while at the same time incorporating the coordinates of the 1‐w‐$Z_{N}$ topology, to facilitate the discussion hereon.

In a brief overview, the regions of the sphere (Figure 7) with angles of φ close to zero indicate conformations where the water molecule is connected to the oxygen atom (1‐w‐$Z_{O}$), while regions close to 180 inform on the energetics when the water molecule docks with the nitrogen site. The global minimum structure for 1‐w‐$Z_{N}$ is captured in the dark‐blue region close to φ = 180. In the upper part of the sphere projection, in green, a clear double‐welled region, symmetric with respect to the N–C–O plane, emerges in the heat map, depicting the regions of lower energy for the 1‐w‐$Z_{O}$ isomer. As expected, the chiral subspace created by the camphor moiety makes the two wells asymmetric, and we see that the well on the first quadrant is favored. The “pizza” slice region in the figure highlights the grid points for a series of water positions, which are close to the minimum energy structure of the 1‐w‐$Z_{O}$, with a selection of geometries close to the results of the Kraitchman analysis being depicted in Figure 7, panel (b). The less‐transparent orientation in this figure is the one found to be compatible with the results of the Kraitchman analysis. Finally, we note that, similarly to what occurs in the 1‐w‐$Z_{N}$ isomer, hopping between the wells in the first and fourth quadrants would also generate a transient c‐type dipole moment component. To highlight this effect, the variation of the c‐component of the electric dipole moment for the local minima regions is shown in Figure 7, panel (c). The selected minima, highlighted in panel (b) of Figure 7, are predicted to have weak c‐type transitions in the experimental spectrum, in full agreement with our observations.

We note that the local hydration dynamics discussed here for both 1‐w‐Z topologies goes firmly in line with a recent study on the complexation between water and 1‐phenyl‐2,2,2‐trifluoroethanol (PhTFE) using MRR spectroscopy,^[^ ^67^ ^]^ where the authors found barrierless large amplitude motions (LAMs) connecting three local minima, yielding partially‐unlocked water molecules.

## Conclusions

In this study, we employed high‐resolution broadband rotational spectroscopy and quantum‐chemistry calculations to investigate the energetic balance between open and closed equilibrium state geometries of a molecular photoswitch. The switch comprises a chiral subspace created by a camphorquinone moiety and a photo‐switchable imine subunit. Previous experimental observations in solution, as well as in cryogenically cooled matrices, have shown a bias toward stabilization in the open configuration, whereas vacuum DFT calculations anticipate a favorable closed conformation.

In this work, we pursued new insights from gas‐phase studies on size‐controlled molecular complexes, creating a framework to validate the previous condensed‐phase observations, and integrate them consistently to describe the structural behavior of these imine‐based architectures when engaging in the first steps of solvation. Our experiments confirmed a favored energetic balance towards the closed (Z) monomeric form in vacuum, contradicting the condensed‐phase trend and validating DFT predictions. Stronger spectral signatures are clearly observed for Z, and we quantitatively determined that Z is substantially more abundant than the isolated E form. This energetic balance is, however, altered with the build‐up of the micro‐hydration layer.

The assignment of the first hydrated species for both Z and E isomers in the experimental spectra allowed us to confidently use the matching DFT predictions to explore the intermolecular contacts established by the water. Surprisingly, we found that with the formation of the first water complex, an increase in stability occurs for the E isomer relatively to Z. The structural models for these complexes suggest that, while the primary interaction in 1‐w‐$Z_{N}$ and 1‐w‐E is the same, the stronger secondary interactions established in 1‐w‐E steer the docking toward a more stable configuration. Importantly, because of the looser docking of water in the 1‐w‐$Z_{N}$ topology, quantum‐chemistry calculations predict a shallow potential energy surface with respect to the OH−N axial coordinate. Experimental evidence extracted from our rotational spectra clearly points to a vibrationally averaged structure enclosing several orientations of the water molecule.

For both the Z and E first‐order hydrates, the lowest energy configurations are formed via hydrogen bonding with the nitrogen site. Additional signatures uncovered during the fitting procedure led to the identification of a third species with fitted spectroscopic parameters consistent with the molecular size of a first‐order hydrate. Using an original theoretical strategy to pinpoint plausible locations for the water that would generate a matching set of rotational constants, we unveiled a water complex established with the closed (Z) form of the switch, one that instead of using the nitrogen as anchoring site is trapped in the antipodal oxygen site. We find that this third topology is also subject to considerable microhydration dynamics, where the lack of strong secondary interactions to tightly anchor the water molecule results in a double‐well potential energy surface that allows the water to roam around the C = O moiety. Such scenario is again better described as a vibrationally averaged structure enclosing several water orientations. Interconversion between $Z_{N}$ and $Z_{O}$ during the collisional cooling is likely prohibited due to the high energy barrier (∼30 kJ ${\mathrm{mol}}^{-1}$), resulting in a trapped state with considerably higher energy (∼10 kJ ${\mathrm{mol}}^{-1}$).

Moreover, observation of such trapped high energy state for the closed (Z) species informs on the predictable inability of the water to trigger the opening of the camphorquinone imine during the adiabatic expansion, and conclusively, Z → E isomerisation is found to be unlikely in these experimental conditions. These exploratory experiments will be revisited in future measurements by employing optical excitations to induce transitional motion between switch states. We note that, although isomerisation does not seem to be triggered by collisions with a single water molecule, we do find evidence that water causes an effective energy reordering of conformations, reversing the initial balance of Z/E abundances. This effect is greatly amplified with the buildup of the cluster size, prominently observed for the two‐water complex, rendering the double‐hydrate with Z undetectable.

Comparable solvent‐induced structural modifications have been documented in crystalline environments,^[^ ^68^ ^]^ prompting the hypothesis that a minimum threshold for solvent polarity may exist, below which the reversal of stability is inhibited, ensuring sustained functional integrity. Such scenario would be consistent with the previous matrix experiments where a weakly polar solid environment was used for the same system studied here. Furthermore, the relative placement of partial charges in O and N local sites may result in induction effects that could have a direct correlation with solubility^[^ ^66^ ^]^ and photo‐stationary equilibria. We also expect to learn more on the role of polarity versus steric effects in this scope of heterogeneous structural behaviour from future studies using solvating agents with different dielectric constants. With future applications in biotechnology in sight, controlling the initial state of molecular switches in situ becomes increasingly more relevant. If the local solvated environment has the potential to alter and govern the conformational preference of the switch prior to any trigger or external stimulus, then these effects must be understood to widen the potential use of this class of systems in the field of molecular nanotechnology. In this framework, rotational spectroscopy is a highly appropriate technique to investigate configurational dynamics of molecular photoswitches in a controlled environment.

## Conflict of Interests

The authors declare no conflict of interest.

## Supporting information

Supporting Information

Supplemental Video 1

## Data Availability

The data that support the findings of this study are available in the Supporting Information of this article.
