# Supplementary material for: Microhydration Dynamics in Molecular Photoswitches: Equilibrium State Reconfiguration in Imine‐Based Architectures
Source: Angew Chem Int Ed Engl. 2025 Jul 7;64(31):e202506531. doi: 10.1002/anie.202506531 (PMC12304875; doi:10.1002/anie.202506531)
Supplement: Supplementary file 1 — Supporting Information [file ANIE-64-e202506531-s002.pdf]

Supporting Information: Microhydration Dynamics in Molecular Photoswitches: Equilibrium State Reconfiguration in Imine-Based Architectures

Nuno M. Campos,[a] Rita J. C. Roque,[a] Pablo Pinacho,[b],[c] Corina H. Pollok,[d] Christian Merten,[d] Pedro S. P. Silva,[a] Manuela R. Silva,[a] Melanie Schnell,*,[b],[e] and Sérgio R. Domingos*,[a]

[a] *CFisUC, Department of Physics, University of Coimbra, Coimbra 3004-516, Portugal*

[b] *Deutsches Elektronen-Synchrotron (DESY), Notkestraße 85, Hamburg 22607, Germany*

[c] *Department of Physical Chemistry and Inorganic Chemistry, IU-CINQUIMA, University of Valladolid, Paseo Belen 7, 47011 Valladolid, Spain*

[d] *Ruhr-Universität Bochum, Fakultät fur Chemie and Biochemie, Organische Chemie II, Universitätsstrasse 150, Bochum 44801, Germany*

[e] *Institut fur Physikalische Chemie, Christian-Albrechts-Universität zu Kiel, Max-Eyth-Str. 1, Kiel 24118, Germany*

* E-mail: melanie.schnell@desy.de; sergio.domingos@uc.pt

Contents

Synthesis **S-3**

Experimental Conditions **S-3**

Spectroscopic Parameters of Isotopologues **S-3**

Conformational Search **S-7**

Method S-7

Results S-8

1-w-Z$_N$ Minimum Structures **S-12**

1wZ$_O$ Dynamics **S-13**

Estimating Molecular Structure from Rotational Constants S-14

Generation of the Grid Points for the Spherical Scan S-17

Rotation around OO axis for Minimum Energy Structure of 1wZ$_O$ S-19

Relative Abundances of Species **S-21**

Lists of Rotational Transitions **S-23**

Z Isomer S-23

E Isomer S-37

1-w-Z$_N$ Cluster S-46

1-w-Z$_O$ Cluster S-51

1-w-E Cluster S-58

2-w-E Cluster S-72

^{13}C Isotopologues of the Z Isomer S-78

^{18}O Isotopologues of 1-w-Z$_N$, 1-w-Z$_O$, 1-w-E and 2-w-E S-116

References **S-141**

Synthesis

The samples of camphorquinone imine were prepared using published methods [S1] with chemicals purchased from Sigma Aldrich (Germany).

Experimental Conditions

All experiments in this study were done using the Hamburg COMPACT Spectrometer, [S2] which is a chirped-pulse Fourier transform spectrometer. [S3] A cold molecular jet introduces the molecules into the vacuum chamber at very low rotational temperatures (2 K). To create this jet, we use a pulsed nozzle (Parker General Valve Series 9) with a constant flow of Neon at stagnation pressures of 2 to 3 bar. The sample was introduced into a reservoir in the nozzle and heated to produce sufficient vapor pressure.

A 4-μs chirp spanning 2-8 GHz is generated in an arbitrary wave-form generator (AWG) and amplified in a 300-W travelling wave tube (TWT) amplifier. The chirped pulse is then broadcasted into the chamber using a horn antenna. The molecules absorb the energy from the microwave electric field and are induced to rotate coherently in phase with the incident radiation. The free induction decay (FID) of the created macroscopic polarization is then captured using a second horn antenna. The captured FIDs are recorded, co-added, and averaged.

In Figures S1 and S2 we present the two experimental spectra obtained in the process of this study. The first was obtained by heating the reservoir with the sample to 110°C, using Neon as a carrier gas with a backing pressure of 2 bar and accumulating a total of 3.8 million FID measurements (H_2O was present in the carrier gas line and so some of the hydrated species are also captured in this spectrum). The second was obtained by heating the reservoir with the sample to 110°C, using Neon as a carrier gas with a backing pressure of 3 bar, adding a mixture of $H_2{}^{16}O$ and $H_2{}^{18}O$ to the carrier gas line and accumulating a total of 3.1 million FID measurements.

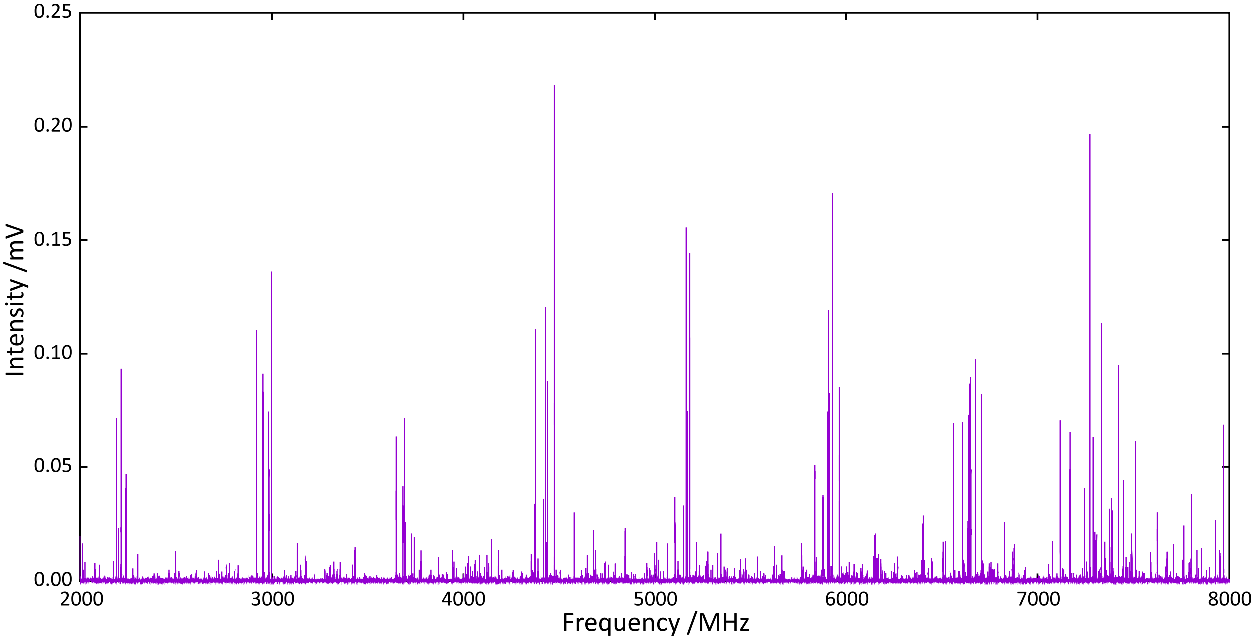

Figure S1: Rotational spectrum of camphorquinone imine in the 2 to 8 GHz range

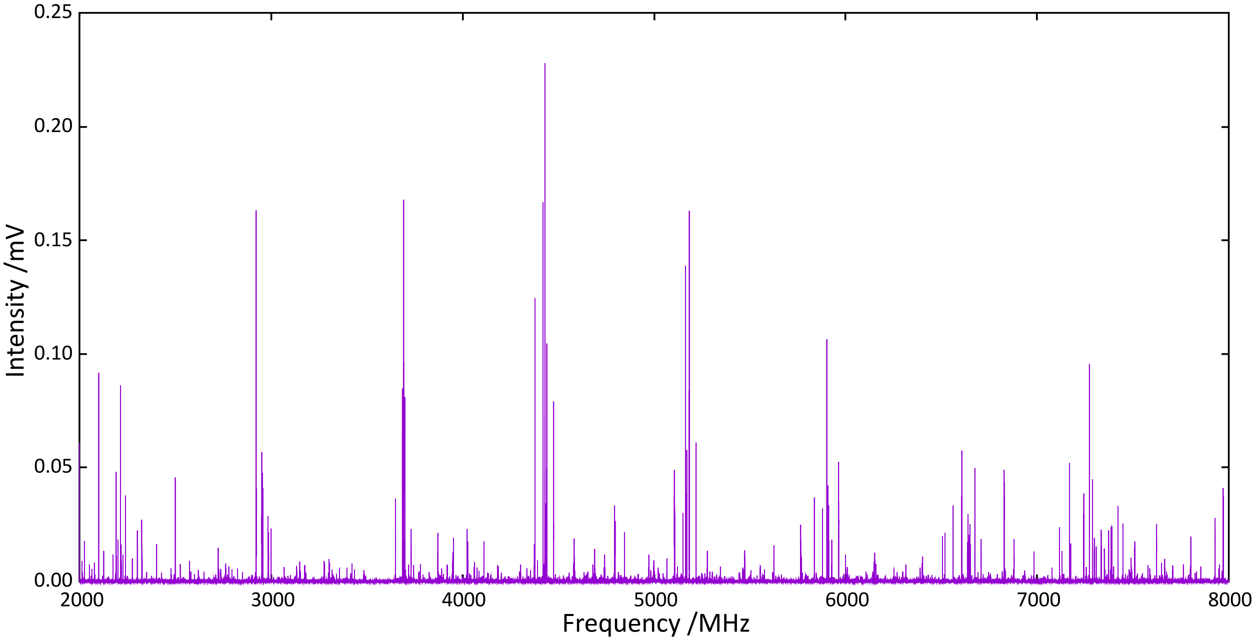

Figure S2: Rotational spectrum of camphorquinone imine co-expanded with a mixture of $H_2{}^{16}O$ and $H_2{}^{18}O$ in the 2 to 8 GHz range

Spectroscopic Parameters of Isotopologues

In this section we present information on the molecular species found in the experimental spectra but which were not presented in the main paper. This consists of the rotational

constants of the singly substituted ^{13}C isotopologues of the isolated Z form of the monomer and the singly substituted ^{18}O of the hydrated species resulting from the co-expansion of the imine with $H_2{}^{18}O$. We have also included in this section the substitution coordinates (r_s) obtained using the Kraitchman equations[S4,S5] and the isotopologue results.

In all cases, while the rotational constants of the isotopologues were allowed to vary during the fitting procedure, all other parameters (centrifugal distortion constants and quadrupole coupling constants) were fixed to the values of the parent species and not allowed to change.

In the cases of imaginary coordinates obtained from the Kraitchman equations,[S4,S5] these were assumed to be zero. This is because the values obtained are very small and the errors are on the order of magnitude as the values. Furthermore when observing the structure, these atoms are often along one of the inertial axis of the species which explains the difficulty in determining all coordinates correctly.

Table S1: Experimentally determined parameters for ^{13}C isotopologues of the Z Isomer of camphorquinone imine. Rotational constants (A, B, C in MHz) and number of lines (N) included in the fit and its standard error (σ, in kHz)

Species	A /MHz	B /MHz	C /MHz	N	σ /kHz
Parent	980.63578(17)	377.197038(55)	360.984663(51)	372	4.6568
C1	975.353(65)	375.40303(25)	360.04004(25)	75	9.5104
C2	979.503(30)	376.35176(25)	360.09527(24)	75	10.2552
C3	976.911(58)	377.00860(28)	360.33233(28)	76	8.6669
C4	972.739(75)	375.96256(26)	360.13429(26)	66	9.1438
C9	979.207(62)	376.09885(28)	359.98080(27)	59	8.5350
C10	979.021(62)	377.18975(29)	360.75230(28)	76	9.2745
C11	980.119(65)	377.16461(29)	360.90164(26)	69	9.3388
C13	977.402(74)	374.09850(37)	357.84646(36)	71	8.1732
C17	972.266(77)	375.34976(28)	360.35376(28)	60	9.1576
C21	973.666(50)	375.34098(23)	358.36186(22)	65	7.5509
C27	980.571(74)	374.98465(29)	358.95365(28)	66	9.5708
C28	976.809(64)	374.31874(29)	358.54728(26)	73	8.2298
C32	977.969(57)	372.84535(28)	356.66229(26)	78	7.5275
C36	976.218(56)	374.42955(24)	358.79275(22)	68	7.8607

Table S2: Experimentally determined coordinates (r_s structure) for the carbon atoms of the Z Isomer of camphorquinone imine.

Atoms	a /Å	b /Å	c /Å
C1	1.9066(46)	0.171(49)	1.6663(53)
C2	1.7137(24)	0.7239(59)	0.273(16)
C3	0.787(10)	1.3846(57)	0.228(36)
C4	1.3245(76)	1.2174(80)	1.6477(63)
C9	1.8797(44)	0.610(13)	0.620(14)
C10	0.197(42)	0.9296(90)	0.115(74)*i
C11	0.289(30)	0.489(17)	0.181(48)
C13	3.2902(30)	1.2070(83)	0.522(20)
C17	1.5151(68)	0.370(26)	2.0776(51)
C21	2.5631(27)	1.9200(36)	0.191(38)
C27	2.8116(35)	0.160(62)	0.09(10)
C28	2.9816(29)	0.781(11)	1.1969(73)
C32	3.9486(20)	1.1805(66)	0.200(40)
C36	2.8372(27)	0.694(11)	1.3694(56)

Table S3: Experimentally determined parameters for ^{18}O isotopologues of the water molecules in the camphorquinone imine-water complexes. Rotational constants (A, B, C in MHz) and number of lines (N) included in the fit and its standard error (σ, in kHz)

Species	A /MHz	B /MHz	C /MHz	N	σ /kHz
1-w-E	681.56455(21)	359.42981(14)	305.92344(12)	355	8.6134
1-w(^{18}O)-E	665.71747(25)	356.597695(93)	300.849741(61)	239	10.1603
1-w-Z_N	677.40586(22)	363.18760(19)	300.59866(13)	168	6.4279
1-w(^{18}O)-Z_N	658.11595(42)	361.443949(81)	295.574593(57)	145	9.2483
1-w-Z_O	596.1688(18)	369.14709(29)	296.84249(19)	184	8.5014
1-w(^{18}O)-Z_O	576.1308(18)	369.10211(13)	291.81945(10)	108	8.3723
2-w-E	550.88858(20)	334.45608(13)	283.69184(15)	166	10.5260
2-w(^{18}O,^{16}O)-E	540.96692(21)	332.34691(35)	281.35669(11)	75	10.4755
2-w(^{16}O,^{18}O)-E	542.58459(13)	331.66296(19)	279.96369(11)	78	8.7976

Table S4: Experimentally determined coordinates (r_s structure) for the oxygen atoms of the water molecules in the camphorquinone imine-water complexes.

Atoms	a /Å	b /Å	c /Å
1-w-Z_N - O40	1.80616(83)	3.33399(45)	0.029(52)
1-w-Z_O - O40	0.052(30)*i	3.83916(41)	0.3074(52)
1-w-E - O40	2.28815(66)	2.95997(51)	0.5108(30)
2-w-E - O40	1.3602(11)	2.33491(65)	1.74821(87)
2-w-E - O46	2.34630(65)	2.52536(60)	0.9005(17)

Conformational Search

Method

The molecular structure of the water complexes of the camphorquinone imine were predicted using CREST[S6] (Conformer-Rotamer Emsemble Sampling Tool) which is a utility and driver program for the semiempirical quantum chemistry package xtb.[S7]

The program takes as input a initial structure containing the monomers of the cluster whose geometry we want to predict. It searches for minima in the potential energy surface by using root-mean-square-deviation based metadynamic simulation[S8] using calculations with the GFN2-xTB[S9] semiempirical method. A bias potential is applied around already found minima to help overcome larger barriers and explore the full energy surface, or a large portion of it. An extra potential wall is added around all the molecules in the cluster which ensures that during the simulations the molecules don't completely separate.

The programs returns as output a set of molecular geometries corresponding to all found conformers (and rotamers if we want those) ordered from lower to higher energy. The semiemprical methods used greatly speed up calculations, which ensures that the search can be done in a reasonable time, but are less accurate and precise then other methods. As such, we selected the lowest energy conformers and used other methods to refine the models obtained.

DFT calculations were done in ORCA[S10–S12] using the B3LYP[S13–S16] functional, the D3BJ[S17,S18] dispersion correction and the def2-TZVP[S19,S20] basis set to calculate the geometry, energy, zero-point energy, rotational constants, electric dipole and nuclear quadrupole coupling constants of the conformers of our water clusters. The resulting output files were analysed and the conformers were organized according to their energy. We used the rotational constants and quadrupole coupling constants of the lowest energy conformer of each complex as the starting parameters in our search of the rotational spectrum.

Once the assignment of the spectrum for the one and two water clusters and their ^{18}O

isotopologues were done, we were able to determine that the predicted conformers which more closely corresponded to the obtained structures were 1-w-E_conf1 (1-w-E), 1-w-Z_conf0 (1-w-Z_N) and 2-w-E_conf2 (2-w-E). Since 1-w-E_conf1 and 1-w-Z_conf0 contained 1 imaginary frequency they did not correspond to minima. As such, we reoptimized the geometry of these two structures using tighter convergence criteria to obtain structures which correspond to a minimum in the potential energy surface. The properties of these are presented in the main paper. Later in our analysis, when we knew of the presence of 1-w-Z_O, we looked once more at our conformational search and noticed that 1-w-Z_conf3 is actually extremely similar to the minimum energy structure for 1-w-Z_O which we found later.

Results

Table S5: Predicted conformers for the one water clusters sorted on relative energy (including zero point energy corrections, in kJ/mol), their rotational constants (MHz), electric dipole moments (D), nuclear quadrupole coupling constants (MHz) and number of imaginary vibrational frequencies of the predicted structure

Name	ΔE	A	B	C	μ_a	μ_b	μ_c	χ_{bb}	χ_{cc}	χ_{aa}	Im. Freq.
1-w-E_conf1	0.00	679.61	359.82	301.20	-3.90	-5.79	0.26	-3.81	3.58	0.23	1
1-w-E_conf2	1.42	689.80	357.47	298.99	-3.93	-5.41	0.47	-3.57	3.32	0.25	0
1-w-Z_conf0	1.64	685.76	365.24	304.87	0.98	0.56	-0.05	-4.05	3.46	0.58	1
1-w-E_conf0	4.16	704.70	348.16	297.92	-3.64	-4.87	0.17	-4.13	3.79	0.35	1
1-w-Z_conf3	10.42	639.93	370.77	304.65	1.03	-0.64	0.45	-4.70	4.07	0.63	0
1-w-Z_conf7	11.52	628.86	373.13	303.45	0.68	-1.49	0.35	1.43	-2.43	1.01	0
1-w-E_conf7	11.78	620.29	338.31	272.96	2.63	-4.14	0.08	-3.31	3.97	-0.66	0
1-w-Z_conf2	11.86	627.74	369.03	298.17	-0.66	0.79	0.34	-1.98	0.93	1.06	0
1-w-E_conf9	12.21	631.58	348.09	289.60	1.90	-3.72	0.85	-3.06	3.73	-0.67	0
1-w-E_conf5	12.35	624.97	343.09	277.25	-2.17	-4.05	0.15	-3.34	3.96	-0.62	1
1-w-E_conf10	12.96	627.89	339.81	277.59	-2.57	-4.19	0.16	-3.19	3.97	-0.78	1
1-w-Z_conf8	14.47	668.55	366.97	311.16	0.81	-0.82	-0.10	3.65	-4.90	1.25	0
1-w-Z_conf11	16.06	668.45	342.06	283.21	4.50	-0.50	-0.26	-0.66	-0.41	1.08	0
1-w-Z_conf10	16.92	613.93	355.20	281.59	4.47	-1.38	0.48	-4.13	3.03	1.10	3

1-w-Z_conf5	17.07	642.60	368.20	299.99	0.38	-1.28	-0.41	-3.17	2.40	0.77	0
1-w-E_conf13	17.50	628.29	338.22	274.53	2.06	-3.62	0.19	-3.33	3.93	-0.60	2
1-w-E_conf15	17.71	608.12	339.10	272.00	-2.79	-4.21	0.10	-3.49	4.11	-0.62	2
1-w-E_conf14	19.05	635.77	338.21	281.35	1.89	-3.40	0.48	-3.46	3.94	-0.48	1
1-w-Z_conf13	24.60	753.14	285.01	259.96	4.49	-2.06	-0.47	-3.17	3.08	0.10	2
1-w-Z_conf15	24.72	861.02	267.17	260.78	5.06	0.95	-0.52	-3.21	3.69	-0.48	2
1-w-Z_conf19	24.74	796.28	281.87	266.85	-5.01	-1.38	-0.51	-3.39	3.88	-0.50	2
1-w-E_conf21	25.17	615.70	379.77	301.00	0.59	-6.39	1.00	-1.94	1.08	0.86	1
1-w-Z_conf12	25.42	795.82	284.59	267.81	-4.74	1.96	-0.10	-3.54	3.80	-0.26	0
1-w-Z_conf49	25.55	801.49	248.65	240.17	4.50	-0.24	-0.77	-3.80	2.51	1.29	2
1-w-Z_conf23	25.59	784.67	286.41	269.77	-3.74	0.49	-0.11	0.53	-1.05	0.52	0
1-w-E_conf20	25.67	610.13	377.28	297.96	0.87	-6.64	1.55	-4.62	3.86	0.75	1
1-w-Z_conf36	25.72	596.02	350.63	279.52	1.63	-2.16	-0.88	-3.35	3.24	0.11	1
1-w-E_conf18	25.81	634.02	374.04	298.30	1.93	-5.65	1.18	-3.77	2.93	0.84	0
1-w-Z_conf31	26.08	857.57	256.52	249.67	-4.01	-0.47	-0.50	-2.24	2.54	-0.30	2
1-w-Z_conf37	26.43	650.62	327.82	274.02	-2.51	-2.25	0.78	-3.72	3.87	-0.14	1
1-w-Z_conf35	26.56	890.03	255.91	251.30	3.92	0.24	-1.23	-3.36	3.85	-0.49	1
1-w-Z_conf16	26.86	665.58	301.28	261.96	4.02	-2.26	-0.34	-3.47	3.22	0.25	3
1-w-Z_conf45	27.46	878.04	246.06	245.13	-3.76	1.90	1.16	-4.05	2.79	1.26	1
1-w-Z_conf25	27.48	745.55	292.17	265.86	-3.01	0.23	-0.62	1.42	-2.35	0.92	1
1-w-Z_conf48	27.60	889.36	246.84	245.86	4.41	-0.84	-0.79	-5.18	3.98	1.20	0
1-w-Z_conf40	27.91	941.68	246.73	237.96	0.23	-0.28	1.17	-4.37	3.20	1.17	2
1-w-E_conf30	28.03	789.51	274.29	257.96	4.13	4.38	1.38	-3.13	3.37	-0.24	2
1-w-E_conf28	28.19	724.96	295.88	269.99	4.10	-5.01	-1.40	-3.07	3.26	-0.19	2
1-w-E_conf27	28.19	721.02	296.58	270.16	4.05	5.05	-1.40	-3.11	3.25	-0.14	2
1-w-Z_conf50	28.25	931.01	241.36	236.37	4.35	-0.43	1.22	-5.04	3.83	1.21	1
1-w-E_conf54	28.33	846.39	272.39	260.77	3.90	4.79	-0.78	-1.50	1.63	-0.12	1
1-w-Z_conf52	28.35	842.28	247.93	242.96	-4.46	0.55	0.36	-3.75	2.67	1.08	1
1-w-E_conf50	28.66	805.98	281.31	275.13	2.14	-3.47	1.15	-2.68	3.60	-0.92	0
1-w-E_conf39	28.75	828.35	268.76	256.80	3.47	-3.85	1.15	-2.06	2.72	-0.66	2
1-w-E_conf34	28.88	866.56	270.53	257.62	4.00	-4.71	-0.36	-3.01	3.45	-0.45	1
1-w-E_conf29	29.03	738.69	294.87	271.01	4.15	-4.92	-1.44	-3.06	3.34	-0.27	2
1-w-E_conf41	29.41	876.56	262.46	252.49	-3.30	-3.84	-0.42	-2.86	3.78	-0.92	1

1-w-E_conf36	29.47	825.83	258.54	253.87	1.83	2.48	-1.47	-1.59	1.71	-0.12	2
1-w-E_conf35	29.47	808.42	277.63	267.66	3.86	-4.47	-0.99	-1.20	1.17	0.03	2
1-w-E_conf31	29.56	746.99	288.49	269.19	4.07	4.44	1.54	-1.08	0.95	0.14	1
1-w-E_conf47	29.64	592.29	332.49	268.31	3.66	5.93	-0.65	-2.85	3.30	-0.45	2
1-w-Z_conf21	29.94	818.75	242.16	229.27	-4.79	-0.45	-0.08	-4.10	3.96	0.14	2
1-w-Z_conf47	30.41	941.71	215.69	210.54	-4.12	1.57	1.52	-3.86	2.70	1.16	2
1-w-Z_conf44	30.56	951.26	217.25	211.97	3.99	2.25	0.34	-4.34	3.16	1.18	1
1-w-E_conf22	31.39	648.10	314.86	275.49	-3.48	5.14	-1.20	-1.16	0.70	0.47	1
1-w-E_conf23	31.72	642.97	317.44	274.47	3.52	5.31	1.07	-0.74	0.28	0.46	1
1-w-E_conf46	31.90	702.81	294.41	265.60	-3.81	-4.99	-1.37	-2.71	2.67	0.04	3
1-w-Z_conf39	32.39	974.40	233.49	225.95	0.30	0.48	-0.57	-2.96	1.84	1.12	2
1-w-E_conf24	32.65	650.71	308.94	271.26	-3.25	-5.11	1.00	-1.79	1.46	0.33	2
1-w-Z_conf54	34.67	483.27	147.79	129.76	-2.24	0.60	0.52	-4.74	3.53	1.20	3

Table S6: Predicted conformers for the two water clusters sorted on relative energy (including zero point energy corrections, in kJ/mol), their rotational constants (MHz), electric dipole moments (D), nuclear quadrupole coupling constants (MHz) and number of imaginary vibrational frequencies of the predicted structure

Name	ΔE	A	B	C	μ_a	μ_b	μ_c	χ_{bb}	χ_{cc}	χ_{aa}	Im. Freq.
2-w-E_conf2	0.00	559.77	331.42	282.93	-2.52	3.07	1.64	1.12	-2.06	0.94	0
2-w-E_conf0	2.49	529.85	347.59	280.06	1.27	4.05	-1.72	-0.64	0.25	0.39	0
2-w-E_conf4	3.79	551.60	333.11	283.45	-2.91	3.78	0.74	-3.57	3.49	0.07	0
2-w-E_conf1	5.45	529.62	325.86	283.47	2.22	3.01	-1.56	-2.85	2.74	0.10	0
2-w-E_conf3	6.77	555.76	339.50	281.51	1.94	4.32	-1.60	-0.72	0.20	0.52	0
2-w-Z_conf0	11.03	495.42	353.53	259.67	-0.61	-0.52	2.13	-2.98	2.32	0.66	0
2-w-E_conf11	11.43	579.72	279.85	237.69	-6.91	3.31	-1.02	-3.79	3.48	0.31	1
2-w-Z_conf34	11.91	485.14	355.97	254.24	-0.02	0.07	0.47	-2.54	1.89	0.65	0
2-w-Z_conf8	11.97	460.00	358.52	248.84	0.05	0.54	-0.89	-3.84	3.20	0.64	0
2-w-Z_conf4	12.91	458.46	358.91	247.27	0.02	0.43	1.04	-4.00	3.32	0.67	1
2-w-Z_conf5	12.98	536.54	321.45	267.08	2.48	1.07	-1.55	-4.33	3.48	0.85	0
2-w-E_conf18	13.19	545.78	322.02	291.59	-4.39	4.81	-0.38	-2.53	2.42	0.11	0
2-w-Z_conf27	13.72	572.36	296.28	256.77	-3.36	-1.13	-1.95	-4.63	3.44	1.20	0
2-w-E_conf12	14.18	546.70	327.72	276.52	-3.70	5.84	-0.79	-3.35	3.28	0.07	1

2-w-E_conf5	14.20	458.40	361.69	262.32	-1.13	4.99	-1.62	-3.35	3.61	-0.26	0
2-w-E_conf7	14.88	460.17	348.65	251.41	-4.00	5.00	-2.17	-3.34	3.62	-0.28	0
2-w-Z_conf15	14.99	550.66	325.41	272.78	-1.55	1.62	-2.36	-4.26	3.33	0.93	0
2-w-E_conf6	15.38	460.07	352.35	254.48	-3.70	5.08	-2.20	-3.66	3.81	-0.14	0
2-w-Z_conf12	15.63	535.87	333.87	273.05	-0.84	2.34	2.16	-2.82	2.03	0.79	0
2-w-E_conf8	15.69	461.63	360.34	260.51	-4.31	4.82	-0.28	-3.58	3.80	-0.22	0
2-w-E_conf25	16.34	459.53	345.01	247.19	4.65	-4.76	-2.03	-3.60	3.69	-0.09	0
2-w-Z_conf26	16.40	599.98	282.59	245.98	-3.25	-1.82	-1.74	-4.66	3.45	1.21	0
2-w-Z_conf6	16.96	545.60	289.04	239.04	3.20	-0.89	1.78	-3.97	3.01	0.96	1
2-w-E_conf22	19.55	463.83	353.66	253.58	2.90	-4.44	1.29	-1.83	1.47	0.36	2
2-w-E_conf9	19.56	465.73	352.07	253.25	3.03	4.49	1.27	-2.03	1.65	0.38	2
2-w-E_conf20	20.66	467.58	355.66	258.85	-0.28	4.23	1.09	-1.07	0.61	0.46	1
2-w-E_conf14	20.84	458.79	332.59	237.35	-6.84	4.02	-0.16	-3.15	3.32	-0.16	0
2-w-E_conf23	21.07	510.35	342.53	271.38	2.80	3.60	0.74	-2.12	1.83	0.29	2
2-w-Z_conf7	21.07	584.91	290.28	249.75	-2.55	-1.31	1.77	-2.31	1.40	0.91	0
2-w-E_conf13	21.49	478.51	352.25	264.08	-3.37	4.27	0.74	-2.35	2.27	0.08	0
2-w-E_conf28	21.53	503.86	312.80	260.34	1.13	-4.04	-0.93	-3.17	3.48	-0.32	0
2-w-E_conf21	22.33	484.74	352.66	261.84	-0.37	4.44	1.03	-1.54	1.39	0.15	1
2-w-Z_conf11	23.03	470.12	346.49	268.95	-1.23	0.17	0.55	-1.18	-0.10	1.27	1
2-w-E_conf17	23.27	521.16	277.88	231.23	1.89	3.06	-0.14	-0.59	0.78	-0.20	0
2-w-Z_conf22	23.52	456.18	327.19	243.51	0.24	1.47	1.25	3.59	-4.89	1.30	0
2-w-Z_conf18	23.54	462.35	353.40	266.29	-2.13	-1.03	-1.06	0.95	-2.09	1.14	0
2-w-Z_conf21	23.60	496.18	346.43	277.62	-2.18	0.11	1.21	3.34	-4.69	1.35	0
2-w-Z_conf10	23.77	436.59	341.02	252.40	-1.23	-1.06	-1.11	-3.09	2.33	0.76	0
2-w-E_conf27	24.09	490.96	328.06	241.03	0.88	5.72	-0.24	-2.48	2.24	0.24	3
2-w-E_conf15	24.44	501.13	284.30	229.30	2.93	-3.39	0.34	-2.49	3.57	-1.08	1
2-w-Z_conf19	24.82	440.65	343.81	256.97	-1.08	-1.63	-1.60	-3.70	2.72	0.98	0
2-w-Z_conf16	24.88	460.72	352.31	265.97	1.87	-1.10	-1.23	2.05	-3.12	1.07	0
2-w-E_conf24	25.07	452.70	354.21	255.33	-2.77	4.93	-0.35	-3.71	3.91	-0.20	0
2-w-Z_conf25	25.47	469.10	324.65	244.30	-0.07	1.19	1.51	3.61	-4.78	1.17	0
2-w-Z_conf9	25.66	475.78	348.50	270.42	-1.60	0.38	0.42	1.81	-2.75	0.94	0
2-w-E_conf34	25.68	506.85	331.23	246.18	-0.65	5.32	0.67	-1.51	1.19	0.32	2
2-w-Z_conf24	26.51	435.86	345.63	238.14	-0.67	-1.16	-0.53	-3.73	3.35	0.38	2

2-w-E_conf29	27.51	511.54	272.40	224.42	2.60	-3.13	0.09	-1.39	1.79	-0.40	0
2-w-Z_conf23	27.53	527.98	298.73	235.34	-0.49	-0.51	-0.52	2.42	-3.98	1.56	0
2-w-Z_conf13	27.71	458.45	316.39	235.96	0.88	2.18	-1.59	4.00	-5.20	1.20	0
2-w-Z_conf17	28.13	436.92	326.31	234.89	-0.47	-2.31	-0.60	3.62	-4.87	1.25	1
2-w-Z_conf14	28.31	433.05	327.47	232.55	0.54	1.79	-1.70	3.93	-4.94	1.01	0
2-w-E_conf37	28.94	503.06	312.33	259.06	0.85	-4.18	-1.10	-3.24	3.53	-0.28	2
2-w-E_conf26	28.98	506.94	286.62	236.95	-1.77	-2.70	0.09	-2.71	3.96	-1.26	1
2-w-Z_conf31	29.13	480.57	336.77	244.69	-0.95	0.67	1.16	-2.88	1.79	1.09	2
2-w-Z_conf43	30.29	554.18	318.08	281.94	-2.35	-0.20	2.21	-3.91	2.60	1.31	0
2-w-Z_conf47	30.58	569.47	323.26	292.92	-2.31	0.79	2.06	-4.08	2.78	1.30	0
2-w-Z_conf51	30.80	462.80	289.71	213.69	-1.98	-1.39	0.09	-4.36	3.44	0.91	3
2-w-Z_conf40	31.03	562.46	263.47	215.54	0.44	-1.24	-0.77	-4.67	3.59	1.08	2
2-w-Z_conf36	31.07	482.70	318.98	250.83	0.60	-2.31	-1.01	-1.99	1.12	0.88	2
2-w-Z_conf42	32.06	551.45	282.24	243.22	-0.62	-1.31	1.90	-4.87	3.51	1.37	0
2-w-Z_conf45	35.53	458.22	345.88	240.42	-1.86	3.64	-1.96	4.18	-5.16	0.98	1
2-w-Z_conf55	35.78	456.64	334.35	235.04	-2.92	-3.83	0.92	3.35	-4.56	1.21	2
2-w-Z_conf59	35.83	635.41	254.72	228.86	4.25	-0.57	-0.62	-3.63	3.39	0.25	3
2-w-Z_conf33	36.09	478.70	344.01	249.95	-1.61	-2.25	-2.42	2.65	-3.75	1.10	1
2-w-Z_conf56	36.65	620.42	257.59	233.62	4.57	0.46	-2.96	-3.96	3.28	0.68	1
2-w-Z_conf52	41.29	391.02	303.71	209.37	-1.37	-2.14	-1.45	3.78	-4.74	0.96	0

1-w-Z_N Minimum Structures

As is noted in the main paper we found multiple equilibrium structures for the 1-w-Z_N species. Many of them were found in the process of attempting to obtained a true minimum structure for this species, since a relatively small change in the initial structure sometimes resulted in a large difference of the minimum structure obtained. Table S7 presents the theoretical parameters of four minimum structures that were found along with a transition state structure, which has the best agreement with the Kraitchman equation results. Of the four minimum structures, the one that was initially found from the re-optimization of

1-w-Z_conf0 and used for the starting parameters of the fit was 1-w-Z_N[4].

Table S7: Structures obtained from reoptimizations of the 1-w-Z_N geometry. Relative energy (including zero point energy corrections, in kJ/mol), rotational constants (MHz), electric dipole moments (D) and nuclear quadrupole coupling constants (MHz) of four local minima and the transition state which best agrees with experimental parameters and Kraitchman substitution coordinates

Parameters	Minimum Structures				Transition Structure
	1-w-Z_N[1] (red)	1-w-Z_N[2] (yellow)	1-w-Z_N[3] (orange)	1-w-Z_N[4] (pink)	1-w-Z_N (best fit)
CNOH angle /°	-9.8	+24.9	+57.7	-70.6	+0.8
A /MHz	685.61	685.95	686.97	695.21	685.87
B /MHz	362.66	362.59	362.94	360.21	362.87
C /MHz	301.69	301.74	302.62	304.51	301.91
μ_a /D	1.1	1.1	1.4	1.7	1.0
μ_b /D	0.6	0.6	-0.4	0.2	0.6
μ_c /D	0.2	-0.6	-1.4	1.6	-0.0
χ_{aa} /MHz	0.62	0.62	0.61	0.59	0.62
$\chi_{bb} - \chi_{cc}$ /MHz	-7.47	-7.44	-7.45	-7.60	-7.46
Δ E /kJ/mol	0.0	0.2	0.5	1.7	0.1

1wZ_O Dynamics

In this section we will discuss the methods we used to find and study the dynamics of the 1-w-Z_O species. As noted in the main paper, the search for 1-w-Z_O began from a spectral misfit early on the fitting process of the two first-order microsolvated species. This resulted in spectral fit for an unknown species whose rotational constants could not be matched to any theoretical prediction from DFT, but which were on the correct order of magnitude to be another one-water complex.

This unknown species became far more relevant when the question of if a single water molecule can induce a conformational change during the collision events occurring in the supersonic jet expansion arose since the presence or absence of another mono-hydrated species in the rotational spectrum could be a important piece of information to support or refute this hypothesis.

As such, we attempted to reverse engineer the problem and use the experimental rotational constants to obtain candidate structures for this species. Given the rotational constants, we assumed that this unknown was also a cluster composed of the imine and one water molecule.

Estimating Molecular Structure from Rotational Constants

As was referenced in the main paper we used a innovative methodology to obtain a starting point for an unknown molecular structure. This method attempts to reverse-engineer possible molecular structures which correspond to experimentally determined rotational constants. It mostly relies on the fact the inertia tensor for a molecular cluster can be broken into multiple components which are independent of each other.

We can consider the center of mass of our molecule cluster as the origin of our reference system with no loss of generality. For a molecule M in our cluster, the its contribution to the tensor of inertia is given by:

$$I_{ij}^{M} = \sum_{k \in M} m_k \left(||\vec{r}^{\,(k)}||^2 \delta_{ij} - r_i^{(k)} r_j^{(k)} \right) \tag{1}$$

Where $\vec{r}^{\,(k)}$ is the position of atom k in molecule M and m_k is the mass of atom k in molecule M. The position of atom k can always be written as the sum of the position of the center of mass of M and the position of atom k relative to this center of mass:

$$\vec{r}^{\,(k)} = \vec{R}^{\,CM,M} + \vec{r}^{\,(k),rel} \tag{2}$$

Substituting this expression into the equation above we can, with some algebraic manipulation, break the inertia tensor into two distinct components, one which depends only on the center of mass coordinates and another which depends only on the relative coordinates:

$$I_{ij}^{M} = I_{ij}^{M,CM} + I_{ij}^{M,rel} \tag{3}$$

$$I_{ij}^{M,CM} = M_M \left(||\vec{R}^{\ CM,M}||^2 \delta_{ij} - R_i^{CM,M} R_j^{CM,M} \right) \tag{4}$$

$$I_{ij}^{M,rel} = \sum_{k \in M} m_k \left(||\vec{r}^{\ (k),rel}||^2 \delta_{ij} - r_i^{(k),rel} r_j^{(k),rel} \right) \tag{5}$$

Where M_M represents the total mass of molecule M. This separation of the inertia tensor can be made for each molecule of a cluster but there are some other factors that must be taken into account.

Since a translation or rotation of the entire molecular system results in no alteration to the inertia tensor relative to the center of mass of the system, the inertia tensor will depend only on the relative positions and orientations of the components. In this way, terms like $I_{ij}^{M,CM}$ will depend purely on the relative positions of the fragments, while terms like $I_{ij}^{M,rel}$ will depend on the geometry of the fragments and the relative orientations.

The case of a cluster formed from 2 molecules, like the one we are studying, allows even further simplifications. In order to facilitate the calculations we can fix the position and orientation of one of the molecules and vary the position and orientation of the other, until we find values which are compatible with the experimental results. Since we desired only a rough estimate of the position of the water molecule and considering its much smaller size and mass compared to the imine, and so much smaller contribution to the inertia tensor, instead of searching for the possible orientations of the water molecule we searched only for the position of the center of mass of the water. As such, we decided to average the contribution of the water molecule to the inertia tensor over all its possible orientations. Doing so will only affect the $I_{ij}^{M,rel}$ term which will instead assume the following form:

$$I_{ij,avg}^{M,rel} = \sum_{k \in M} \frac{2}{3} m_k ||\vec{r}^{\ (k),rel}||^2 \delta_{ij} \tag{6}$$

The program we wrote do do this search relied on a brute force algorithm. It took as an input two geometries, one for a molecule which would remain fixed and another which would be moved. In our case these were the Z or E isomer of the imine and a water molecule, respectively.

The total inertia tensor of the system can then be given by:

$$I_{ij} = I_{ij}^{rel} + I_{ij}^{CM} \tag{7}$$

$$I_{ij}^{rel} = I_{ij}^{Z/E,rel} + I_{ij,avg}^{H_2O,rel} \tag{8}$$

$$I_{ij}^{CM} = I_{ij}^{Z/E,CM} + I_{ij}^{H_2O,CM} \tag{9}$$

Since we are averaging over all orientations for the water molecule, the first term only has to be calculated once. The second term, which depends on the positions of the centers of mass of the imine and water, has to be calculated everytime we change the relative position between the two.

We then defined the parameters of our search, defining a range of possible possible values for the coordinates of the center of mass of the water molecule and a step to create a grid of points to test. At each point, the inertia tensor relative to the center of mass of the cluster was calculated, diagonalized to obtain the principal moments of inertia and the results compared to the experimental values. The structures were ranked based on the sum of the squared differences between the predicted and experimental values and only the 32 best positions were saved.

The search was ran initially with a larger range of about 16 Å centered on the center of mass of the imine and a larger step of about 0.25 Å. The obtained structures were inspected, any which were not sensible were immediately discarded (for example any intersecting structure or those where the water molecule didn't have a available bonding site) and the remaining ones were then refined by using a smaller range centered on the best positions of the previous search and a step of about 0.1 Å. These searches were ran using both the E and Z isomer of the imine. In the tighter search, many of the structures obtained were effectively the same, with only slight shifts of the position of the water. In this case, one example of each of these structures was taken and then optimized with ORCA [S10–S12] using DFT calculations at the B3LYP-D3BJ/def2-TZVP [S13–S20] level of theory to obtain the

closest equilibrium structure to the starting structure and check if it was compatible with the experimental results.

A couple of notes and warnings regarding the method. First of all, is the fact that the method requires one to assume the composition of a cluster. In some cases, like this one, a reasonable assumption of the cluster composition can be made but in other cases it might not be possible. Second, the geometries used during the search are those of the molecules in isolation, which means that no relaxation of the molecular geometry can be taken into account. In a case where the molecules used are quite rigid this will not pose a problem, but in a more flexible system, where alterations to the molecular geometry to facilitate binding are more important or relevant, this method is likely to have a larger error. Finally, in the case of averaging the contribution of a molecule over all its orientations we are effectively replacing the molecule with a sphere of uniform density with mass M_M and radius $R = \sqrt{5(I_{xx} + I_{yy} + I_{zz})/6M_M}$.When the molecule whose orientation is being averaged is highly symmetric (the closer to spherical the better) or much smaller in size and mass than the other molecule, the error introduced by this process is not large since the change in orientation of the molecule does not have a significant effect on the inertia tensor. For highly asymmetric molecules or molecules closer in size the error introduced by this process will likely be much larger.

Applications of this method to similar problems in the future require testing to accurately judge the accuracy and limitations of the method.

Generation of the Grid Points for the Spherical Scan

The points used for the scan presented in Figure 7 of the main paper were generated by creating a set of pairs of angles in such a way that the area around each of the points (as defined below) was approximately the same. This pair of angles characterizes two rotations around the O atom of the imine which were applied to the water molecule to generate the geometries which were then used in the calculations.

First we defined a uniformly distributed set of n+1 values for the angle ϑ which is related to the first rotation. The first rotation will occur in the plane containing the N and O atoms of the imine and the C atoms which connect them. The true rotation which we apply will be given by $\theta = \vartheta - \frac{\pi}{2}$ but the derivations were initially done using ϑ. The difference between the two is simply what position is considered the zero as can be seen in Fig. S3, panel (c).

$$\vartheta_i = \frac{\pi}{n} i \quad , \quad i = 0, ..., n \tag{10}$$

In his way we will have a set of latitude values for our spherical surface. The next rotation will occur in a plane perpendicular to the first which is also parallel to the N-O line. This rotation will be characterized for by the angle φ, which is undefined for the extreme values of ϑ ($\vartheta = 0$ and $\vartheta = \pi$). As such, for the poles we will consider only one value possible for φ, which will be zero. For all other values of latitude, the number of longitudinal points will be chosen so that the area around each point, as defined below, is the same (or as close as possible). The longitudinal angles will be given by the following formula, where m_i is the number of longitudinal points for ϑ_i (except for ϑ_0 and ϑ_n where $m_0 = m_n = 1$):

$$\varphi_{i,j} = \frac{2\pi}{m_i} j \quad , \quad j = 0, ..., m_i - 1 \tag{11}$$

For the poles the area around the points is considered the portion of the spherical surface described by $\vartheta \in \left[0, 0 + \frac{\vartheta_1}{2}\right[$ and $\varphi \in [0, 2\pi[$ for the "north" pole and $\vartheta \in \left]\pi - \frac{\theta_{n-1}}{2}, \pi\right]$ and $\varphi \in [0, 2\pi[$ for the "south" pole. These two areas will be equal to each other.

For all other points, the area around the points was considered to be the region of the spherical surface in between $\vartheta \in \left[\frac{\vartheta_{i-1}+\vartheta_i}{2}, \frac{\vartheta_i+\vartheta_{i+1}}{2}\right[$ and $\varphi \in \left[\varphi_{i,j} - \frac{\pi}{m_i}, \varphi_{i,j} + \frac{\pi}{m_i}\right[$. The areas for every point at the same latitude are the same since the longitudinal points are distributed uniformly at a given latitude.

The expression for these areas can be written in the following form:

$$A_{pole} = \int_0^{\frac{\pi}{2n}} \sin(\vartheta) d\vartheta \int_0^{2\pi} d\varphi = 2\pi \left(1 - \cos\left(\frac{\pi}{2n}\right)\right) \tag{12}$$

$$A_i = \int_{\frac{\pi}{2n}(2i-1)}^{\frac{\pi}{2n}(2i+1)} \sin(\vartheta) d\vartheta \int_{-\frac{\pi}{m_i}}^{\frac{\pi}{m_i}} d\varphi = \frac{4\pi}{m_i} \sin\left(\frac{\pi}{n} i\right) \sin\left(\frac{\pi}{2n}\right) \quad , \quad i = 1, ..., n-1 \tag{13}$$

And the expression of the area of the points at a given latitude can be equaled to the area at the poles to give an expression for m_i, which is rounded to the closest integer since the number of longitudinal points must be a integer:

$$m_i = \frac{\sin\left(\frac{\pi}{2n}\right)}{1 - \cos\left(\frac{\pi}{2n}\right)} \sin\left(\frac{\pi}{n} i\right) \quad , \quad i = 1, ..., n-1 \tag{14}$$

We then took a initial state which you can see in Figure S3 and applied two rotations to generate all the geometries that we used in our scan. These geometries were optimized with DFT calculations at the B3LYP-D3BJ/def2-TZVP [S13–S20] level of theory using ORCA [S10–S12] while imposing two constraints which ensure that the angles θ and φ defined below remained fixed. An additional filtering step was added to the process to remove any structure where the imine and water intersected. This was done by setting a minimum distance between atoms that all pairs of atoms had to obey.

Something to note is that the points generated by this method are not distributed in a spherically symmetrical way. Instead for a large number of points they approach cylindrical symmetry with the axis of the second rotation as the axis of symmetry. Furthermore, the way that the φ values are generated means that there is a greater number of points close to $\varphi = 0$. Both of these problems could probably be mitigated by the addition of a latitude dependent offset to the longitude values, however the introduced bias from the method was actually helpful to us since the primary objective was to scan the potential energy surface close the O atom of the imine and, as such, was left in intentionally and not corrected.

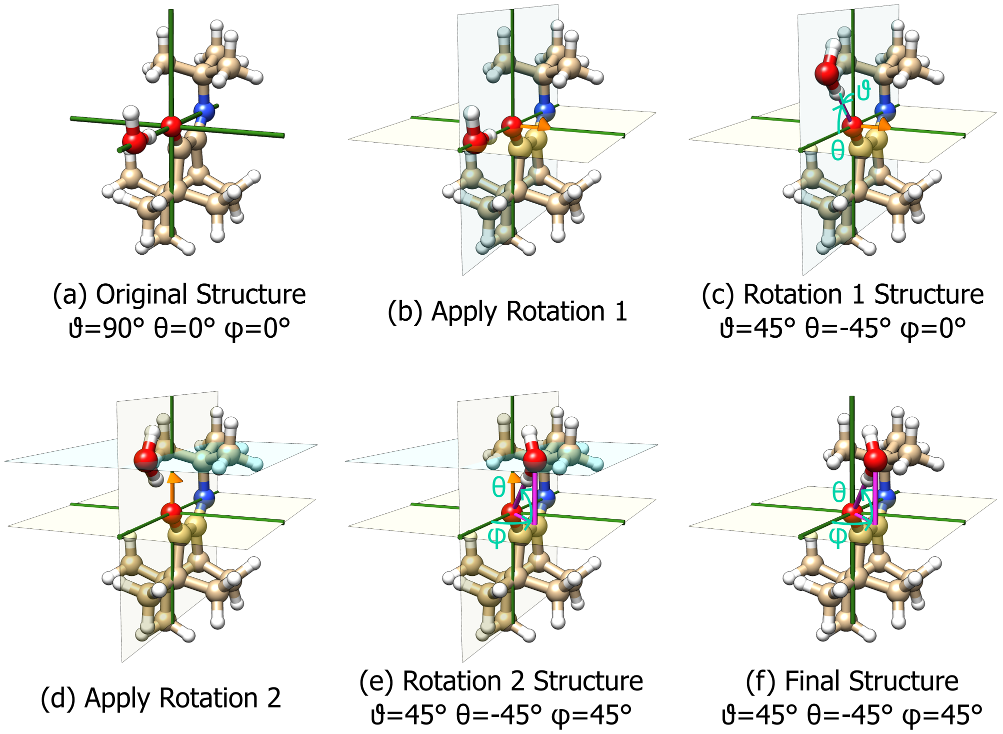

Figure S3: Process of generation of the geometries for the spherical scan in Figure 7 of the main paper (for a $\theta = -45°$ and $\varphi = 45°$ rotation). In all panels, a set of three green axis are shown which can be used as a reference for the water position. In panels (b)-(f), the yellow planes serve only as a reference and to aid in the definition of angles. In panels (b)-(e), the orange arrow marks the rotation axis and the blue plane the rotation plane. In panels (c), (e) and (f) the angles used in this section and the main paper are defined.

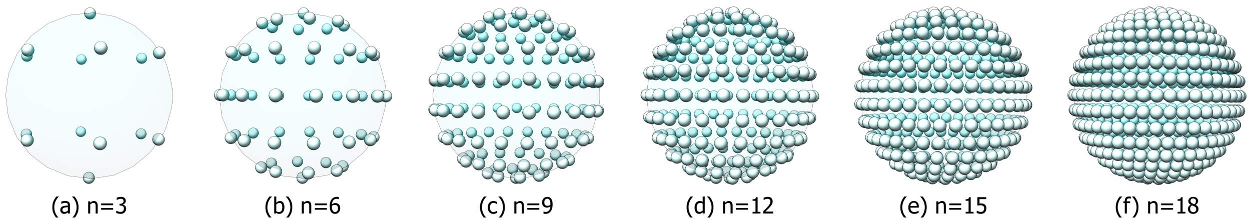

Figure S4: Distribution of grid points generated on a spherical surface for different values of latitudinal divisions. The distribution shown in panel (f) with n=18 was the one used for in the scan presented in Fig. 7 of the main paper.

Rotation around OO axis for Minimum Energy Structure of 1wZ$_O$

To gain a better understanding of the PES of 1-w-Z$_O$, we ran a relaxed surface scan similar to the one done in Figure 6 of the main paper for 1-w-Z$_N$. We scanned the potential energy surface as the water molecule is rotated through the axis which connects the O atom of the

water to the O atom of the imine. As can be seen we have a single wide potential well similar to what is seen in Figure 6 of the main paper, but with a smaller range. The c-component of the dipole moment also changes sinusoidally in this region, similar to what is observed for the 1-w-Z_N complex.

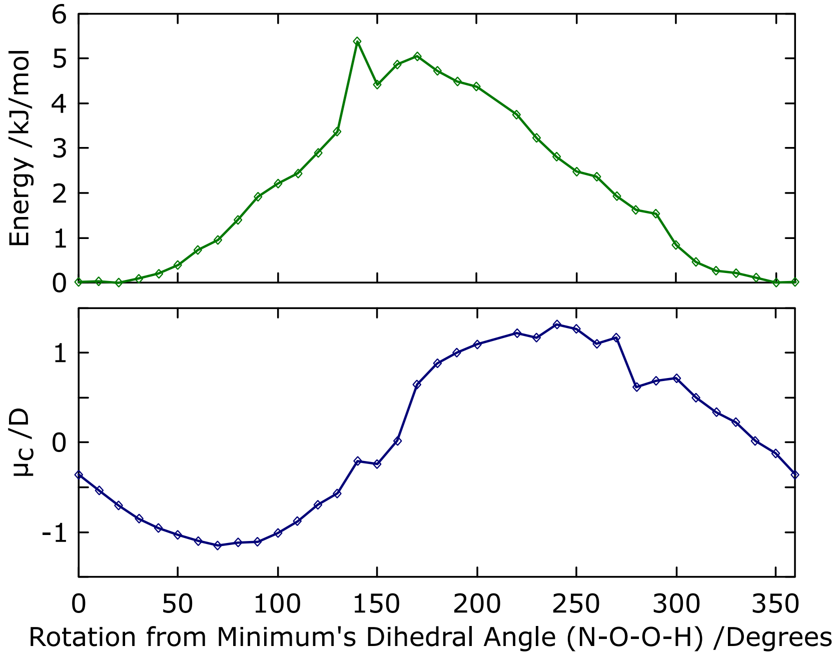

Figure S5: Relaxed energy scan and variation of the c-component of the dipole moment as we rotate the water molecule around the OO axis for 1wZ_O.

Relative Abundances of Species

The variation of the electric dipole moment components for the Z isomer hydrated species makes it difficult to accurately calculate the relative abundances of these species, since the intensity of rotational transitions is proportional to the square of the electric dipole moment components which generates it and the abundance of the species. As such, unlike the main paper, where we were able to estimate that the relative abundance of the isolated isomers was of 88% Z and 12% E, we can't obtain a precise value for the relative abundance of the species when considering all of them.

However, while a precise value is impossible to calculate, we can still estimate a range

of possible values for the relative abundances since we know the range of variation of the electric dipole components for both the Z isomer hydrated species. For 1-w-Z_N, μ_a is about 1 Debye near the center of the potential well, while it approaches 2 Debye as we move towards the edges of the potential well in any direction. For 1-w-Z_O, μ_a varies between slightly below 1 Debye and slightly above 2 Debye in the potential wells. As such, we calculated the relative abundance assuming these extreme cases of either 1 or 2 Debye for these species, obtaining 4 values of relative abundance for each species which gives us the ranges of possible abundances which can be seen in Fig. S6.

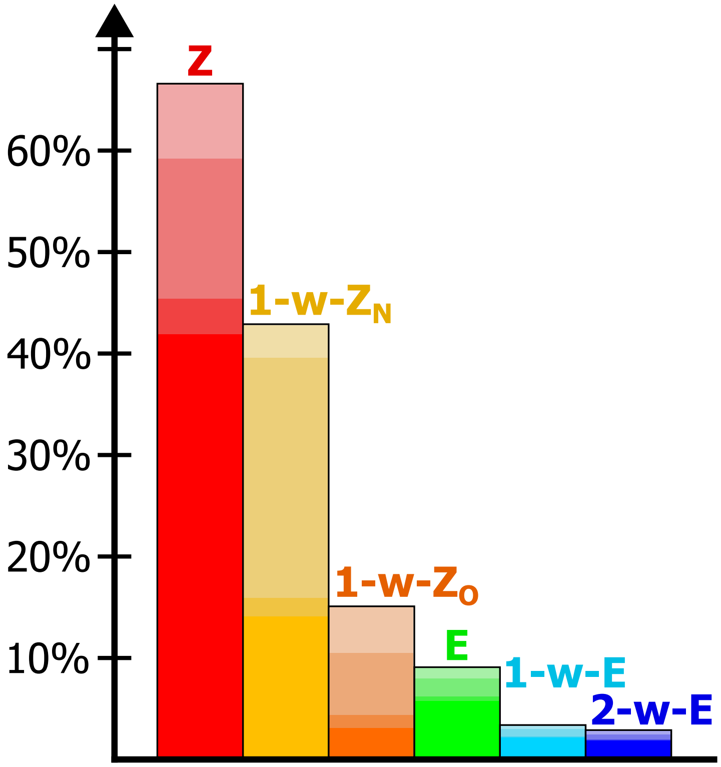

Figure S6: Relative abundance of the isomers of the imine switch obtained using the relative intensity of the lines and predicted electric dipole moments. Due to uncertainty in the components of the electric dipole moment of the monohydrated Z isomers, we calculated the abundances assuming 1 or 2 Debye for the a-component. As such, we present 4 values in the bar, with the minimum possible abundance showed in solid color and growing more transparent with increasing abundance.

Lists of Rotational Transitions

Z Isomer

Table S8: Observed and calculated rotational transitions (MHz) for the Z Isomer

Observed	Calculated	Obs-Calc	J'	K_a'	K_c'	F'	J"	K_a"	K_c"	F"
2063.8984	2063.8943	0.0041	2	1	2	3	1	0	1	2
2188.9609	2188.9656	-0.0047	3	1	3	3	2	1	2	3
2189.8038	2189.8025	0.0013	3	1	3	2	2	1	2	1
2212.8360	2212.8378	-0.0018	3	0	3	2	2	0	2	2
2213.2486	2213.2577	-0.0091	3	0	3	4	2	0	2	3
2213.3312	2213.3358	-0.0046	3	0	3	2	2	0	2	1
2213.5398	2213.5349	0.0049	3	0	3	3	2	0	2	3
2214.2627	2214.2674	-0.0047	3	2	2	2	2	2	1	1
2214.8151	2214.8162	-0.0011	3	2	2	3	2	2	1	2
2215.5264	2215.5290	-0.0026	3	2	1	2	2	2	0	1
2215.7382	2215.7388	-0.0006	3	2	1	4	2	2	0	3
2236.5452	2236.5443	0.0009	3	1	2	2	2	1	1	2
2238.5833	2238.5846	-0.0013	3	1	2	4	2	1	1	3
2238.7181	2238.7202	-0.0021	3	1	2	3	2	1	1	2
2238.8412	2238.8406	0.0006	3	1	2	2	2	1	1	1
2384.4299	2384.4309	-0.0010	4	0	4	3	3	1	3	2
2384.8626	2384.8638	-0.0012	4	0	4	5	3	1	3	4
2776.5160	2776.5150	0.0010	3	1	3	3	2	0	2	2
2777.9218	2777.9169	0.0049	3	1	3	4	2	0	2	3
2918.6008	2918.6007	0.0001	4	1	4	4	3	1	3	4
2919.5833	2919.5869	-0.0036	4	1	4	3	3	1	3	2

2919.6840	2919.6828	0.0012	4	1	4	4	3	1	3	3
2919.6840	2919.6867	-0.0027	4	1	4	5	3	1	3	4
2949.4573	2949.4582	-0.0009	4	0	4	4	3	0	3	3
2949.5321	2949.5230	0.0091	4	0	4	5	3	0	3	4
2949.5750	2949.5699	0.0051	4	0	4	3	3	0	3	2
2952.4054	2952.3993	0.0061	4	2	3	3	3	2	2	2
2952.4345	2952.4364	-0.0019	4	2	3	5	3	2	2	4
2952.5785	2952.5789	-0.0004	4	2	3	4	3	2	2	3
2953.1500	2953.1412	0.0088	4	3	2	3	3	3	1	2
2953.1656	2953.1647	0.0009	4	3	1	3	3	3	0	2
2953.2774	2953.2720	0.0054	4	3	2	5	3	3	1	4
2953.6224	2953.6177	0.0047	4	3	2	4	3	3	1	3
2953.6444	2953.6424	0.0020	4	3	1	4	3	3	0	3
2955.2948	2955.2955	-0.0007	10	3	7	10	10	2	8	10
2955.5918	2955.5823	0.0095	4	2	2	3	3	2	1	2
2955.6256	2955.6300	-0.0044	4	2	2	5	3	2	1	4
2955.8433	2955.8469	-0.0036	4	2	2	4	3	2	1	3
2955.9478	2955.9554	-0.0076	10	3	7	11	10	2	8	11
2982.4105	2982.4105	0.0000	4	1	3	3	3	1	2	3
2984.4553	2984.4513	0.0040	4	1	3	5	3	1	2	4
2984.5161	2984.5076	0.0085	4	1	3	4	3	1	2	3
2984.5868	2984.5863	0.0005	4	1	3	3	3	1	2	2
3011.3711	3011.3673	0.0038	8	3	5	8	8	2	6	8
3011.8785	3011.8834	-0.0049	8	3	5	9	8	2	6	9
3011.9452	3011.9486	-0.0034	8	3	5	7	8	2	6	7
3029.1570	3029.1494	0.0076	7	3	4	7	7	2	5	7
3029.6024	3029.6004	0.0020	7	3	4	8	7	2	5	8

3029.6711	3029.6657	0.0054	7	3	4	6	7	2	5	6
3041.7736	3041.7803	-0.0067	6	3	3	7	6	2	4	7
3041.8595	3041.8531	0.0064	6	3	3	5	6	2	4	5
3049.2008	3049.2023	-0.0015	5	3	2	5	5	2	3	5
3049.5853	3049.5825	0.0028	5	3	2	6	5	2	3	6
3058.8890	3058.8828	0.0062	4	3	2	5	4	2	3	5
3063.6830	3063.6806	0.0024	6	3	4	6	6	2	5	6
3063.7215	3063.7205	0.0010	6	3	4	7	6	2	5	7
3063.7215	3063.7274	-0.0059	6	3	4	5	6	2	5	5
3068.6236	3068.6317	-0.0081	7	3	5	6	7	2	6	6
3068.6400	3068.6360	0.0040	7	3	5	8	7	2	6	8
3068.6567	3068.6667	-0.0100	7	3	5	7	7	2	6	7
3075.9190	3075.9148	0.0042	8	3	6	7	8	2	7	7
3075.9190	3075.9265	-0.0075	8	3	6	9	8	2	7	9
3076.0176	3076.0204	-0.0028	8	3	6	8	8	2	7	8
3086.1751	3086.1815	-0.0064	9	3	7	10	9	2	8	10
3086.3321	3086.3352	-0.0031	9	3	7	9	9	2	8	9
3100.0100	3100.0190	-0.0090	10	3	8	11	10	2	9	11
3100.2128	3100.2137	-0.0009	10	3	8	10	10	2	9	10
3149.3869	3149.3924	-0.0055	5	0	5	4	4	1	4	3
3149.6945	3149.6969	-0.0024	5	0	5	6	4	1	4	5
3150.9067	3150.9069	-0.0002	5	0	5	5	4	1	4	4
3319.2736	3319.2704	0.0032	2	2	0	3	1	1	1	2
3482.9827	3482.9827	-0.0000	4	1	4	4	3	0	3	3
3484.3443	3484.3460	-0.0017	4	1	4	5	3	0	3	4
3484.7247	3484.7260	-0.0013	4	1	4	3	3	0	3	2
3647.9146	3647.9180	-0.0034	5	1	5	5	4	1	4	5

3648.9716	3648.9689	0.0027	5	1	5	4	4	1	4	3
3650.3327	3650.3349	-0.0022	5	1	5	4	4	1	4	4
3684.2825	3684.2829	-0.0004	5	0	5	4	4	0	4	4
3684.4320	3684.4315	0.0005	5	0	5	5	4	0	4	4
3684.5202	3684.5199	0.0003	5	0	5	6	4	0	4	5
3684.5435	3684.5484	-0.0049	5	0	5	4	4	0	4	3
3684.6430	3684.6439	-0.0009	5	0	5	5	4	0	4	5
3689.9848	3689.9856	-0.0008	5	2	4	4	4	2	3	4
3690.1643	3690.1690	-0.0047	5	2	4	6	4	2	3	5
3690.1643	3690.1631	0.0012	5	2	4	4	4	2	3	3
3690.2375	3690.2341	0.0034	5	2	4	5	4	2	3	4
3690.3730	3690.3762	-0.0032	5	2	4	5	4	2	3	5
3691.4427	3691.4464	-0.0037	5	4	2	4	4	4	1	3
3691.4427	3691.4468	-0.0041	5	4	1	4	4	4	0	3
3691.5363	3691.5381	-0.0018	5	4	1	6	4	4	0	5
3691.5363	3691.5377	-0.0014	5	4	2	6	4	4	1	5
3691.8402	3691.8493	-0.0091	5	4	2	5	4	4	1	4
3691.8402	3691.8496	-0.0094	5	4	1	5	4	4	0	4
3691.9976	3692.0034	-0.0058	5	3	2	6	4	3	1	5
3692.1998	3692.1910	0.0088	5	3	2	5	4	3	1	4
3696.1395	3696.1473	-0.0078	5	2	3	4	4	2	2	4
3696.5418	3696.5324	0.0094	5	2	3	4	4	2	2	3
3696.5528	3696.5486	0.0042	5	2	3	6	4	2	2	5
3696.7063	3696.7075	-0.0012	5	2	3	5	4	2	2	4
3697.0123	3697.0145	-0.0022	5	2	3	5	4	2	2	5
3918.4154	3918.4183	-0.0029	6	0	6	5	5	1	5	4
3918.6438	3918.6462	-0.0024	6	0	6	7	5	1	5	6

3919.7653	3919.7679	-0.0026	6	0	6	6	5	1	5	5
4023.7144	4023.7131	0.0013	3	2	2	3	2	1	1	2
4025.1909	4025.1901	0.0008	3	2	2	4	2	1	1	3
4026.0124	4026.0115	0.0009	3	2	2	2	2	1	1	1
4074.1862	4074.1835	0.0027	3	2	1	2	2	1	2	1
4074.8424	4074.8388	0.0036	3	2	1	4	2	1	2	3
4076.0543	4076.0505	0.0038	3	2	1	3	2	1	2	2
4183.8393	4183.8463	-0.0070	5	1	5	6	4	0	4	5
4184.1195	4184.1249	-0.0054	5	1	5	4	4	0	4	3
4377.9651	4377.9619	0.0032	6	1	6	5	5	1	5	4
4377.9728	4377.9690	0.0038	6	1	6	6	5	1	5	5
4377.9891	4377.9962	-0.0071	6	1	6	7	5	1	5	6
4417.8661	4417.8651	0.0010	6	0	6	6	5	0	5	5
4427.3784	4427.3781	0.0003	6	2	5	5	5	2	4	5
4427.6207	4427.6244	-0.0037	6	2	5	7	5	2	4	6
4427.6207	4427.6267	-0.0060	6	2	5	5	5	2	4	4
4427.6528	4427.6532	-0.0004	6	2	5	6	5	2	4	5
4427.8579	4427.8603	-0.0024	6	2	5	6	5	2	4	6
4429.7375	4429.7417	-0.0042	6	5	1	5	5	5	0	4
4429.7375	4429.7417	-0.0042	6	5	2	5	5	5	1	4
4429.8127	4429.8090	0.0037	6	5	2	7	5	5	1	6
4429.8127	4429.8090	0.0037	6	5	1	7	5	5	0	6
4430.0913	4430.0877	0.0036	6	4	2	5	5	4	1	4
4430.0913	4430.0863	0.0050	6	4	3	5	5	4	2	4
4430.0913	4430.0903	0.0010	6	5	1	6	5	5	0	5
4430.0913	4430.0903	0.0010	6	5	2	6	5	5	1	5
4430.1230	4430.1265	-0.0035	6	4	2	7	5	4	1	6

4430.1230	4430.1250	-0.0020	6	4	3	7	5	4	2	6
4430.3097	4430.3128	-0.0031	6	4	2	6	5	4	1	5
4430.3097	4430.3113	-0.0016	6	4	3	6	5	4	2	5
4430.7045	4430.6952	0.0093	6	3	4	5	5	3	3	4
4430.7103	4430.7109	-0.0006	6	3	4	7	5	3	3	6
4430.8289	4430.8249	0.0040	6	3	4	6	5	3	3	5
4430.9196	4430.9172	0.0024	6	3	3	5	5	3	2	4
4430.9196	4430.9292	-0.0096	6	3	3	7	5	3	2	6
4431.0422	4431.0457	-0.0035	6	3	3	6	5	3	2	5
4438.7275	4438.7237	0.0038	6	2	4	5	5	2	3	4
4438.7275	4438.7313	-0.0038	6	2	4	7	5	2	3	6
4438.8697	4438.8717	-0.0020	6	2	4	6	5	2	3	5
4473.0423	4473.0418	0.0005	6	1	5	5	5	1	4	5
4475.0138	4475.0167	-0.0029	6	1	5	7	5	1	4	6
4475.0138	4475.0159	-0.0021	6	1	5	6	5	1	4	5
4475.0780	4475.0749	0.0031	6	1	5	5	5	1	4	4
4476.7054	4476.7050	0.0004	6	1	5	6	5	1	4	6
4690.1276	4690.1297	-0.0021	7	0	7	6	6	1	6	5
4690.3034	4690.3050	-0.0016	7	0	7	8	6	1	6	7
4691.3273	4691.3298	-0.0025	7	0	7	7	6	1	6	6
4737.5695	4737.5719	-0.0024	4	2	3	4	3	1	2	3
4739.0409	4739.0419	-0.0010	4	2	3	5	3	1	2	4
4739.5697	4739.5702	-0.0005	4	2	3	3	3	1	2	2
4839.9640	4839.9633	0.0007	4	2	2	3	3	1	3	2
4840.4249	4840.4212	0.0037	4	2	2	5	3	1	3	4
4841.8132	4841.8103	0.0029	4	2	2	4	3	1	3	3
4876.0659	4876.0662	-0.0003	6	1	6	6	5	0	5	5

4877.3231	4877.3225	0.0006	6	1	6	7	5	0	5	6
4877.5380	4877.5384	-0.0004	6	1	6	5	5	0	5	4
5105.3911	5105.3939	-0.0028	7	1	7	7	6	1	6	7
5107.8608	5107.8613	-0.0005	7	1	7	6	6	1	6	6
5149.5303	5149.5309	-0.0006	7	0	7	7	6	0	6	6
5149.6506	5149.6550	-0.0044	7	0	7	8	6	0	6	7
5149.6702	5149.6732	-0.0030	7	0	7	6	6	0	6	5
5164.7573	5164.7539	0.0034	7	2	6	8	6	2	5	7
5164.7573	5164.7584	-0.0011	7	2	6	6	6	2	5	5
5164.7573	5164.7623	-0.0050	7	2	6	7	6	2	5	6
5168.0847	5168.0851	-0.0004	7	6	1	8	6	6	0	7
5168.0847	5168.0851	-0.0004	7	6	2	8	6	6	1	7
5168.3482	5168.3495	-0.0013	7	5	2	8	6	5	1	7
5168.3482	5168.3406	0.0076	7	6	1	7	6	6	0	6
5168.3482	5168.3406	0.0076	7	6	2	7	6	6	1	6
5168.3482	5168.3494	-0.0012	7	5	3	8	6	5	2	7
5168.5305	5168.5309	-0.0004	7	5	2	7	6	5	1	6
5168.5305	5168.5308	-0.0003	7	5	3	7	6	5	2	6
5168.8323	5168.8311	0.0012	7	4	3	8	6	4	2	7
5168.8323	5168.8262	0.0061	7	4	4	8	6	4	3	7
5168.9517	5168.9489	0.0028	7	4	4	7	6	4	3	6
5168.9517	5168.9539	-0.0022	7	4	3	7	6	4	2	6
5169.6670	5169.6626	0.0044	7	3	5	6	6	3	4	5
5169.6670	5169.6695	-0.0025	7	3	5	8	6	3	4	7
5169.7518	5169.7484	0.0034	7	3	5	7	6	3	4	6
5170.1676	5170.1625	0.0051	7	3	4	6	6	3	3	5
5170.1676	5170.1748	-0.0072	7	3	4	8	6	3	3	7

5170.2584	5170.2634	-0.0050	7	3	4	7	6	3	3	6
5181.6419	5181.6417	0.0002	7	2	5	6	6	2	4	6
5182.3501	5182.3547	-0.0046	7	2	5	8	6	2	4	7
5182.3501	5182.3499	0.0002	7	2	5	6	6	2	4	5
5182.4875	5182.4903	-0.0028	7	2	5	7	6	2	4	6
5183.0983	5183.0966	0.0017	7	2	5	7	6	2	4	7
5217.5859	5217.5863	-0.0004	7	1	6	6	6	1	5	6
5219.5188	5219.5151	0.0037	7	1	6	8	6	1	5	7
5272.1720	5272.1754	-0.0034	3	3	1	2	2	2	0	1
5272.5188	5272.5146	0.0042	3	3	0	4	2	2	1	3
5443.3023	5443.2984	0.0039	5	2	4	5	4	1	3	4
5444.7665	5444.7597	0.0068	5	2	4	6	4	1	3	5
5445.1521	5445.1470	0.0051	5	2	4	4	4	1	3	3
5463.0254	5463.0253	0.0001	8	0	8	7	7	1	7	6
5463.1704	5463.1700	0.0004	8	0	8	9	7	1	7	8
5464.0779	5464.0841	-0.0062	8	0	8	8	7	1	7	7
5502.3900	5502.3921	-0.0021	7	5	2	7	7	4	3	7
5502.5998	5502.6048	-0.0050	7	5	3	8	7	4	4	8
5502.5998	5502.5981	0.0017	7	5	2	8	7	4	3	8
5564.7347	5564.7274	0.0073	7	1	7	7	6	0	6	6
5565.9041	5565.9030	0.0011	7	1	7	8	6	0	6	7
5566.0837	5566.0811	0.0026	7	1	7	6	6	0	6	5
5616.9094	5616.9088	0.0006	5	2	3	4	4	1	4	3
5617.2850	5617.2831	0.0019	5	2	3	6	4	1	4	5
5618.8360	5618.8350	0.0010	5	2	3	5	4	1	4	4
5834.6172	5834.6117	0.0055	8	1	8	8	7	1	7	7
5834.6250	5834.6281	-0.0031	8	1	8	7	7	1	7	6

5834.6570	5834.6521	0.0049	8	1	8	9	7	1	7	8
5879.2812	5879.2807	0.0005	8	0	8	8	7	0	7	7
5879.4217	5879.4180	0.0037	8	0	8	9	7	0	7	8
5901.5000	5901.5066	-0.0066	8	2	7	9	7	2	6	8
5901.5000	5901.5019	-0.0019	8	2	7	8	7	2	6	7
5906.6041	5906.5955	0.0086	8	7	1	8	7	7	0	7
5906.6041	5906.5955	0.0086	8	6	2	9	7	6	1	8
5906.6041	5906.5955	0.0086	8	7	2	8	7	7	1	7
5906.6041	5906.5955	0.0086	8	6	3	9	7	6	2	8
5906.7696	5906.7698	-0.0002	8	6	3	8	7	6	2	7
5906.7696	5906.7698	-0.0002	8	6	2	8	7	6	1	7
5906.9548	5906.9552	-0.0004	8	5	3	7	7	5	2	6
5906.9548	5906.9551	-0.0003	8	5	4	7	7	5	3	6
5906.9707	5906.9730	-0.0023	8	5	3	9	7	5	2	8
5906.9707	5906.9729	-0.0022	8	5	4	9	7	5	3	8
5907.1007	5907.0983	0.0024	8	5	3	8	7	5	2	7
5907.1007	5907.0982	0.0025	8	5	4	8	7	5	3	7
5907.6724	5907.6722	0.0002	8	4	4	7	7	4	3	6
5907.6724	5907.6816	-0.0092	8	4	4	9	7	4	3	8
5907.6724	5907.6681	0.0043	8	4	5	9	7	4	4	8
5907.7651	5907.7554	0.0097	8	4	5	8	7	4	4	7
5907.7651	5907.7692	-0.0041	8	4	4	8	7	4	3	7
5908.7964	5908.7947	0.0017	8	3	6	7	7	3	5	6
5908.7964	5908.7971	-0.0007	8	3	6	9	7	3	5	8
5908.8627	5908.8556	0.0071	8	3	6	8	7	3	5	7
5909.7909	5909.7973	-0.0064	8	3	5	9	7	3	4	8
5909.7909	5909.7936	-0.0027	8	3	5	7	7	3	4	6

5909.8669	5909.8672	-0.0003	8	3	5	8	7	3	4	7
5927.5113	5927.5107	0.0006	8	2	6	7	7	2	5	6
5927.5113	5927.5143	-0.0030	8	2	6	9	7	2	5	8
5927.6475	5927.6492	-0.0017	8	2	6	8	7	2	5	7
5963.2974	5963.3036	-0.0062	8	1	7	8	7	1	6	7
5963.3592	5963.3659	-0.0067	8	1	7	7	7	1	6	6
5967.3663	5967.3642	0.0021	10	1	9	10	9	2	8	9
6009.5343	6009.5366	-0.0023	4	3	2	4	3	2	1	3
6009.7324	6009.7301	0.0023	4	3	2	5	3	2	1	4
6009.7769	6009.7876	-0.0107	4	3	2	3	3	2	1	2
6011.2396	6011.2454	-0.0058	4	3	1	4	3	2	2	3
6140.9726	6140.9715	0.0011	6	2	5	6	5	1	4	5
6142.4267	6142.4247	0.0020	6	2	5	7	5	1	4	6
6142.7317	6142.7295	0.0022	6	2	5	5	5	1	4	4
6235.6207	6235.6258	-0.0051	9	0	9	8	8	1	8	7
6235.7300	6235.7336	-0.0036	9	0	9	10	8	1	8	9
6236.5387	6236.5419	-0.0032	9	0	9	9	8	1	8	8
6249.8013	6249.8082	-0.0069	8	1	8	8	7	0	7	7
6250.8941	6250.9000	-0.0059	8	1	8	9	7	0	7	8
6251.0295	6251.0360	-0.0065	8	1	8	7	7	0	7	6
6406.6703	6406.6636	0.0067	6	2	4	5	5	1	5	4
6406.9984	6406.9911	0.0073	6	2	4	7	5	1	5	6
6408.7046	6408.7027	0.0019	6	2	4	6	5	1	5	5
6562.2311	6562.2243	0.0068	9	1	9	9	8	1	8	8
6562.2655	6562.2628	0.0027	9	1	9	10	8	1	8	9
6607.0688	6607.0694	-0.0006	9	0	9	9	8	0	8	8
6607.2148	6607.2157	-0.0009	9	0	9	10	8	0	8	9

6607.2325	6607.2286	0.0039	9	0	9	8	8	0	8	7
6637.8335	6637.8317	0.0018	9	2	8	10	8	2	7	9
6637.8335	6637.8364	-0.0029	9	2	8	8	8	2	7	7
6644.8533	6644.8524	0.0009	9	8	2	9	8	8	1	8
6644.8533	6644.8524	0.0009	9	8	1	9	8	8	0	8
6645.0110	6645.0176	-0.0066	9	7	3	9	8	7	2	8
6645.0110	6645.0176	-0.0066	9	7	2	9	8	7	1	8
6645.1616	6645.1542	0.0074	9	6	3	8	8	6	2	7
6645.1616	6645.1542	0.0074	9	6	4	8	8	6	3	7
6645.1616	6645.1707	-0.0091	9	6	3	10	8	6	2	9
6645.1616	6645.1707	-0.0091	9	6	4	10	8	6	3	9
6645.2901	6645.2959	-0.0058	9	6	3	9	8	6	2	8
6645.2901	6645.2959	-0.0058	9	6	4	9	8	6	3	8
6645.6909	6645.6873	0.0036	9	5	5	8	8	5	4	7
6645.6909	6645.6976	-0.0067	9	5	4	10	8	5	3	9
6645.6909	6645.6876	0.0033	9	5	4	8	8	5	3	7
6645.6909	6645.6973	-0.0064	9	5	5	10	8	5	4	9
6645.7814	6645.7891	-0.0077	9	5	4	9	8	5	3	8
6645.7814	6645.7888	-0.0074	9	5	5	9	8	5	4	8
6646.6769	6646.6678	0.0091	9	4	6	8	8	4	5	7
6646.7299	6646.7367	-0.0068	9	4	6	9	8	4	5	8
6648.0927	6648.0862	0.0065	9	3	7	8	8	3	6	7
6648.0927	6648.0867	0.0060	9	3	7	10	8	3	6	9
6649.9121	6649.9149	-0.0028	9	3	6	10	8	3	5	9
6649.9121	6649.9130	-0.0009	9	3	6	8	8	3	5	7
6649.9776	6649.9772	0.0004	9	3	6	9	8	3	5	8
6674.2012	6674.1970	0.0042	9	2	7	8	8	2	6	7

6674.2012	6674.1999	0.0013	9	2	7	10	8	2	6	9
6674.3377	6674.3338	0.0039	9	2	7	9	8	2	6	8
6706.3000	6706.2908	0.0092	9	1	8	9	8	1	7	8
6725.6059	6725.6023	0.0036	8	6	2	8	8	5	3	8
6725.6059	6725.6024	0.0035	8	6	3	8	8	5	4	8
6745.7887	6745.7954	-0.0067	5	3	3	5	4	2	2	4
6746.0279	6746.0204	0.0075	5	3	3	6	4	2	2	5
6746.0770	6746.0818	-0.0048	5	3	3	4	4	2	2	3
6750.8622	6750.8575	0.0047	5	3	2	5	4	2	3	4
6781.4605	6781.4597	0.0008	11	1	10	10	10	2	9	9
6781.6008	6781.5993	0.0015	11	1	10	12	10	2	9	11
6830.7234	6830.7179	0.0055	7	2	6	7	6	1	5	6
6832.1637	6832.1619	0.0018	7	2	6	8	6	1	5	7
6832.4136	6832.4129	0.0007	7	2	6	6	6	1	5	5
6932.7442	6932.7518	-0.0076	9	1	9	9	8	0	8	8
6933.7510	6933.7448	0.0062	9	1	9	10	8	0	8	9
6933.8553	6933.8526	0.0027	9	1	9	8	8	0	8	7
7006.5075	7006.5081	-0.0006	10	0	10	9	9	1	9	8
7006.5927	7006.5923	0.0004	10	0	10	11	9	1	9	10
7007.2912	7007.2885	0.0027	10	0	10	10	9	1	9	9
7211.0558	7211.0517	0.0041	7	2	5	6	6	1	6	5
7211.3513	7211.3496	0.0017	7	2	5	8	6	1	6	7
7213.2263	7213.2239	0.0024	7	2	5	7	6	1	6	6
7289.3254	7289.3262	-0.0008	10	1	10	10	9	1	9	9
7289.3629	7289.3566	0.0063	10	1	10	9	9	1	9	8
7289.3729	7289.3667	0.0062	10	1	10	11	9	1	9	10
7332.9731	7332.9709	0.0022	10	0	10	10	9	0	9	9

7333.1272	7333.1215	0.0057	10	0	10	11	9	0	9	10
7333.1272	7333.1322	-0.0050	10	0	10	9	9	0	9	8
7373.6704	7373.6755	-0.0051	10	2	9	10	9	2	8	9
7373.6704	7373.6794	-0.0090	10	2	9	11	9	2	8	10
7373.7006	7373.7081	-0.0075	10	2	9	9	9	2	8	8
7382.9148	7382.9106	0.0042	10	9	1	11	9	9	0	10
7383.1113	7383.1105	0.0008	10	8	2	11	9	8	1	10
7383.1113	7383.1105	0.0008	10	8	3	11	9	8	2	10
7383.2723	7383.2690	0.0033	10	8	3	10	9	8	2	9
7383.2723	7383.2690	0.0033	10	8	2	10	9	8	1	9
7383.3873	7383.3783	0.0090	10	7	4	9	9	7	3	8
7383.3873	7383.3933	-0.0060	10	7	3	11	9	7	2	10
7383.3873	7383.3933	-0.0060	10	7	4	11	9	7	3	10
7383.3873	7383.3783	0.0090	10	7	3	9	9	7	2	8
7383.5147	7383.5168	-0.0021	10	7	4	10	9	7	3	9
7383.5147	7383.5168	-0.0021	10	7	3	10	9	7	2	9
7383.8162	7383.8131	0.0031	10	6	5	9	9	6	4	8
7383.8162	7383.8231	-0.0069	10	6	5	11	9	6	4	10
7383.8162	7383.8131	0.0031	10	6	4	9	9	6	3	8
7383.8162	7383.8231	-0.0069	10	6	4	11	9	6	3	10
7383.9129	7383.9169	-0.0040	10	6	4	10	9	6	3	9
7383.9129	7383.9169	-0.0040	10	6	5	10	9	6	4	9
7384.5330	7384.5391	-0.0061	10	5	5	11	9	5	4	10
7384.5330	7384.5383	-0.0053	10	5	6	11	9	5	5	10
7384.5330	7384.5333	-0.0003	10	5	5	9	9	5	4	8
7384.5330	7384.5325	0.0005	10	5	6	9	9	5	5	8
7384.6111	7384.6091	0.0020	10	5	5	10	9	5	4	9

7384.6111	7384.6084	0.0027	10	5	6	10	9	5	5	9
7387.5254	7387.5173	0.0081	10	3	8	9	9	3	7	8
7387.5254	7387.5168	0.0086	10	3	8	11	9	3	7	10
7390.6434	7390.6354	0.0080	10	3	7	9	9	3	6	8
7390.6434	7390.6367	0.0067	10	3	7	11	9	3	6	10
7422.2798	7422.2797	0.0001	10	2	8	11	9	2	7	10
7422.2798	7422.2800	-0.0002	10	2	8	9	9	2	7	8
7422.4120	7422.4117	0.0003	10	2	8	10	9	2	7	9
7448.3535	7448.3572	-0.0037	10	1	9	11	9	1	8	10
7448.3872	7448.3804	0.0068	10	1	9	9	9	1	8	8
7479.9074	7479.9128	-0.0054	6	3	4	6	5	2	3	5
7480.1887	7480.1827	0.0060	6	3	4	7	5	2	3	6
7480.2362	7480.2446	-0.0084	6	3	4	5	5	2	3	4
7491.6696	7491.6690	0.0006	6	3	3	6	5	2	4	5
7491.6696	7491.6739	-0.0043	6	3	3	7	5	2	4	6
7491.6826	7491.6863	-0.0037	6	3	3	5	5	2	4	4
7512.7246	7512.7194	0.0052	8	2	7	8	7	1	6	7
7514.1583	7514.1534	0.0049	8	2	7	9	7	1	6	8
7514.3678	7514.3640	0.0038	8	2	7	7	7	1	6	6
7601.2954	7601.3033	-0.0079	12	1	11	11	11	2	10	10
7601.4130	7601.4064	0.0066	12	1	11	13	11	2	10	12
7615.0117	7615.0086	0.0031	10	1	10	10	9	0	9	9
7615.8866	7615.8958	-0.0092	10	1	10	11	9	0	9	10
7615.9799	7615.9807	-0.0008	10	1	10	9	9	0	9	8
7774.4787	7774.4854	-0.0067	11	0	11	10	10	1	10	9
7774.5475	7774.5509	-0.0034	11	0	11	12	10	1	10	11
7775.1390	7775.1376	0.0014	11	0	11	11	10	1	10	10

E Isomer

Table S9: Observed and calculated rotational transitions (MHz) for the E Isomer

Observed	Calculated	Obs-Calc	J'	K_a'	K_c'	F'	J"	K_a"	K_c"	F"
2011.3274	2011.3275	-0.0001	2	1	2	2	1	0	1	1
2012.6752	2012.6746	0.0006	2	1	2	3	1	0	1	2
2012.9196	2012.9197	-0.0001	2	1	2	1	1	0	1	1
2013.7309	2013.7316	-0.0007	2	1	2	1	1	0	1	0
2023.4312	2023.4354	-0.0042	7	2	6	7	7	1	7	7
2190.3942	2190.3855	0.0087	15	3	12	16	15	2	13	16
2247.4918	2247.4973	-0.0055	5	1	4	6	4	2	3	5
2248.8986	2248.9031	-0.0045	5	1	4	5	4	2	3	4
2252.7740	2252.7767	-0.0027	14	3	11	15	14	2	12	15
2324.9940	2324.9868	0.0072	13	3	10	13	13	2	11	13
2325.6401	2325.6353	0.0048	13	3	10	14	13	2	11	14
2372.0662	2372.0747	-0.0085	10	2	9	9	10	1	10	9
2403.3819	2403.3912	-0.0093	12	3	9	13	12	2	10	13
2403.4424	2403.4523	-0.0099	12	3	9	11	12	2	10	11
2480.7030	2480.7126	-0.0096	11	3	8	12	11	2	9	12
2480.7718	2480.7813	-0.0095	11	3	8	10	11	2	9	10
2493.8064	2493.8132	-0.0068	4	0	4	3	3	1	3	2
2495.2995	2495.2919	0.0076	4	0	4	4	3	1	3	3
2528.5179	2528.5148	0.0031	7	2	5	8	6	3	4	7
2552.1762	2552.1701	0.0061	10	3	7	10	10	2	8	10
2553.0022	2553.0028	-0.0006	10	3	7	9	10	2	8	9
2615.6799	2615.6780	0.0019	9	3	6	9	9	2	7	9
2616.4760	2616.4828	-0.0068	9	3	6	8	9	2	7	8

2668.1622	2668.1677	-0.0055	8	3	5	8	8	2	6	8
2668.8358	2668.8334	0.0024	8	3	5	9	8	2	6	9
2668.9163	2668.9176	-0.0013	8	3	5	7	8	2	6	7
2669.7989	2669.8042	-0.0053	12	2	11	11	12	1	12	11
2708.7641	2708.7626	0.0015	7	3	4	7	7	2	5	7
2709.3576	2709.3564	0.0012	7	3	4	8	7	2	5	8
2709.4473	2709.4423	0.0050	7	3	4	6	7	2	5	6
2721.5351	2721.5341	0.0010	3	1	3	3	2	0	2	2
2722.8185	2722.8153	0.0032	3	1	3	4	2	0	2	3
2723.3293	2723.3256	0.0037	3	1	3	2	2	0	2	1
2737.9397	2737.9302	0.0095	6	3	3	6	6	2	4	6
2738.4555	2738.4533	0.0022	6	3	3	7	6	2	4	7
2738.5478	2738.5419	0.0059	6	3	3	5	6	2	4	5
2785.5758	2785.5686	0.0072	5	3	3	5	5	2	4	5
2785.6610	2785.6567	0.0043	5	3	3	6	5	2	4	6
2805.6324	2805.6307	0.0017	7	3	5	6	7	2	6	6
2805.7480	2805.7440	0.0040	7	3	5	7	7	2	6	7
2823.7235	2823.7255	-0.0020	8	3	6	8	8	2	7	8
2881.4197	2881.4192	0.0005	10	3	8	11	10	2	9	11
2881.7407	2881.7462	-0.0055	10	3	8	10	10	2	9	10
2923.8057	2923.8161	-0.0104	11	3	9	12	11	2	10	12
2924.2093	2924.2139	-0.0046	11	3	9	11	11	2	10	11
3063.2091	3063.2148	-0.0057	6	1	5	5	5	2	4	4
3063.4304	3063.4320	-0.0016	6	1	5	7	5	2	4	6
3064.7567	3064.7611	-0.0044	6	1	5	6	5	2	4	5
3141.6474	3141.6544	-0.0070	7	1	7	7	6	2	4	6
3143.5238	3143.5297	-0.0059	7	1	7	8	6	2	4	7

3147.4023	3147.4002	0.0021	2	2	1	2	1	1	0	1
3148.7268	3148.7239	0.0029	2	2	1	3	1	1	0	2
3155.9423	3155.9473	-0.0050	8	2	7	8	7	3	4	7
3156.1015	3156.1062	-0.0047	8	2	7	9	7	3	4	8
3173.6397	3173.6412	-0.0015	2	2	0	2	1	1	1	2
3173.9402	3173.9353	0.0049	2	2	0	3	1	1	1	2
3174.9272	3174.9206	0.0066	2	2	0	2	1	1	1	1
3175.3726	3175.3798	-0.0072	2	2	0	1	1	1	1	1
3275.7260	3275.7289	-0.0029	5	0	5	4	4	1	4	3
3275.9835	3275.9817	0.0018	5	0	5	6	4	1	4	5
3276.9573	3276.9603	-0.0030	5	0	5	5	4	1	4	4
3322.1773	3322.1753	0.0020	8	2	6	7	7	3	5	6
3322.2754	3322.2705	0.0049	8	2	6	9	7	3	5	8
3323.0673	3323.0671	0.0002	8	2	6	8	7	3	5	7
3420.7032	3420.7034	-0.0002	4	1	4	4	3	0	3	3
3421.9332	3421.9361	-0.0029	4	1	4	5	3	0	3	4
3422.2296	3422.2372	-0.0076	4	1	4	3	3	0	3	2
3799.9069	3799.9123	-0.0054	12	4	8	11	12	3	9	11
3864.6780	3864.6873	-0.0093	9	4	5	9	9	3	6	9
3869.6046	3869.6027	0.0019	3	2	2	3	2	1	1	2
3870.9785	3870.9736	0.0049	3	2	2	4	2	1	1	3
3871.7366	3871.7359	0.0007	3	2	2	2	2	1	1	1
3876.1371	3876.1367	0.0004	9	2	8	10	8	3	5	9
3877.1953	3877.1922	0.0031	12	4	9	12	12	3	10	12
3877.2347	3877.2403	-0.0056	12	4	9	11	12	3	10	11
3877.2347	3877.2385	-0.0038	12	4	9	13	12	3	10	13
3878.0440	3878.0469	-0.0029	10	4	7	10	10	3	8	10

3878.1468	3878.1531	-0.0063	10	4	7	9	10	3	8	9
3878.1468	3878.1433	0.0035	10	4	7	11	10	3	8	11
3880.0245	3880.0294	-0.0049	9	4	6	9	9	3	7	9
3880.1422	3880.1496	-0.0074	9	4	6	10	9	3	7	10
3880.7360	3880.7319	0.0041	7	4	3	7	7	3	4	7
3884.3161	3884.3124	0.0037	7	4	4	7	7	3	5	7
3884.7949	3884.7993	-0.0044	6	4	2	7	6	3	3	7
3884.8396	3884.8401	-0.0005	6	4	2	5	6	3	3	5
3888.5861	3888.5907	-0.0046	7	1	6	6	6	2	5	5
3888.7627	3888.7686	-0.0059	7	1	6	8	6	2	5	7
3890.0307	3890.0379	-0.0072	7	1	6	7	6	2	5	6
3948.1904	3948.1884	0.0020	3	2	1	2	2	1	2	1
3948.8092	3948.8057	0.0035	3	2	1	4	2	1	2	3
3949.9737	3949.9672	0.0065	3	2	1	3	2	1	2	2
4060.1132	4060.1120	0.0012	6	0	6	5	5	1	5	4
4060.2981	4060.2911	0.0070	6	0	6	7	5	1	5	6
4061.1422	4061.1398	0.0024	6	0	6	6	5	1	5	5
4110.6028	4110.5948	0.0080	5	1	5	5	4	0	4	4
4111.7432	4111.7381	0.0051	5	1	5	6	4	0	4	5
4111.9730	4111.9767	-0.0037	5	1	5	4	4	0	4	3
4131.5057	4131.5094	-0.0037	9	2	7	8	8	3	6	7
4131.5981	4131.6020	-0.0039	9	2	7	10	8	3	6	9
4132.4821	4132.4843	-0.0022	9	2	7	9	8	3	6	8
4402.1213	4402.1234	-0.0021	11	3	8	12	10	4	7	11
4402.4874	4402.4841	0.0033	11	3	8	11	10	4	7	10
4579.3252	4579.3207	0.0045	4	2	3	4	3	1	2	3
4580.6832	4580.6803	0.0029	4	2	3	5	3	1	2	4

4581.1701	4581.1691	0.0010	4	2	3	3	3	1	2	2
4722.2411	4722.2421	-0.0010	8	1	7	7	7	2	6	6
4722.3914	4722.3911	0.0003	8	1	7	9	7	2	6	8
4723.6073	4723.6046	0.0027	8	1	7	8	7	2	6	7
4740.4105	4740.4117	-0.0012	4	2	2	3	3	1	3	2
4740.8562	4740.8572	-0.0010	4	2	2	5	3	1	3	4
4742.2370	4742.2324	0.0046	4	2	2	4	3	1	3	3
4793.4462	4793.4499	-0.0037	6	1	6	6	5	0	5	5
4794.4958	4794.4962	-0.0004	6	1	6	7	5	0	5	6
4794.6740	4794.6712	0.0028	6	1	6	5	5	0	5	4
4843.9783	4843.9779	0.0004	7	0	7	6	6	1	6	5
4844.1078	4844.1063	0.0015	7	0	7	8	6	1	6	7
4844.8182	4844.8201	-0.0019	7	0	7	7	6	1	6	6
4958.3451	4958.3408	0.0043	10	2	8	10	9	3	7	9
4967.7759	4967.7751	0.0008	13	5	8	12	13	4	9	12
4989.2717	4989.2748	-0.0031	10	5	5	11	10	4	6	11
4990.0121	4990.0118	0.0003	10	5	6	10	10	4	7	10
4990.1644	4990.1624	0.0020	10	5	6	9	10	4	7	9
4998.4220	4998.4297	-0.0077	6	5	1	6	6	4	2	6
4998.4416	4998.4433	-0.0017	6	5	2	6	6	4	3	6
4998.7107	4998.7118	-0.0011	6	5	2	7	6	4	3	7
5018.4415	5018.4450	-0.0035	3	3	1	3	2	2	0	3
5018.7378	5018.7391	-0.0013	3	3	1	3	2	2	0	2
5018.8832	5018.8793	0.0039	3	3	1	4	2	2	0	3
5019.2910	5019.2902	0.0008	3	3	0	3	2	2	1	3
5019.6688	5019.6602	0.0086	3	3	0	3	2	2	1	2
5019.6729	5019.6691	0.0038	3	3	0	2	2	2	1	1

5019.7160	5019.7237	-0.0077	3	3	0	4	2	2	1	3
5100.6886	5100.6887	-0.0001	12	3	10	11	11	4	7	10
5100.6886	5100.6902	-0.0016	12	3	10	13	11	4	7	12
5100.7711	5100.7686	0.0025	12	3	10	12	11	4	7	11
5183.6750	5183.6786	-0.0036	12	3	9	11	11	4	8	10
5184.1351	5184.1263	0.0088	12	3	9	12	11	4	8	11
5276.6355	5276.6353	0.0002	5	2	4	5	4	1	3	4
5277.6233	5277.6241	-0.0008	11	2	10	11	10	3	7	10
5277.9792	5277.9814	-0.0022	5	2	4	6	4	1	3	5
5278.1694	5278.1631	0.0063	11	2	10	12	10	3	7	11
5278.2097	5278.2183	-0.0086	11	2	10	10	10	3	7	9
5278.3362	5278.3381	-0.0019	5	2	4	4	4	1	3	3
5471.9861	5471.9822	0.0039	7	1	7	7	6	0	6	6
5472.9154	5472.9130	0.0024	7	1	7	8	6	0	6	7
5473.0433	5473.0435	-0.0002	7	1	7	6	6	0	6	5
5552.8743	5552.8709	0.0034	5	2	3	4	4	1	4	3
5553.2514	5553.2469	0.0045	5	2	3	6	4	1	4	5
5554.8328	5554.8305	0.0023	5	2	3	5	4	1	4	4
5562.7381	5562.7455	-0.0074	9	1	8	8	8	2	7	7
5562.8692	5562.8717	-0.0025	9	1	8	10	8	2	7	9
5564.0228	5564.0262	-0.0034	9	1	8	9	8	2	7	8
5624.5773	5624.5753	0.0020	8	0	8	7	7	1	7	6
5624.6679	5624.6690	-0.0011	8	0	8	9	7	1	7	8
5625.2489	5625.2471	0.0018	8	0	8	8	7	1	7	7
5768.2604	5768.2535	0.0069	4	3	1	4	3	2	2	3
5768.3399	5768.3357	0.0042	4	3	1	5	3	2	2	4
5799.4731	5799.4651	0.0080	11	2	9	10	10	3	8	9

5799.5486	5799.5541	-0.0055	11	2	9	12	10	3	8	11
5800.5778	5800.5751	0.0027	11	2	9	11	10	3	8	10
5846.8715	5846.8759	-0.0044	13	3	11	13	12	4	8	12
5952.5895	5952.5903	-0.0008	12	2	11	12	11	3	8	11
5953.2750	5953.2775	-0.0025	12	2	11	13	11	3	8	12
5953.3420	5953.3409	0.0011	12	2	11	11	11	3	8	10
5961.7777	5961.7793	-0.0016	6	2	5	6	5	1	4	5
5963.1103	5963.1111	-0.0008	6	2	5	7	5	1	4	6
5977.3580	5977.3536	0.0044	13	3	10	14	12	4	9	13
6108.8124	6108.8184	-0.0060	8	6	3	8	8	5	4	8
6108.8124	6108.8171	-0.0047	8	6	2	8	8	5	3	8
6109.0492	6109.0405	0.0087	8	6	3	7	8	5	4	7
6109.0492	6109.0392	0.0100	8	6	2	7	8	5	3	7
6109.7029	6109.7083	-0.0054	7	6	1	7	7	5	2	7
6109.7029	6109.7086	-0.0057	7	6	2	7	7	5	3	7
6110.2700	6110.2746	-0.0046	6	6	0	6	6	5	1	6
6110.2700	6110.2747	-0.0047	6	6	1	6	6	5	2	6
6110.5909	6110.5922	-0.0013	6	6	1	7	6	5	2	7
6110.5909	6110.5922	-0.0013	6	6	0	7	6	5	1	7
6149.0588	6149.0551	0.0037	8	1	8	8	7	0	7	7
6149.9652	6149.9557	0.0095	8	1	8	7	7	0	7	6
6389.3378	6389.3358	0.0020	6	2	4	5	5	1	5	4
6389.6764	6389.6744	0.0020	6	2	4	7	5	1	5	6
6391.4742	6391.4676	0.0066	6	2	4	6	5	1	5	5
6399.7396	6399.7425	-0.0029	9	0	9	8	8	1	8	7
6399.8118	6399.8096	0.0022	9	0	9	10	8	1	8	9
6400.2624	6400.2610	0.0014	9	0	9	9	8	1	8	8

6408.4311	6408.4244	0.0067	10	1	9	11	9	2	8	10
6409.4223	6409.4265	-0.0042	10	1	9	10	9	2	8	9
6505.8812	6505.8727	0.0085	5	3	3	5	4	2	2	4
6518.9245	6518.9253	-0.0008	5	3	2	4	4	2	3	3
6588.4524	6588.4510	0.0014	14	3	12	15	13	4	9	14
6604.9709	6604.9674	0.0035	13	2	12	13	12	3	9	12
6605.8172	6605.8169	0.0003	13	2	12	14	12	3	9	13
6605.8934	6605.8868	0.0066	13	2	12	12	12	3	9	11
6635.1488	6635.1463	0.0025	7	2	6	7	6	1	5	6
6636.4630	6636.4615	0.0015	7	2	6	8	6	1	5	7
6636.6881	6636.6877	0.0004	7	2	6	6	6	1	5	5
6657.1618	6657.1604	0.0014	12	2	10	11	11	3	9	10
6657.2464	6657.2456	0.0008	12	2	10	13	11	3	9	12
6658.3085	6658.3103	-0.0018	12	2	10	12	11	3	9	11
6786.1102	6786.1051	0.0051	14	3	11	14	13	4	10	13
6827.3556	6827.3598	-0.0042	9	1	9	9	8	0	8	8
6828.0362	6828.0366	-0.0004	9	1	9	10	8	0	8	9
6828.1013	6828.1049	-0.0036	9	1	9	8	8	0	8	7
6877.0788	6877.0894	-0.0106	4	4	0	4	3	3	1	4
6877.0788	6877.0720	0.0068	4	4	1	4	3	3	0	4
6877.5773	6877.5705	0.0068	4	4	0	3	3	3	1	2
6877.5773	6877.5758	0.0015	4	4	1	5	3	3	0	4
7168.1247	7168.1166	0.0081	10	0	10	9	9	1	9	8
7168.1604	7168.1645	-0.0041	10	0	10	11	9	1	9	10
7168.5002	7168.5033	-0.0031	10	0	10	10	9	1	9	9
7216.0085	7215.9950	0.0135	12	7	5	11	12	6	6	11
7216.0085	7215.9963	0.0122	12	7	6	11	12	6	7	11

7220.9125	7220.9078	0.0047	8	7	1	8	8	6	2	8
7220.9125	7220.9078	0.0047	8	7	2	8	8	6	3	8
7230.4027	7230.4065	-0.0038	14	2	13	14	13	3	10	13
7231.4243	7231.4286	-0.0043	14	2	13	15	13	3	10	14
7231.5097	7231.5069	0.0028	14	2	13	13	13	3	10	12
7242.3902	7242.3958	-0.0056	6	3	4	6	5	2	3	5
7242.7528	7242.7599	-0.0071	6	3	4	7	5	2	3	6
7242.8374	7242.8418	-0.0044	6	3	4	5	5	2	3	4
7253.7289	7253.7291	-0.0002	7	2	5	6	6	1	6	5
7254.0432	7254.0437	-0.0005	7	2	5	8	6	1	6	7
7256.0495	7256.0480	0.0015	7	2	5	7	6	1	6	6
7256.7918	7256.7901	0.0017	11	1	10	10	10	2	9	9
7256.8847	7256.8818	0.0029	11	1	10	12	10	2	9	11
7257.8926	7257.8938	-0.0012	11	1	10	11	10	2	9	10
7272.7565	7272.7479	0.0086	6	3	3	6	5	2	4	5
7297.3378	7297.3415	-0.0037	8	2	7	8	7	1	6	7
7298.6282	7298.6336	-0.0054	8	2	7	9	7	1	6	8
7298.8170	7298.8230	-0.0060	8	2	7	7	7	1	6	6
7509.0721	7509.0729	-0.0008	10	1	10	10	9	0	9	9
7528.8666	7528.8743	-0.0077	13	2	11	12	12	3	10	11
7610.5336	7610.5333	0.0003	15	3	12	16	14	4	11	15
7611.1827	7611.1825	0.0002	15	3	12	15	14	4	11	14
7929.1805	7929.1740	0.0065	11	0	11	10	10	1	10	9
7929.2053	7929.2080	-0.0027	11	0	11	12	10	1	10	11
7929.4492	7929.4522	-0.0030	11	0	11	11	10	1	10	10
7949.3246	7949.3188	0.0058	9	2	8	9	8	1	7	8
7950.4894	7950.4944	-0.0050	9	2	8	10	8	1	7	9

7950.8124	7950.8044	0.0080	9	2	8	8	8	1	7	7
7970.8279	7970.8338	-0.0059	7	3	5	7	6	2	4	6
7971.2890	7971.2963	-0.0073	7	3	5	8	6	2	4	7
7971.3763	7971.3819	-0.0056	7	3	5	6	6	2	4	5

1-w-Z_N Cluster

Table S10: Observed and calculated rotational transitions (MHz) for 1-w-Z_N

Observed	Calculated	Obs-Calc	J'	K_a'	K_c'	F'	J"	K_a"	K_c"	F"
2024.5915	2024.5793	0.0122	3	2	1	3	2	2	0	2
2079.5251	2079.5101	0.0150	3	1	2	4	2	1	1	3
2079.7527	2079.7551	-0.0024	3	1	2	2	2	1	1	1
2184.7912	2184.7778	0.0134	8	4	4	9	8	3	5	9
2237.4939	2237.4854	0.0085	10	6	4	11	9	7	3	10
2384.3513	2384.3570	-0.0057	4	0	4	5	3	1	3	4
2384.7911	2384.8009	-0.0098	4	0	4	4	3	1	3	3
2514.9647	2514.9651	-0.0004	4	1	4	4	3	1	3	3
2514.9647	2514.9727	-0.0080	4	1	4	3	3	1	3	2
2515.0516	2515.0502	0.0014	4	1	4	5	3	1	3	4
2578.2780	2578.2758	0.0022	4	0	4	4	3	0	3	3
2578.6099	2578.6137	-0.0038	4	0	4	5	3	0	3	4
2578.6841	2578.6742	0.0099	4	0	4	3	3	0	3	2
2674.1762	2674.1831	-0.0069	4	3	1	3	3	3	0	2
2674.2759	2674.2807	-0.0048	4	3	1	5	3	3	0	4
2674.6045	2674.6062	-0.0017	4	3	1	4	3	3	0	3
2724.9422	2724.9486	-0.0064	4	2	2	3	3	2	1	2

2761.0141	2761.0141	-0.0000	4	1	3	4	3	1	2	3
2761.0919	2761.0898	0.0021	4	1	3	5	3	1	2	4
2761.2286	2761.2276	0.0010	4	1	3	3	3	1	2	2
3051.8976	3051.9052	-0.0076	5	0	5	4	4	1	4	3
3052.0049	3052.0111	-0.0062	5	0	5	6	4	1	4	5
3052.2240	3052.2291	-0.0051	5	0	5	5	4	1	4	4
3132.1391	3132.1418	-0.0027	5	1	5	5	4	1	4	4
3132.2277	3132.2348	-0.0071	5	1	5	6	4	1	4	5
3182.3940	3182.3932	0.0008	5	0	5	5	4	0	4	4
3182.6984	3182.7044	-0.0060	5	0	5	6	4	0	4	5
3182.7291	3182.7345	-0.0054	5	0	5	4	4	0	4	3
3300.1773	3300.1816	-0.0043	5	2	4	6	4	2	3	5
3355.6444	3355.6522	-0.0078	5	3	2	4	4	3	1	3
3355.7000	3355.7029	-0.0029	5	3	2	6	4	3	1	5
3355.9550	3355.9570	-0.0020	5	3	2	5	4	3	1	4
3430.4440	3430.4383	0.0057	5	1	4	5	4	1	3	4
3430.5972	3430.5944	0.0028	5	1	4	6	4	1	3	5
3430.6916	3430.6893	0.0023	5	1	4	4	4	1	3	3
3435.9976	3435.9965	0.0011	5	2	3	6	4	2	2	5
3668.9641	3668.9601	0.0040	16	6	11	15	16	5	12	15
3668.9641	3668.9614	0.0027	16	6	11	17	16	5	12	17
3698.6538	3698.6503	0.0035	6	0	6	5	5	1	5	4
3698.7187	3698.7161	0.0026	6	0	6	7	5	1	5	6
3698.7847	3698.7814	0.0033	6	0	6	6	5	1	5	5
3724.6282	3724.6239	0.0043	3	3	0	3	2	2	1	2
3744.4449	3744.4395	0.0054	6	1	6	6	5	1	5	5
3778.6970	3778.6941	0.0029	6	0	6	6	5	0	5	5

3778.9442	3778.9397	0.0045	6	0	6	7	5	0	5	6
3778.9442	3778.9486	-0.0044	6	0	6	5	5	0	5	4
3945.0960	3945.0988	-0.0028	6	2	5	6	5	2	4	5
3945.1748	3945.1737	0.0011	6	2	5	7	5	2	4	6
3945.1748	3945.1862	-0.0114	6	2	5	5	5	2	4	4
4010.8317	4010.8409	-0.0092	6	4	3	7	5	4	2	6
4011.8145	4011.8124	0.0021	6	3	4	5	5	3	3	4
4011.8145	4011.8196	-0.0051	6	3	4	7	5	3	3	6
4011.9040	4011.9075	-0.0035	6	3	4	6	5	3	3	5
4012.8116	4012.8166	-0.0050	6	4	2	6	5	4	1	5
4048.7167	4048.7179	-0.0012	6	3	3	7	5	3	2	6
4082.8941	4082.8894	0.0047	6	1	5	6	5	1	4	5
4083.1225	4083.1193	0.0032	6	1	5	7	5	1	4	6
4083.1969	4083.1942	0.0027	6	1	5	5	5	1	4	4
4147.1607	4147.1633	-0.0026	6	2	4	5	5	2	3	4
4328.0751	4328.0669	0.0082	7	0	7	7	6	1	6	6
4328.0751	4328.0747	0.0004	7	0	7	8	6	1	6	7
4352.8210	4352.8257	-0.0047	7	1	7	7	6	1	6	6
4352.9315	4352.9212	0.0103	7	1	7	8	6	1	6	7
4373.7248	4373.7250	-0.0002	7	0	7	7	6	0	6	6
4373.9013	4373.9021	-0.0008	7	0	7	6	6	0	6	5
4373.9013	4373.9052	-0.0039	7	0	7	8	6	0	6	7
4582.6572	4582.6561	0.0011	7	2	6	7	6	2	5	6
4582.7620	4582.7565	0.0055	7	2	6	8	6	2	5	7
4582.7620	4582.7701	-0.0081	7	2	6	6	6	2	5	5
4602.0674	4602.0675	-0.0001	16	1	15	16	16	0	16	16
4670.2746	4670.2638	0.0108	7	6	1	6	6	6	0	5

4670.2746	4670.2615	0.0131	7	6	2	6	6	6	1	5
4680.0517	4680.0520	-0.0003	7	3	5	6	6	3	4	5
4680.0517	4680.0502	0.0015	7	3	5	8	6	3	4	7
4680.0797	4680.0895	-0.0098	7	3	5	7	6	3	4	6
4686.3420	4686.3482	-0.0062	7	4	4	8	6	4	3	7
4686.3420	4686.3339	0.0081	7	4	4	6	6	4	3	5
4686.4696	4686.4749	-0.0053	7	4	4	7	6	4	3	6
4692.1875	4692.1792	0.0083	7	4	3	8	6	4	2	7
4692.3258	4692.3348	-0.0090	7	4	3	7	6	4	2	6
4714.4124	4714.4119	0.0005	7	1	6	7	6	1	5	6
4714.6996	4714.7012	-0.0016	7	1	6	8	6	1	5	7
4714.7669	4714.7627	0.0042	7	1	6	6	6	1	5	5
4756.4494	4756.4429	0.0065	7	3	4	7	6	3	3	6
4849.5129	4849.5068	0.0061	7	2	5	8	6	2	4	7
4945.4328	4945.4295	0.0033	8	0	8	8	7	1	7	7
4958.3558	4958.3583	-0.0025	8	1	8	8	7	1	7	7
4958.4426	4958.4445	-0.0019	8	1	8	9	7	1	7	8
4958.4426	4958.4288	0.0138	8	1	8	7	7	1	7	6
4970.1874	4970.1883	-0.0009	8	0	8	8	7	0	7	7
4970.3152	4970.3195	-0.0043	8	0	8	9	7	0	7	8
4970.3152	4970.3111	0.0041	8	0	8	7	7	0	7	6
4983.4322	4983.4272	0.0050	8	1	7	7	7	2	6	6
4983.4711	4983.4772	-0.0061	8	1	7	9	7	2	6	8
4983.8560	4983.8465	0.0095	8	1	7	8	7	2	6	7
5075.4484	5075.4327	0.0157	4	4	0	5	3	3	1	4
5212.5724	5212.5762	-0.0038	8	2	7	8	7	2	6	7
5212.6891	5212.6933	-0.0042	8	2	7	9	7	2	6	8

5325.0769	5325.0734	0.0035	8	1	7	8	7	1	6	7
5325.3930	5325.3882	0.0048	8	1	7	9	7	1	6	8
5343.7725	5343.7767	-0.0042	8	3	6	7	7	3	5	6
5343.7725	5343.7706	0.0019	8	3	6	8	7	3	5	7
5343.7725	5343.7709	0.0016	8	3	6	9	7	3	5	8
5363.5043	5363.5072	-0.0029	8	4	5	7	7	4	4	6
5378.9429	5378.9381	0.0048	8	4	4	7	7	4	3	6
5379.0999	5379.1042	-0.0043	8	4	4	8	7	4	3	7
5476.8221	5476.8197	0.0024	8	3	5	9	7	3	4	8
5555.4565	5555.4474	0.0091	9	0	9	9	8	1	8	8
5555.5165	5555.5030	0.0135	9	0	9	10	8	1	8	9
5562.0702	5562.0756	-0.0054	9	1	9	8	8	1	8	7
5568.3777	5568.3762	0.0015	9	0	9	9	8	0	8	8
5743.4403	5743.4380	0.0023	5	4	1	4	4	3	2	3
5765.4136	5765.4185	-0.0049	16	9	8	17	16	8	9	17
5835.2125	5835.2161	-0.0036	9	2	8	9	8	2	7	8
5835.3396	5835.3410	-0.0014	9	2	8	10	8	2	7	9
5920.9955	5920.9935	0.0020	9	1	8	10	8	1	7	9
6076.0437	6076.0452	-0.0015	9	4	5	10	8	4	4	9
6076.0437	6076.0333	0.0104	9	4	5	8	8	4	4	7
6076.2114	6076.2130	-0.0016	9	4	5	9	8	4	4	8
6167.8948	6167.8921	0.0027	10	0	10	9	9	0	9	8
6203.3179	6203.3081	0.0098	9	3	6	10	8	3	5	9
6205.8020	6205.7966	0.0054	9	2	7	9	8	2	6	8
6205.9313	6205.9194	0.0119	9	2	7	10	8	2	6	9
6205.9517	6205.9520	-0.0003	9	2	7	8	8	2	6	7
6414.8956	6414.8989	-0.0033	6	4	2	7	5	3	3	6

6451.4603	6451.4713	-0.0110	10	2	9	10	9	2	8	9
6509.9324	6509.9422	-0.0098	10	1	9	10	9	1	8	9
6510.2091	6510.2203	-0.0112	10	1	9	9	9	1	8	8
6510.2091	6510.1959	0.0132	10	1	9	11	9	1	8	10
6623.0753	6623.0691	0.0062	15	4	12	15	14	5	9	14
6709.3371	6709.3432	-0.0061	10	5	6	9	9	5	5	8
6709.3371	6709.3485	-0.0114	10	5	6	11	9	5	5	10
6709.4264	6709.4374	-0.0110	10	5	6	10	9	5	5	9
6715.3110	6715.3116	-0.0006	10	5	5	9	9	5	4	8
6715.3110	6715.3187	-0.0077	10	5	5	11	9	5	4	10
6715.4265	6715.4267	-0.0002	10	5	5	10	9	5	4	9
6717.4115	6717.4079	0.0036	10	4	7	9	9	4	6	8
6717.4115	6717.4083	0.0032	10	4	7	11	9	4	6	10
6786.6272	6786.6211	0.0061	10	4	6	11	9	4	5	10
6786.8174	6786.8128	0.0046	10	4	6	10	9	4	5	9
6851.8587	6851.8579	0.0008	10	2	8	10	9	2	7	9
6852.0507	6852.0514	-0.0007	10	2	8	11	9	2	7	10
6852.0767	6852.0846	-0.0079	10	2	8	9	9	2	7	8
7014.5782	7014.5856	-0.0074	11	1	10	11	10	2	9	10
7062.5842	7062.5868	-0.0026	11	2	10	11	10	2	9	10
7062.7042	7062.7086	-0.0044	11	2	10	12	10	2	9	11
7062.7042	7062.7147	-0.0105	11	2	10	10	10	2	9	9
7095.2563	7095.2586	-0.0023	7	4	3	8	6	3	4	7
7095.2563	7095.2621	-0.0058	7	4	3	6	6	3	4	5

1-w-Z_O Cluster

Table S11: Observed and calculated rotational transitions (MHz) for 1-w-Z_O

Observed	Calculated	Obs-Calc	J'	K_a'	K_c'	F'	J"	K_a"	K_c"	F"
2096.0676	2096.0644	0.0032	3	1	2	4	2	1	1	3
2096.1420	2096.1578	-0.0158	3	1	2	3	2	1	1	2
2494.8018	2494.8115	-0.0097	4	1	4	3	3	1	3	2
2494.8018	2494.8032	-0.0014	4	1	4	5	3	1	3	4
2543.3189	2543.3050	0.0139	4	0	4	5	3	0	3	4
2772.7690	2772.7860	-0.0170	4	2	2	5	3	2	1	4
2772.8756	2772.8688	0.0068	4	1	3	5	3	1	2	4
2772.8757	2772.8906	-0.0149	4	1	3	4	3	1	2	3
2772.9902	2772.9813	0.0089	4	2	2	4	3	2	1	3
3101.3802	3101.3849	-0.0047	5	1	5	4	4	1	4	3
3101.3802	3101.3760	0.0042	5	1	5	6	4	1	4	5
3101.3802	3101.3857	-0.0055	5	1	5	5	4	1	4	4
3131.3935	3131.4159	-0.0224	5	0	5	6	4	0	4	5
3131.3935	3131.3909	0.0026	5	0	5	5	4	0	4	4
3297.3613	3297.3634	-0.0021	5	2	4	6	4	2	3	5
3297.3613	3297.3575	0.0038	5	2	4	4	4	2	3	3
3297.4317	3297.4319	-0.0002	5	2	4	5	4	2	3	4
3400.1529	3400.1588	-0.0059	5	3	2	4	4	3	1	3
3400.1994	3400.2072	-0.0078	5	3	2	6	4	3	1	5
3400.4223	3400.4193	0.0030	5	3	2	5	4	3	1	4
3426.2687	3426.2994	-0.0307	5	1	4	4	4	1	3	3
3426.2687	3426.2750	-0.0063	5	1	4	6	4	1	3	5
3426.2687	3426.2623	0.0064	5	1	4	5	4	1	3	4
3494.2650	3494.2600	0.0050	5	2	3	4	4	2	2	3
3494.2650	3494.2647	0.0003	5	2	3	6	4	2	2	5

3494.3638	3494.3624	0.0014	5	2	3	5	4	2	2	4
3702.4675	3702.4679	-0.0004	6	1	6	6	5	1	5	5
3702.4675	3702.4660	0.0015	6	1	6	7	5	1	5	6
3702.4675	3702.4731	-0.0056	6	1	6	5	5	1	5	4
3718.3037	3718.3075	-0.0038	6	0	6	5	5	0	5	4
3718.3037	3718.2968	0.0069	6	0	6	7	5	0	5	6
3731.6468	3731.6575	-0.0107	6	1	6	7	5	0	5	6
3731.6468	3731.6326	0.0142	6	1	6	6	5	0	5	5
3731.6468	3731.6701	-0.0233	6	1	6	5	5	0	5	4
4035.0033	4034.9862	0.0171	6	3	4	5	5	3	3	4
4035.0033	4035.0015	0.0018	6	3	4	7	5	3	3	6
4035.1061	4035.1068	-0.0007	6	3	4	6	5	3	3	5
4043.5769	4043.5824	-0.0055	6	4	3	7	5	4	2	6
4043.7752	4043.7786	-0.0034	6	4	3	6	5	4	2	5
4049.6649	4049.6722	-0.0073	6	1	5	6	5	1	4	5
4050.6039	4050.5997	0.0042	6	4	2	6	5	4	1	6
4050.6039	4050.6139	-0.0100	6	4	2	7	5	4	1	6
4121.4098	4121.3995	0.0103	6	3	3	7	5	3	2	6
4121.5456	4121.5407	0.0049	6	3	3	6	5	3	2	5
4202.2065	4202.1877	0.0188	6	2	4	7	5	2	3	6
4202.2065	4202.1936	0.0129	6	2	4	5	5	2	3	4
4294.1792	4294.1683	0.0109	7	0	7	8	6	1	6	7
4294.1792	4294.1716	0.0076	7	0	7	7	6	1	6	6
4294.1792	4294.1731	0.0061	7	0	7	6	6	1	6	5
4299.9894	4299.9850	0.0044	7	1	7	7	6	1	6	6
4299.9894	4299.9859	0.0035	7	1	7	8	6	1	6	7
4299.9894	4299.9913	-0.0019	7	1	7	6	6	1	6	5

4307.5433	4307.5357	0.0076	7	0	7	6	6	0	6	5
4307.5433	4307.5290	0.0143	7	0	7	8	6	0	6	7
4554.8927	4554.8853	0.0074	7	2	6	8	6	2	5	7
4554.8927	4554.8897	0.0030	7	2	6	6	6	2	5	5
4554.8927	4554.8975	-0.0048	7	2	6	7	6	2	5	6
4645.6994	4645.7061	-0.0067	7	1	6	6	6	1	5	5
4645.6994	4645.6919	0.0075	7	1	6	8	6	1	5	7
4698.9381	4698.9353	0.0028	7	3	5	8	6	3	4	7
4698.9381	4698.9309	0.0072	7	3	5	6	6	3	4	5
4698.9867	4698.9964	-0.0097	7	3	5	7	6	3	4	6
4726.7818	4726.7848	-0.0030	7	4	4	6	6	4	3	5
4726.9264	4726.9295	-0.0031	7	4	4	7	6	4	3	6
4858.3098	4858.3062	0.0036	7	3	4	6	6	3	3	5
4858.3098	4858.3140	-0.0042	7	3	4	8	6	3	3	7
4858.3112	4858.3069	0.0043	8	2	6	8	7	3	5	7
4858.4183	4858.4114	0.0069	7	3	4	7	6	3	3	6
4887.1435	4887.1494	-0.0059	7	2	5	7	6	2	4	6
4887.1435	4887.1478	-0.0043	7	2	5	6	6	2	4	5
4887.1435	4887.1388	0.0047	7	2	5	8	6	2	4	7
4895.4760	4895.4722	0.0038	8	1	8	8	7	1	7	7
4895.4760	4895.4781	-0.0021	8	1	8	7	7	1	7	6
4895.4760	4895.4739	0.0021	8	1	8	9	7	1	7	8
4898.8487	4898.8418	0.0069	8	0	8	8	7	0	7	7
4898.8487	4898.8461	0.0026	8	0	8	9	7	0	7	8
4898.8487	4898.8507	-0.0020	8	0	8	7	7	0	7	6
5227.2162	5227.2285	-0.0123	8	1	7	8	7	1	6	7
5227.2590	5227.2582	0.0008	8	1	7	9	7	1	6	8

5227.2590	5227.2679	-0.0089	8	1	7	7	7	1	6	6
5403.7248	5403.7303	-0.0055	8	5	3	8	7	5	2	7
5409.3491	5409.3496	-0.0005	8	4	5	9	7	4	4	8
5409.3491	5409.3409	0.0082	8	4	5	7	7	4	4	6
5409.4351	5409.4338	0.0013	8	4	5	8	7	4	4	7
5489.9212	5489.9219	-0.0007	9	1	9	9	8	1	8	8
5489.9212	5489.9237	-0.0025	9	1	9	10	8	1	8	9
5489.9212	5489.9269	-0.0057	9	1	9	8	8	1	8	7
5542.1793	5542.1735	0.0058	8	2	6	7	7	2	5	6
5542.1793	5542.1639	0.0154	8	2	6	9	7	2	5	8
5595.0723	5595.0792	-0.0069	8	3	5	7	7	3	4	6
5595.0723	5595.0805	-0.0082	8	3	5	9	7	3	4	8
5595.1488	5595.1417	0.0071	8	3	5	8	7	3	4	7
5773.9788	5773.9743	0.0045	9	2	8	9	8	2	7	8
5773.9788	5773.9768	0.0020	9	2	8	10	8	2	7	9
5773.9788	5773.9809	-0.0021	9	2	8	8	8	2	7	7
5807.3823	5807.3629	0.0194	9	1	8	9	8	1	7	8
5807.3823	5807.3899	-0.0076	9	1	8	8	8	1	7	7
5807.3823	5807.3834	-0.0011	9	1	8	10	8	1	7	9
5993.9751	5993.9734	0.0017	9	3	7	8	8	3	6	7
5993.9751	5993.9888	-0.0137	9	3	7	9	8	3	6	8
5993.9751	5993.9715	0.0036	9	3	7	10	8	3	6	9
6083.4817	6083.4837	-0.0020	10	0	10	11	9	1	9	10
6083.4817	6083.4823	-0.0006	10	0	10	10	9	1	9	9
6083.4817	6083.4862	-0.0045	10	0	10	9	9	1	9	8
6083.8898	6083.8838	0.0060	10	1	10	10	9	1	9	9
6083.8898	6083.8879	0.0019	10	1	10	9	9	1	9	8

6083.8898	6083.8854	0.0044	10	1	10	11	9	1	9	10
6084.4865	6084.4830	0.0035	10	0	10	10	9	0	9	9
6084.4865	6084.4875	-0.0010	10	0	10	9	9	0	9	8
6084.4865	6084.4850	0.0015	10	0	10	11	9	0	9	10
6087.6730	6087.6829	-0.0099	9	4	6	10	8	4	5	9
6087.6730	6087.6792	-0.0062	9	4	6	8	8	4	5	7
6087.7375	6087.7397	-0.0022	9	5	5	9	8	5	4	8
6087.7375	6087.7394	-0.0019	9	4	6	9	8	4	5	8
6162.9901	6163.0105	-0.0204	9	2	7	8	8	2	6	7
6162.9901	6163.0013	-0.0112	9	2	7	10	8	2	6	9
6162.9901	6162.9732	0.0169	9	2	7	9	8	2	6	8
6200.5129	6200.5185	-0.0056	9	4	5	10	8	4	4	9
6200.5129	6200.5120	0.0009	9	4	5	8	8	4	4	7
6200.6182	6200.6052	0.0130	9	4	5	9	8	4	4	8
6314.6932	6314.6980	-0.0048	9	3	6	10	8	3	5	9
6314.6932	6314.7004	-0.0072	9	3	6	8	8	3	5	7
6314.7304	6314.7292	0.0012	9	3	6	9	8	3	5	8
6374.2174	6374.2108	0.0066	10	2	9	11	9	2	8	10
6374.2174	6374.2063	0.0111	10	2	9	10	9	2	8	9
6374.2174	6374.2142	0.0032	10	2	9	9	9	2	8	8
6677.6304	6677.6319	-0.0015	11	1	11	12	10	1	10	11
6677.6304	6677.6306	-0.0002	11	1	11	11	10	1	10	10
6677.6304	6677.6340	-0.0036	11	1	11	10	10	1	10	9
6677.8717	6677.8736	-0.0019	11	0	11	11	10	0	10	10
6677.8717	6677.8771	-0.0054	11	0	11	10	10	0	10	9
6677.8717	6677.8751	-0.0034	11	0	11	12	10	0	10	11
6736.4597	6736.4693	-0.0096	10	7	3	11	9	7	2	10

6736.4597	6736.4533	0.0064	10	7	3	9	9	7	2	8
6753.7349	6753.7372	-0.0023	10	2	8	10	9	2	7	9
6753.7834	6753.7785	0.0049	10	2	8	9	9	2	7	8
6753.7834	6753.7707	0.0127	10	2	8	11	9	2	7	10
6755.6149	6755.6025	0.0124	10	6	5	11	9	6	4	10
6755.7085	6755.7025	0.0060	10	6	5	10	9	6	4	9
6758.0915	6758.0913	0.0002	10	4	7	11	9	4	6	10
6758.0915	6758.0903	0.0012	10	4	7	9	9	4	6	8
6777.3018	6777.2983	0.0035	10	5	6	9	9	5	5	8
6777.3018	6777.3040	-0.0022	10	5	6	11	9	5	5	10
6777.3832	6777.3748	0.0084	10	5	6	10	9	5	5	9
6951.5982	6951.6062	-0.0080	10	4	6	11	9	4	5	10
6951.5982	6951.6029	-0.0047	10	4	6	9	9	4	5	8
6971.0931	6971.0964	-0.0033	11	2	10	11	10	2	9	10
6971.0931	6971.1014	-0.0083	11	2	10	12	10	2	9	11
6971.0931	6971.1042	-0.0111	11	2	10	10	10	2	9	9
6979.4093	6979.4146	-0.0053	11	1	10	10	10	1	9	9
6979.4093	6979.4113	-0.0020	11	1	10	12	10	1	9	11
7006.1360	7006.1298	0.0062	10	3	7	11	9	3	6	10
7006.1360	7006.1341	0.0019	10	3	7	9	9	3	6	8
7006.1360	7006.1369	-0.0009	10	3	7	10	9	3	6	9
7240.8860	7240.8863	-0.0003	11	3	9	12	10	3	8	11
7240.8860	7240.8860	0.0000	11	3	9	11	10	3	8	10
7240.8860	7240.8891	-0.0031	11	3	9	10	10	3	8	9
7271.2372	7271.2245	0.0127	12	0	12	12	11	1	11	11
7271.2372	7271.2273	0.0099	12	0	12	11	11	1	11	10
7271.2372	7271.2256	0.0116	12	0	12	13	11	1	11	12

7271.2817	7271.2862	-0.0045	12	1	12	12	11	1	11	11
7271.2817	7271.2873	-0.0056	12	1	12	13	11	1	11	12
7271.2817	7271.2890	-0.0073	12	1	12	11	11	1	11	10
7271.3733	7271.3842	-0.0109	12	0	12	13	11	0	11	12
7271.3733	7271.3830	-0.0097	12	0	12	12	11	0	11	11
7271.3733	7271.3858	-0.0125	12	0	12	11	11	0	11	10
7537.4002	7537.4191	-0.0189	11	5	6	10	10	5	5	9
7566.1408	7566.1407	0.0001	12	2	11	13	11	2	10	12
7566.1408	7566.1431	-0.0023	12	2	11	11	11	2	10	10
7566.1408	7566.1360	0.0048	12	2	11	12	11	2	10	11
7662.5773	7662.5648	0.0125	11	3	8	10	10	3	7	9
7662.5773	7662.5595	0.0178	11	3	8	12	10	3	7	11
7701.1953	7701.1924	0.0029	11	4	7	12	10	4	6	11
7701.1953	7701.1917	0.0036	11	4	7	10	10	4	6	9
7701.2290	7701.2396	-0.0106	11	4	7	11	10	4	6	10
7864.9054	7864.9033	0.0021	13	1	13	13	12	1	12	12
7864.9054	7864.9057	-0.0003	13	1	13	12	12	1	12	11
7864.9054	7864.9043	0.0011	13	1	13	14	12	1	12	13
7864.9483	7864.9423	0.0060	13	0	13	14	12	0	12	13
7864.9483	7864.9413	0.0070	13	0	13	13	12	0	12	12
7864.9483	7864.9437	0.0046	13	0	13	12	12	0	12	11
7901.4499	7901.4383	0.0116	12	2	10	12	11	2	9	11
7901.4499	7901.4649	-0.0150	12	2	10	11	11	2	9	10
7901.4499	7901.4605	-0.0106	12	2	10	13	11	2	9	12

1-w-E Cluster

Table S12: Observed and calculated rotational transitions (MHz) for 1-w-E

Observed	Calculated	Obs-Calc	J'	K_a'	K_c'	F'	J"	K_a"	K_c"	F"
2072.2032	2072.2029	0.0003	3	1	2	4	2	1	1	3
2072.4060	2072.4119	-0.0059	3	1	2	2	2	1	1	1
2187.1532	2187.1522	0.0010	3	1	3	4	2	0	2	3
2543.3107	2543.3102	0.0005	4	1	4	3	3	1	3	2
2543.3107	2543.3125	-0.0018	4	1	4	4	3	1	3	3
2543.3816	2543.3803	0.0013	4	1	4	5	3	1	3	4
2604.2293	2604.2299	-0.0006	4	0	4	4	3	0	3	3
2604.5094	2604.4964	0.0130	4	0	4	5	3	0	3	4
2656.6060	2656.6050	0.0010	4	2	3	5	3	2	2	4
2656.6060	2656.5966	0.0094	4	2	3	3	3	2	2	2
2713.7866	2713.7827	0.0039	4	2	2	4	3	2	1	3
2754.7400	2754.7416	-0.0016	4	1	3	5	3	1	2	4
2754.8549	2754.8563	-0.0014	4	1	3	3	3	1	2	2
2757.6250	2757.6204	0.0046	4	1	4	4	3	0	3	3
2758.4185	2758.4169	0.0016	4	1	4	5	3	0	3	4
2758.6044	2758.6022	0.0022	4	1	4	3	3	0	3	2
2962.6866	2962.6855	0.0011	3	2	2	4	2	1	1	3
2963.2714	2963.2775	-0.0061	3	2	2	2	2	1	1	1
3068.8127	3068.8145	-0.0018	5	0	5	4	4	1	4	3
3068.9267	3068.9227	0.0040	5	0	5	6	4	1	4	5
3069.1920	3069.1897	0.0023	5	0	5	5	4	1	4	4
3152.1558	3152.1621	-0.0063	3	2	1	2	2	1	2	1
3154.0627	3154.0774	-0.0147	3	2	1	3	2	1	2	2
3170.3625	3170.3683	-0.0058	5	1	5	4	4	1	4	3
3170.3938	3170.4048	-0.0110	5	1	5	6	4	1	4	5

3222.5823	3222.5802	0.0021	5	0	5	5	4	0	4	4
3222.8431	3222.8432	-0.0001	5	0	5	6	4	0	4	5
3222.8783	3222.8713	0.0070	5	0	5	4	4	0	4	3
3313.1299	3313.1167	0.0132	5	2	4	5	4	2	3	4
3313.1299	3313.1469	-0.0170	5	2	4	4	4	2	3	3
3313.1299	3313.1406	-0.0107	5	2	4	6	4	2	3	5
3324.3308	3324.3253	0.0055	5	1	5	6	4	0	4	5
3343.5229	3343.5136	0.0093	5	3	3	6	4	3	2	5
3343.6365	3343.6351	0.0014	5	3	3	5	4	3	2	4
3352.4918	3352.4974	-0.0056	5	3	2	4	4	3	1	3
3352.5233	3352.5302	-0.0069	5	3	2	6	4	3	1	5
3352.7088	3352.7115	-0.0027	5	3	2	5	4	3	1	4
3416.8192	3416.8316	-0.0124	5	2	3	6	4	2	2	5
3416.8192	3416.8189	0.0003	5	2	3	4	4	2	2	3
3417.0666	3417.0713	-0.0047	5	2	3	5	4	2	2	4
3428.7859	3428.7882	-0.0023	5	1	4	5	4	1	3	4
3428.8934	3428.8951	-0.0017	5	1	4	6	4	1	3	5
3428.9657	3428.9720	-0.0063	5	1	4	4	4	1	3	3
3546.0205	3546.0262	-0.0057	4	2	3	4	3	1	2	3
3547.0886	3547.0875	0.0011	4	2	3	5	3	1	2	4
3547.4626	3547.4622	0.0004	4	2	3	3	3	1	2	2
3744.5368	3744.5523	-0.0155	3	3	0	4	2	2	1	3
3793.3250	3793.3303	-0.0053	6	1	6	6	5	1	5	5
3793.4165	3793.4130	0.0035	6	1	6	7	5	1	5	6
3832.1829	3832.1814	0.0015	6	0	6	6	5	0	5	5
3832.4081	3832.4039	0.0042	6	0	6	7	5	0	5	6
3832.4081	3832.4157	-0.0076	6	0	6	5	5	0	5	4

3894.4675	3894.4667	0.0008	6	1	6	6	5	0	5	5
3894.9023	3894.8951	0.0072	6	1	6	7	5	0	5	6
3964.7195	3964.7084	0.0111	6	2	5	6	5	2	4	5
3964.7605	3964.7637	-0.0032	6	2	5	7	5	2	4	6
3964.7605	3964.7730	-0.0125	6	2	5	5	5	2	4	4
4007.5470	4007.5583	-0.0113	6	5	1	6	5	5	0	5
4007.5470	4007.5440	0.0030	6	5	2	6	5	5	1	5
4012.6658	4012.6752	-0.0094	6	4	3	6	5	4	2	5
4013.4829	4013.4784	0.0045	6	4	2	7	5	4	1	6
4013.6191	4013.6151	0.0040	6	4	2	6	5	4	1	5
4014.7913	4014.7970	-0.0057	6	3	4	5	5	3	3	4
4014.7913	4014.8034	-0.0121	6	3	4	7	5	3	3	6
4014.8751	4014.8756	-0.0005	6	3	4	6	5	3	3	5
4037.9871	4038.0067	-0.0196	6	3	3	5	5	3	2	4
4038.0322	4038.0270	0.0052	6	3	3	7	5	3	2	6
4038.1988	4038.2014	-0.0026	6	3	3	6	5	3	2	5
4091.0385	4091.0430	-0.0045	6	1	5	6	5	1	4	5
4091.2030	4091.2040	-0.0010	6	1	5	7	5	1	4	6
4091.2605	4091.2638	-0.0033	6	1	5	5	5	1	4	4
4104.4469	4104.4500	-0.0031	5	2	4	5	4	1	3	4
4105.4872	4105.4865	0.0007	5	2	4	6	4	1	3	5
4105.7530	4105.7529	0.0001	5	2	4	4	4	1	3	3
4123.5031	4123.5139	-0.0108	6	2	4	5	5	2	3	4
4123.5031	4123.5086	-0.0055	6	2	4	7	5	2	3	6
4123.6669	4123.6718	-0.0049	6	2	4	6	5	2	3	5
4376.4543	4376.4672	-0.0129	7	0	7	7	6	1	6	6
4390.1076	4390.1108	-0.0032	4	3	2	4	3	2	1	3

4390.4393	4390.4403	-0.0010	4	3	2	5	3	2	1	4
4390.5588	4390.5598	-0.0010	4	3	2	3	3	2	1	2
4475.1623	4475.1607	0.0016	7	1	7	7	6	0	6	6
4475.4573	4475.4638	-0.0065	7	1	7	6	6	0	6	5
4475.4573	4475.4462	0.0111	7	1	7	8	6	0	6	7
4610.7144	4610.7321	-0.0177	7	2	6	7	6	2	5	6
4610.8184	4610.8179	0.0005	7	2	6	6	6	2	5	5
4610.8184	4610.8077	0.0107	7	2	6	8	6	2	5	7
4640.3728	4640.3702	0.0026	6	2	5	6	5	1	4	5
4641.3532	4641.3552	-0.0020	6	2	5	7	5	1	4	6
4641.5536	4641.5539	-0.0003	6	2	5	5	5	1	4	4
4674.4854	4674.4815	0.0039	7	6	1	6	6	6	0	5
4674.4854	4674.4806	0.0048	7	6	2	6	6	6	1	5
4674.5151	4674.5103	0.0048	7	6	1	8	6	6	0	7
4674.5151	4674.5094	0.0057	7	6	2	8	6	6	1	7
4674.6723	4674.6669	0.0054	7	6	1	7	6	6	0	6
4674.6723	4674.6660	0.0063	7	6	2	7	6	6	1	6
4684.9387	4684.9281	0.0106	7	3	5	8	6	3	4	7
4684.9387	4684.9285	0.0102	7	3	5	6	6	3	4	5
4686.6877	4686.6895	-0.0018	7	4	4	8	6	4	3	7
4686.6877	4686.6785	0.0092	7	4	4	6	6	4	3	5
4686.7929	4686.7861	0.0068	7	4	4	7	6	4	3	6
4689.7394	4689.7542	-0.0148	7	4	3	8	6	4	2	7
4689.7394	4689.7412	-0.0018	7	4	3	6	6	4	2	5
4689.8627	4689.8664	-0.0037	7	4	3	7	6	4	2	6
4738.0281	4738.0304	-0.0023	7	1	6	7	6	1	5	6
4738.2512	4738.2410	0.0102	7	1	6	8	6	1	5	7

4826.2253	4826.2352	-0.0099	7	2	5	8	6	2	4	7
4826.3075	4826.3163	-0.0088	7	2	5	7	6	2	4	6
5009.2334	5009.2286	0.0048	8	0	8	7	7	1	7	6
5009.2334	5009.2364	-0.0030	8	0	8	8	7	1	7	7
5009.2334	5009.2499	-0.0165	8	0	8	9	7	1	7	8
5019.9599	5019.9633	-0.0034	5	3	3	5	4	2	2	4
5020.4743	5020.4726	0.0017	5	3	3	6	4	2	2	5
5020.6113	5020.6126	-0.0013	5	3	3	4	4	2	2	3
5029.7698	5029.7726	-0.0028	8	1	8	7	7	1	7	6
5029.7698	5029.7862	-0.0164	8	1	8	9	7	1	7	8
5045.6486	5045.6445	0.0041	8	0	8	8	7	0	7	7
5045.7839	5045.7721	0.0118	8	0	8	9	7	0	7	8
5045.7839	5045.7674	0.0165	8	0	8	7	7	0	7	6
5066.1271	5066.1204	0.0067	8	1	8	8	7	0	7	7
5066.3170	5066.3114	0.0056	8	1	8	7	7	0	7	6
5066.3170	5066.3084	0.0086	8	1	8	9	7	0	7	8
5104.2951	5104.2863	0.0088	4	4	1	5	3	3	0	4
5104.2951	5104.2837	0.0114	4	4	1	3	3	3	0	2
5104.7600	5104.7468	0.0132	4	4	0	3	3	3	1	2
5104.7600	5104.7530	0.0070	4	4	0	5	3	3	1	4
5119.4461	5119.4552	-0.0091	5	3	2	6	4	2	3	5
5119.6863	5119.6903	-0.0040	5	3	2	5	4	2	3	4
5160.0746	5160.0593	0.0153	7	2	6	7	6	1	5	6
5160.9659	5160.9589	0.0070	7	2	6	8	6	1	5	7
5250.8136	5250.8096	0.0040	8	2	7	8	7	2	6	7
5250.8875	5250.8995	-0.0120	8	2	7	9	7	2	6	8
5250.9199	5250.9084	0.0115	8	2	7	7	7	2	6	6

5341.6961	5341.6864	0.0097	8	7	1	9	7	7	0	8
5341.6961	5341.6864	0.0097	8	7	2	9	7	7	1	8
5341.8310	5341.8283	0.0027	8	7	1	8	7	7	0	7
5341.8310	5341.8283	0.0027	8	7	2	8	7	7	1	7
5345.8210	5345.8333	-0.0123	8	6	2	9	7	6	1	8
5345.8210	5345.8277	-0.0067	8	6	3	9	7	6	2	8
5345.8210	5345.8171	0.0039	8	6	2	7	7	6	1	6
5345.8210	5345.8115	0.0095	8	6	3	7	7	6	2	6
5345.9444	5345.9473	-0.0029	8	6	2	8	7	6	1	7
5345.9444	5345.9416	0.0028	8	6	3	8	7	6	2	7
5352.4101	5352.3908	0.0193	8	3	6	9	7	3	5	8
5352.4101	5352.3943	0.0158	8	3	6	7	7	3	5	6
5352.4101	5352.4004	0.0097	8	3	6	8	7	3	5	7
5352.6707	5352.6784	-0.0077	8	5	4	7	7	5	3	6
5352.6707	5352.6863	-0.0156	8	5	4	9	7	5	3	8
5352.9966	5352.9954	0.0012	8	5	3	9	7	5	2	8
5352.9966	5352.9849	0.0117	8	5	3	7	7	5	2	6
5362.4376	5362.4453	-0.0077	8	4	5	9	7	4	4	8
5362.4376	5362.4397	-0.0021	8	4	5	7	7	4	4	6
5362.5247	5362.5210	0.0037	8	4	5	8	7	4	4	7
5367.9684	5367.9814	-0.0130	8	1	7	8	7	1	6	7
5368.2313	5368.2262	0.0051	8	1	7	9	7	1	6	8
5370.6454	5370.6453	0.0001	8	4	4	7	7	4	3	6
5370.6454	5370.6544	-0.0090	8	4	4	9	7	4	3	8
5370.7634	5370.7613	0.0021	8	4	4	8	7	4	3	7
5441.7214	5441.7139	0.0075	8	3	5	9	7	3	4	8
5441.9007	5441.9048	-0.0041	8	3	5	8	7	3	4	7

5519.6556	5519.6643	-0.0087	8	2	6	8	7	2	5	7
5519.6556	5519.6566	-0.0010	8	2	6	9	7	2	5	8
5617.7652	5617.7676	-0.0024	6	3	4	6	5	2	3	5
5618.4473	5618.4444	0.0029	6	3	4	7	5	2	3	6
5618.5961	5618.5908	0.0053	6	3	4	5	5	2	3	4
5633.4113	5633.4161	-0.0048	9	0	9	9	8	1	8	8
5633.4619	5633.4516	0.0103	9	0	9	10	8	1	8	9
5644.6000	5644.6054	-0.0054	9	1	9	9	8	1	8	8
5644.6720	5644.6605	0.0115	9	1	9	8	8	1	8	7
5644.6720	5644.6718	0.0002	9	1	9	10	8	1	8	9
5653.9038	5653.8921	0.0117	9	0	9	9	8	0	8	8
5653.9970	5653.9808	0.0162	9	0	9	8	8	0	8	7
5653.9970	5653.9879	0.0091	9	0	9	10	8	0	8	9
5665.0863	5665.0814	0.0049	9	1	9	9	8	0	8	8
5665.2119	5665.2044	0.0075	9	1	9	8	8	0	8	7
5665.2119	5665.2081	0.0038	9	1	9	10	8	0	8	9
5672.8285	5672.8385	-0.0100	8	2	7	8	7	1	6	7
5673.6147	5673.6174	-0.0027	8	2	7	9	7	1	6	8
5673.7260	5673.7260	-0.0000	8	2	7	7	7	1	6	6
5677.5203	5677.5392	-0.0189	9	1	8	10	8	2	7	9
5677.5203	5677.5045	0.0158	9	1	8	8	8	2	7	7
5677.8159	5677.8208	-0.0049	9	1	8	9	8	2	7	8
5769.4708	5769.4576	0.0132	5	4	2	5	4	3	1	4
5769.5568	5769.5508	0.0060	5	4	2	6	4	3	1	5
5769.5568	5769.5742	-0.0174	5	4	2	4	4	3	1	3
5772.8082	5772.7943	0.0139	5	4	1	5	4	3	2	4
5772.8683	5772.8622	0.0061	5	4	1	4	4	3	2	3

5772.8683	5772.8488	0.0195	5	4	1	6	4	3	2	5
5844.2592	5844.2686	-0.0094	6	3	3	5	5	2	4	4
5844.3384	5844.3415	-0.0031	6	3	3	7	5	2	4	6
5844.7690	5844.7750	-0.0060	6	3	3	6	5	2	4	5
5885.0426	5885.0486	-0.0060	9	2	8	8	8	2	7	7
5885.0426	5885.0407	0.0019	9	2	8	10	8	2	7	9
5982.6765	5982.6779	-0.0014	9	1	8	9	8	1	7	8
5982.9484	5982.9303	0.0181	9	1	8	10	8	1	7	9
5982.9489	5982.9631	-0.0142	9	1	8	8	8	1	7	7
6012.6585	6012.6714	-0.0129	9	7	3	10	8	7	2	9
6012.6585	6012.6718	-0.0133	9	7	2	10	8	7	1	9
6012.6585	6012.6577	0.0008	9	7	3	8	8	7	2	7
6012.6585	6012.6581	0.0004	9	7	2	8	8	7	1	7
6012.7726	6012.7783	-0.0057	9	7	2	9	8	7	1	8
6012.7726	6012.7779	-0.0053	9	7	3	9	8	7	2	8
6015.7321	6015.7221	0.0100	9	3	7	9	8	3	6	8
6015.7322	6015.7428	-0.0106	9	3	7	8	8	3	6	7
6015.7325	6015.7365	-0.0040	9	3	7	10	8	3	6	9
6039.1751	6039.1847	-0.0096	9	4	6	10	8	4	5	9
6039.1751	6039.1822	-0.0071	9	4	6	8	8	4	5	7
6039.2373	6039.2440	-0.0067	9	4	6	9	8	4	5	8
6058.1132	6058.0999	0.0133	9	4	5	8	8	4	4	7
6058.1132	6058.1078	0.0054	9	4	5	10	8	4	4	9
6058.2345	6058.2223	0.0122	9	4	5	9	8	4	4	8
6158.0876	6158.0894	-0.0018	9	3	6	10	8	3	5	9
6158.0876	6158.0849	0.0027	9	3	6	8	8	3	5	7
6158.2722	6158.2709	0.0013	9	3	6	9	8	3	5	8

6179.0555	6179.0619	-0.0064	7	3	5	7	6	2	4	6
6179.8553	6179.8639	-0.0086	7	3	5	8	6	2	4	7
6180.0017	6180.0054	-0.0037	7	3	5	6	6	2	4	5
6189.7938	6189.7996	-0.0058	9	2	8	9	8	1	7	8
6190.4173	6190.4319	-0.0146	9	2	8	10	8	1	7	9
6190.5047	6190.5072	-0.0025	9	2	8	8	8	1	7	7
6200.1171	6200.1101	0.0070	9	2	7	9	8	2	6	8
6200.1566	6200.1668	-0.0102	9	2	7	10	8	2	6	9
6252.2478	6252.2633	-0.0155	10	0	10	11	9	1	9	10
6252.2478	6252.2519	-0.0041	10	0	10	9	9	1	9	8
6258.2393	6258.2505	-0.0112	10	1	10	9	9	1	9	8
6263.4089	6263.4094	-0.0005	10	0	10	10	9	0	9	9
6263.4879	6263.4756	0.0123	10	0	10	9	9	0	9	8
6263.4879	6263.4835	0.0044	10	0	10	11	9	0	9	10
6269.3962	6269.3910	0.0052	10	1	10	10	9	0	9	9
6269.4829	6269.4742	0.0087	10	1	10	9	9	0	9	8
6269.4829	6269.4805	0.0024	10	1	10	11	9	0	9	10
6380.2090	6380.2058	0.0032	10	1	9	9	9	2	8	8
6380.2090	6380.2242	-0.0152	10	1	9	11	9	2	8	10
6380.3602	6380.3713	-0.0111	10	1	9	10	9	2	8	9
6429.4320	6429.4213	0.0107	6	4	3	6	5	3	2	5
6429.5849	6429.5986	-0.0137	6	4	3	5	5	3	2	4
6429.5849	6429.5651	0.0198	6	4	3	7	5	3	2	6
6442.8108	6442.8262	-0.0154	6	4	2	5	5	3	3	4
6442.8108	6442.8136	-0.0028	6	4	2	7	5	3	3	6
6513.5395	6513.5255	0.0140	10	2	9	10	9	2	8	9
6587.4808	6587.4930	-0.0122	10	1	9	10	9	1	8	9

6613.5916	6613.5929	-0.0013	7	3	4	6	6	2	5	5
6613.6938	6613.6913	0.0025	7	3	4	8	6	2	5	7
6614.3640	6614.3665	-0.0025	7	3	4	7	6	2	5	6
6673.7267	6673.7403	-0.0136	10	3	8	9	9	3	7	8
6673.7267	6673.7336	-0.0069	10	3	8	11	9	3	7	10
6679.6079	6679.6015	0.0064	10	8	2	9	9	8	1	8
6679.6079	6679.6015	0.0064	10	8	3	9	9	8	2	8
6679.7125	6679.7132	-0.0007	10	8	2	10	9	8	1	9
6679.7125	6679.7131	-0.0006	10	8	3	10	9	8	2	9
6684.8107	6684.8145	-0.0038	10	7	4	11	9	7	3	10
6684.8107	6684.8057	0.0050	10	7	4	9	9	7	3	8
6684.8107	6684.8164	-0.0057	10	7	3	11	9	7	2	10
6684.8107	6684.8077	0.0030	10	7	3	9	9	7	2	8
6684.9052	6684.9008	0.0044	10	7	3	10	9	7	2	9
6684.9052	6684.8989	0.0063	10	7	4	10	9	7	3	9
6705.1384	6705.1461	-0.0077	8	3	6	8	7	2	5	7
6705.5665	6705.5611	0.0054	10	5	6	9	9	5	5	8
6705.5665	6705.5653	0.0012	10	5	6	11	9	5	5	10
6705.6412	6705.6331	0.0081	10	5	6	10	9	5	5	9
6706.0098	6706.0195	-0.0097	8	3	6	9	7	2	5	8
6706.1417	6706.1504	-0.0087	8	3	6	7	7	2	5	6
6708.2496	6708.2526	-0.0030	10	5	5	11	9	5	4	10
6708.2496	6708.2476	0.0020	10	5	5	9	9	5	4	8
6708.3321	6708.3292	0.0029	10	5	5	10	9	5	4	9
6715.8933	6715.8829	0.0104	10	4	7	9	9	4	6	8
6715.8933	6715.8836	0.0097	10	4	7	11	9	4	6	10
6754.4912	6754.4916	-0.0004	10	4	6	11	9	4	5	10

6754.4912	6754.4831	0.0081	10	4	6	9	9	4	5	8
6754.6248	6754.6218	0.0030	10	4	6	10	9	4	5	9
6864.7059	6864.6993	0.0066	10	2	8	10	9	2	7	9
6864.8074	6864.8135	-0.0061	10	2	8	11	9	2	7	10
6871.0294	6871.0259	0.0035	11	1	11	10	10	1	10	9
6871.0294	6871.0346	-0.0052	11	1	11	12	10	1	10	11
6873.8812	6873.8735	0.0077	11	0	11	10	10	0	10	9
6873.8812	6873.8813	-0.0001	11	0	11	12	10	0	10	11
6876.9654	6876.9649	0.0005	11	1	11	11	10	0	10	10
6877.0312	6877.0246	0.0066	11	1	11	10	10	0	10	9
6877.0312	6877.0316	-0.0004	11	1	11	12	10	0	10	11
7055.4836	7055.4652	0.0184	11	1	10	10	10	2	9	9
7055.4836	7055.4738	0.0098	11	1	10	12	10	2	9	11
7078.0236	7078.0061	0.0175	7	4	4	7	6	3	3	6
7078.2643	7078.2704	-0.0061	7	4	4	6	6	3	3	5
7117.7805	7117.7703	0.0102	7	4	3	6	6	3	4	5
7117.7805	7117.7644	0.0161	7	4	3	8	6	3	4	7
7117.7805	7117.7651	0.0154	7	4	3	7	6	3	4	6
7134.6992	7134.7161	-0.0169	6	5	1	6	5	4	2	5
7188.6718	7188.6791	-0.0073	11	1	10	11	10	1	9	10
7188.8774	7188.8935	-0.0161	11	1	10	10	10	1	9	9
7188.8774	7188.8769	0.0005	11	1	10	12	10	1	9	11
7201.1993	7201.2038	-0.0045	9	3	7	9	8	2	6	8
7202.1023	7202.0995	0.0028	9	3	7	10	8	2	6	9
7202.2159	7202.2164	-0.0005	9	3	7	8	8	2	6	7
7270.4307	7270.4393	-0.0086	11	2	10	11	10	1	9	10
7270.8250	7270.8201	0.0049	11	2	10	10	10	1	9	9

7346.5943	7346.6014	-0.0071	11	9	3	12	10	9	2	11
7346.5943	7346.6014	-0.0071	11	9	2	12	10	9	1	11
7346.6969	7346.6954	0.0015	11	9	2	11	10	9	1	10
7346.6969	7346.6954	0.0015	11	9	3	11	10	9	2	10
7351.3270	7351.3309	-0.0039	11	8	3	12	10	8	2	11
7351.3270	7351.3231	0.0039	11	8	3	10	10	8	2	9
7351.3270	7351.3308	-0.0038	11	8	4	12	10	8	3	11
7351.3270	7351.3229	0.0041	11	8	4	10	10	8	3	9
7351.4166	7351.4112	0.0054	11	8	3	11	10	8	2	10
7351.4166	7351.4111	0.0055	11	8	4	11	10	8	3	10
7358.2549	7358.2560	-0.0011	11	7	5	12	10	7	4	11
7358.2549	7358.2581	-0.0032	11	7	4	10	10	7	3	9
7358.2549	7358.2499	0.0050	11	7	5	10	10	7	4	9
7358.2549	7358.2641	-0.0092	11	7	4	12	10	7	3	11
7358.3335	7358.3262	0.0073	11	7	5	11	10	7	4	10
7358.3335	7358.3344	-0.0009	11	7	4	11	10	7	3	10
7384.8380	7384.8427	-0.0047	11	5	7	12	10	5	6	11
7384.8380	7384.8399	-0.0019	11	5	7	10	10	5	6	9
7384.8828	7384.9028	-0.0200	11	5	7	11	10	5	6	10
7391.1919	7391.1951	-0.0032	11	4	8	10	10	4	7	9
7391.1919	7391.1934	-0.0015	11	4	8	12	10	4	7	11
7391.3602	7391.3551	0.0051	11	5	6	12	10	5	5	11
7391.3602	7391.3509	0.0093	11	5	6	10	10	5	5	9
7391.4409	7391.4326	0.0083	11	5	6	11	10	5	5	10
7444.4560	7444.4722	-0.0162	8	3	5	7	7	2	6	6
7444.5898	7444.5976	-0.0078	8	3	5	9	7	2	6	8
7445.5355	7445.5392	-0.0037	8	3	5	8	7	2	6	7

7461.9402	7461.9318	0.0084	11	4	7	12	10	4	6	11
7461.9402	7461.9244	0.0158	11	4	7	10	10	4	6	9
7462.0948	7462.0813	0.0135	11	4	7	11	10	4	6	10
7481.6962	7481.6816	0.0146	12	0	12	11	11	1	11	10
7481.6962	7481.6897	0.0065	12	0	12	13	11	1	11	12
7483.3278	7483.3216	0.0062	12	1	12	13	11	1	11	12
7483.3281	7483.3138	0.0143	12	1	12	11	11	1	11	10
7484.8426	7484.8326	0.0100	12	0	12	11	11	0	11	10
7484.8426	7484.8400	0.0026	12	0	12	13	11	0	11	12
7486.4718	7486.4648	0.0070	12	1	12	11	11	0	11	10
7486.4718	7486.4719	-0.0001	12	1	12	13	11	0	11	12
7510.9735	7510.9746	-0.0011	11	2	9	11	10	2	8	10
7511.1266	7511.1387	-0.0121	11	2	9	12	10	2	8	11
7589.7900	7589.7794	0.0106	11	3	8	12	10	3	7	11
7589.7900	7589.7824	0.0076	11	3	8	10	10	3	7	9
7589.8790	7589.8616	0.0174	11	3	8	11	10	3	7	10
7674.7851	7674.7908	-0.0057	10	3	8	10	9	2	7	9
7675.6630	7675.6663	-0.0033	10	3	8	11	9	2	7	10
7675.7625	7675.7670	-0.0045	10	3	8	9	9	2	7	8
7706.2372	7706.2271	0.0101	8	4	5	8	7	3	4	7
7708.7437	7708.7448	-0.0011	12	1	11	11	11	2	10	10
7708.7437	7708.7391	0.0046	12	1	11	12	11	2	10	11
7708.7437	7708.7481	-0.0044	12	1	11	13	11	2	10	12
7757.1300	7757.1443	-0.0143	12	2	11	12	11	2	10	11
7757.2297	7757.2419	-0.0122	12	2	11	13	11	2	10	12
7757.2297	7757.2461	-0.0164	12	2	11	11	11	2	10	10
7790.4880	7790.4993	-0.0113	12	1	11	12	11	1	10	11

7790.6574	7790.6607	-0.0033	12	1	11	13	11	1	10	12
7790.6574	7790.6714	-0.0140	12	1	11	11	11	1	10	10
7839.1440	7839.1545	-0.0105	12	2	11	13	11	1	10	12
7970.4573	7970.4736	-0.0163	12	3	10	12	11	3	9	11
7970.5381	7970.5507	-0.0126	12	3	10	11	11	3	9	10
7970.5381	7970.5435	-0.0054	12	3	10	13	11	3	9	12

2-w-E Cluster

Table S13: Observed and calculated rotational transitions (MHz) for 2-w-E

Observed	Calculated	Obs-Calc	J'	K_a'	K_c'	F'	J"	K_a"	K_c"	F"
2555.7019	2555.6954	0.0065	4	1	3	5	3	1	2	4
2891.7571	2891.7575	-0.0004	5	0	5	6	4	1	4	5
3014.1032	3014.1009	0.0023	5	1	5	6	4	0	4	5
3060.1605	3060.1512	0.0093	3	3	1	2	2	2	0	1
3060.2324	3060.2173	0.0151	3	3	1	4	2	2	0	3
3073.1436	3073.1298	0.0138	5	2	4	6	4	2	3	5
3487.6300	3487.6248	0.0052	6	0	6	7	5	1	5	6
3511.2835	3511.2670	0.0165	6	1	6	7	5	1	5	6
3555.7698	3555.7862	-0.0164	13	8	5	14	13	7	6	14
3555.7711	3555.7748	-0.0037	6	1	6	5	5	0	5	4
3555.7711	3555.7728	-0.0017	6	1	6	7	5	0	5	6
4007.5470	4007.5521	-0.0051	16	9	7	17	16	8	8	17
4071.3343	4071.3225	0.0118	7	0	7	8	6	1	6	7
4166.0939	4166.1005	-0.0066	4	4	1	5	3	3	0	4
4267.7840	4267.7820	0.0020	31	9	23	31	31	8	24	31

4267.7840	4267.7788	0.0052	7	2	6	8	6	2	5	7
4331.7833	4331.7790	0.0043	8	2	6	9	7	3	5	8
4331.8280	4331.8135	0.0145	8	2	6	7	7	3	5	6
4349.4346	4349.4327	0.0019	7	6	1	8	6	6	0	7
4349.5524	4349.5611	-0.0087	7	6	2	7	6	6	1	6
4354.5835	4354.5903	-0.0068	7	3	5	8	6	3	4	7
4354.6527	4354.6526	0.0001	84	31	54	84	85	26	59	85
4363.7816	4363.7856	-0.0040	7	4	4	8	6	4	3	7
4363.8375	4363.8321	0.0054	7	4	4	7	6	4	3	6
4371.0327	4371.0442	-0.0115	7	4	3	8	6	4	2	7
4425.8766	4425.8875	-0.0109	20	10	10	20	20	9	11	20
4426.9215	4426.8989	0.0226	10	3	8	11	9	4	6	10
4492.5880	4492.5752	0.0128	7	2	5	8	6	2	4	7
4548.7145	4548.6983	0.0162	13	10	4	13	13	9	5	13
4548.7770	4548.7595	0.0175	13	10	4	14	13	9	5	14
4552.5317	4552.5373	-0.0056	7	2	6	8	6	1	5	7
4555.8774	4555.8777	-0.0003	12	10	3	13	12	9	4	13
4647.4086	4647.4039	0.0047	8	0	8	9	7	1	7	8
4653.2106	4653.2022	0.0084	8	1	8	9	7	1	7	8
4659.3462	4659.3296	0.0166	8	0	8	9	7	0	7	8
4741.7458	4741.7543	-0.0085	11	2	10	11	10	3	8	10
4776.8237	4776.8289	-0.0052	6	3	4	6	5	2	3	5
4783.3824	4783.3860	-0.0036	5	4	2	5	4	3	1	4
4783.4299	4783.4148	0.0151	5	4	2	6	4	3	1	5
4783.9500	4783.9578	-0.0078	5	4	1	5	4	3	1	4
4788.8489	4788.8421	0.0068	5	4	2	6	4	3	2	5
4789.4100	4789.4167	-0.0067	5	4	1	6	4	3	2	5

4970.1956	4970.2090	-0.0134	8	3	6	9	7	3	5	8
4975.3942	4975.4134	-0.0192	8	6	2	9	7	6	1	8
4975.4806	4975.4975	-0.0169	8	6	2	8	7	6	1	7
4984.0547	4984.0507	0.0040	8	5	4	9	7	5	3	8
4984.1060	4984.1010	0.0050	8	5	4	8	7	5	3	7
4985.0517	4985.0565	-0.0048	8	5	3	9	7	5	2	8
4985.1187	4985.1043	0.0144	8	5	3	8	7	5	2	7
4993.4241	4993.4246	-0.0005	8	4	5	9	7	4	4	8
5012.3365	5012.3487	-0.0122	8	4	4	9	7	4	3	8
5047.2260	5047.2303	-0.0043	8	2	7	9	7	1	6	8
5100.4891	5100.5075	-0.0184	8	3	5	9	7	3	4	8
5180.4506	5180.4664	-0.0158	7	2	5	7	6	1	5	6
5180.7601	5180.7705	-0.0104	7	2	5	8	6	1	5	7
5219.1911	5219.1824	0.0087	9	0	9	10	8	1	8	9
5224.9998	5224.9807	0.0191	9	0	9	10	8	0	8	9
5227.7270	5227.7242	0.0028	9	1	9	10	8	0	8	9
5367.6683	5367.6766	-0.0083	9	1	8	10	8	2	7	9
5413.2305	5413.2240	0.0065	6	4	3	7	5	3	3	6
5416.0418	5416.0494	-0.0076	6	4	2	7	5	3	3	6
5420.7296	5420.7364	-0.0068	7	3	4	6	6	2	4	5
5555.8467	5555.8302	0.0165	9	2	8	10	8	1	7	9
5579.5043	5579.4901	0.0142	9	3	7	10	8	3	6	9
5590.7215	5590.7434	-0.0219	9	8	1	10	8	8	0	9
5595.6058	5595.6088	-0.0030	9	7	2	10	8	7	1	9
5595.6800	5595.6912	-0.0112	9	7	2	9	8	7	1	8
5603.2398	5603.2479	-0.0081	9	6	4	9	8	6	3	8
5614.9400	5614.9298	0.0102	9	5	5	10	8	5	4	9

5618.1030	5618.0994	0.0036	9	5	4	10	8	5	3	9
5622.5875	5622.5878	-0.0003	9	4	6	10	8	4	5	9
5622.6204	5622.6089	0.0115	9	4	6	9	8	4	5	8
5664.3584	5664.3604	-0.0020	9	4	5	10	8	4	4	9
5760.4772	5760.4943	-0.0171	8	3	6	9	7	2	5	8
5788.6915	5788.6910	0.0005	10	0	10	11	9	1	9	10
5789.9656	5789.9623	0.0033	10	1	10	11	9	1	9	10
5791.4335	5791.4346	-0.0011	10	0	10	11	9	0	9	10
5792.7128	5792.7059	0.0069	10	1	10	11	9	0	9	10
5888.3926	5888.3972	-0.0046	6	5	2	7	5	4	1	6
5889.0315	5889.0239	0.0076	6	5	1	7	5	4	2	6
5942.3562	5942.3516	0.0046	8	2	6	9	7	1	6	8
5975.4592	5975.4553	0.0039	10	1	9	11	9	2	8	10
6013.1734	6013.1581	0.0153	10	2	9	11	9	2	8	10
6042.8727	6042.8700	0.0027	7	4	4	8	6	3	4	7
6052.9428	6052.9541	-0.0113	7	4	3	8	6	3	4	7
6181.5099	6181.4920	0.0179	10	3	8	11	9	3	7	10
6215.9806	6215.9867	-0.0061	10	8	2	11	9	8	1	10
6216.0585	6216.0665	-0.0080	10	8	2	10	9	8	1	9
6217.9142	6217.9201	-0.0059	9	3	7	10	8	2	6	9
6218.2685	6218.2805	-0.0120	9	3	7	9	8	2	6	8
6222.6705	6222.6904	-0.0199	10	7	3	11	9	7	2	10
6222.7541	6222.7468	0.0073	10	7	3	10	9	7	2	9
6247.7194	6247.7255	-0.0061	10	5	6	11	9	5	5	10
6249.4051	6249.4005	0.0046	10	4	7	11	9	4	6	10
6256.2136	6256.2119	0.0017	10	5	5	11	9	5	4	10
6329.0953	6329.0999	-0.0046	10	4	6	11	9	4	5	10

6357.0576	6357.0606	-0.0030	11	0	11	12	10	1	10	11
6357.6419	6357.6400	0.0019	11	1	11	12	10	1	10	11
6358.3280	6358.3319	-0.0039	11	0	11	12	10	0	10	11
6358.9116	6358.9113	0.0003	11	1	11	12	10	0	10	11
6370.1423	6370.1383	0.0040	6	6	1	7	5	5	0	6
6426.2018	6426.1973	0.0045	10	3	7	11	9	3	6	10
6506.4023	6506.4020	0.0003	7	5	3	8	6	4	2	7
6509.5358	6509.5363	-0.0005	7	5	2	8	6	4	3	7
6543.7906	6543.8081	-0.0175	8	4	5	9	7	3	4	8
6566.3105	6566.3101	0.0004	11	1	10	12	10	2	9	11
6586.0974	6586.0843	0.0131	11	2	10	12	10	2	9	11
6604.0303	6604.0129	0.0174	11	1	10	12	10	1	9	11
6666.3486	6666.3640	-0.0154	10	3	8	11	9	2	7	10
6674.8424	6674.8413	0.0011	9	3	6	10	8	2	6	9
6681.6078	6681.6145	-0.0067	8	4	5	8	7	3	5	7
6681.6976	6681.7044	-0.0068	8	4	5	9	7	3	5	8
6775.9476	6775.9331	0.0145	11	3	9	12	10	3	8	11
6842.5119	6842.5209	-0.0090	11	8	3	12	10	8	2	11
6842.5739	6842.5781	-0.0042	11	8	3	11	10	8	2	10
6851.5703	6851.5567	0.0136	11	7	4	11	10	7	3	10
6866.4843	6866.4724	0.0119	11	6	5	12	10	6	4	11
6871.8859	6871.8679	0.0180	11	4	8	12	10	4	7	11
6881.6791	6881.6957	-0.0166	11	5	7	12	10	5	6	11
6901.5927	6901.5993	-0.0066	11	5	6	12	10	5	5	11
6924.8751	6924.8804	-0.0053	12	0	12	13	11	1	11	12
6925.1250	6925.1410	-0.0160	12	1	12	13	11	1	11	12
6925.4514	6925.4599	-0.0085	12	0	12	13	11	0	11	12

6925.7113	6925.7204	-0.0091	12	1	12	13	11	0	11	12
7004.5774	7004.5837	-0.0063	11	4	7	12	10	4	6	11
7067.9968	7067.9925	0.0043	11	3	8	12	10	3	7	11
7119.2390	7119.2517	-0.0127	11	3	9	12	10	2	8	11
7119.4170	7119.4085	0.0085	8	5	4	9	7	4	3	8
7119.5427	7119.5329	0.0098	11	3	9	11	10	2	8	10
7120.7347	7120.7232	0.0115	8	5	3	9	7	4	3	8
7129.5046	7129.4925	0.0121	8	5	4	9	7	4	4	8
7130.8189	7130.8072	0.0117	8	5	3	9	7	4	4	8
7146.5231	7146.5188	0.0043	12	1	11	13	11	2	10	12
7156.5667	7156.5624	0.0043	12	2	11	13	11	2	10	12
7224.2474	7224.2600	-0.0126	12	2	10	13	11	3	9	12
7333.9603	7333.9703	-0.0100	9	4	6	9	8	3	6	8
7334.0623	7334.0831	-0.0208	9	4	6	10	8	3	6	9
7363.2132	7363.1975	0.0157	12	3	10	13	11	3	9	12
7462.6104	7462.6020	0.0084	12	9	3	13	11	9	2	12
7462.6104	7462.6019	0.0085	12	9	4	13	11	9	3	12
7471.9118	7471.9166	-0.0048	7	7	0	8	6	6	1	7
7488.2217	7488.2128	0.0089	12	4	9	13	11	4	8	12
7492.4342	7492.4437	-0.0095	13	0	13	14	12	1	12	13
7492.5538	7492.5595	-0.0057	13	1	13	14	12	1	12	13
7492.6933	7492.7042	-0.0109	13	0	13	14	12	0	12	13
7492.8107	7492.8200	-0.0093	13	1	13	14	12	0	12	13
7499.4300	7499.4398	-0.0098	12	6	7	13	11	6	6	12
7503.0574	7503.0646	-0.0072	12	6	6	13	11	6	5	12
7515.5831	7515.5734	0.0097	12	5	8	13	11	5	7	12
7557.1040	7557.1174	-0.0134	12	5	7	13	11	5	6	12

7589.1460	7589.1563	-0.0103	12	3	10	13	11	2	9	12
7610.4213	7610.4131	0.0082	8	6	3	9	7	5	2	8
7610.7585	7610.7517	0.0068	8	6	2	9	7	5	3	8
7683.1854	7683.1921	-0.0067	12	4	8	13	11	4	7	12
7689.4873	7689.4671	0.0202	12	3	9	13	11	3	8	12
7697.5350	7697.5526	-0.0176	10	4	6	11	9	3	6	10
7720.5553	7720.5494	0.0059	13	1	12	14	12	2	11	13
7722.0005	7721.9896	0.0109	9	5	5	10	8	4	4	9
7725.5278	7725.5236	0.0042	13	2	12	14	12	2	11	13
7730.6047	7730.5930	0.0117	13	1	12	14	12	1	11	13
7735.5741	7735.5672	0.0069	13	2	12	14	12	1	11	13
7751.0068	7750.9978	0.0090	9	5	5	10	8	4	5	9
7755.4412	7755.4395	0.0017	9	5	4	9	8	4	5	8
7755.4892	7755.4820	0.0072	9	5	4	10	8	4	5	9
7863.5315	7863.5382	-0.0067	13	2	11	14	12	3	10	13
7944.2386	7944.2262	0.0124	13	3	11	14	12	3	10	13

^{13}C Isotopologues of the Z Isomer

Table S14: Observed and calculated rotational transitions (MHz) for the ^{13}C (atom number 1) isotopologue of the Z isomer

Observed	Calculated	Obs-Calc	J'	K_a'	K_c'	F'	J"	K_a"	K_c"	F"
2971.8568	2971.8615	-0.0047	4	1	3	5	3	1	2	4
2971.9197	2971.9187	0.0010	4	1	3	4	3	1	2	3
3671.3436	3671.3549	-0.0113	5	0	5	5	4	0	4	4
3671.4378	3671.4391	-0.0013	5	0	5	6	4	0	4	5
3714.2712	3714.2840	-0.0128	5	1	4	6	4	1	3	5

3714.3560	3714.3686	-0.0126	5	1	4	4	4	1	3	3
4364.3150	4364.3192	-0.0042	6	1	6	6	5	1	5	5
4364.3150	4364.3108	0.0042	6	1	6	5	5	1	5	4
4364.3545	4364.3452	0.0093	6	1	6	7	5	1	5	6
4411.3213	4411.3319	-0.0106	6	2	5	7	5	2	4	6
4411.3213	4411.3340	-0.0127	6	2	5	5	5	2	4	4
4456.2948	4456.3009	-0.0061	6	1	5	6	5	1	4	5
4456.2948	4456.3002	-0.0054	6	1	5	7	5	1	4	6
4456.3616	4456.3583	0.0033	6	1	5	5	5	1	4	4
5090.6940	5090.7098	-0.0158	7	1	7	6	6	1	6	5
5090.6940	5090.7009	-0.0069	7	1	7	7	6	1	6	6
5132.1447	5132.1493	-0.0046	7	0	7	8	6	0	6	7
5132.1447	5132.1671	-0.0224	7	0	7	6	6	0	6	5
5145.8254	5145.8241	0.0013	7	2	6	8	6	2	5	7
5145.8254	5145.8336	-0.0082	7	2	6	7	6	2	5	6
5145.8254	5145.8285	-0.0031	7	2	6	6	6	2	5	5
5161.7608	5161.7612	-0.0004	7	2	5	8	6	2	4	7
5161.7608	5161.7566	0.0042	7	2	5	6	6	2	4	5
5161.8849	5161.8926	-0.0077	7	2	5	7	6	2	4	6
5197.8191	5197.8011	0.0180	7	1	6	7	6	1	5	6
5197.8191	5197.8138	0.0053	7	1	6	8	6	1	5	7
5816.6679	5816.6725	-0.0046	8	1	8	7	7	1	7	6
5816.6679	5816.6566	0.0113	8	1	8	8	7	1	7	7
5859.8089	5859.8058	0.0031	8	0	8	8	7	0	7	7
5859.9250	5859.9379	-0.0129	8	0	8	9	7	0	7	8
5879.9737	5879.9754	-0.0017	8	2	7	9	7	2	6	8
5879.9737	5879.9802	-0.0065	8	2	7	7	7	2	6	6

5879.9737	5879.9719	0.0018	8	2	7	8	7	2	6	7
5886.5973	5886.5807	0.0166	8	3	6	7	7	3	5	6
5886.5973	5886.5831	0.0142	8	3	6	9	7	3	5	8
5903.5528	5903.5566	-0.0038	8	2	6	9	7	2	5	8
5903.5528	5903.5532	-0.0004	8	2	6	7	7	2	5	6
5903.6816	5903.6875	-0.0059	8	2	6	8	7	2	5	7
5938.7357	5938.7498	-0.0141	8	1	7	7	7	1	6	6
6542.1547	6542.1741	-0.0194	9	1	9	9	8	1	8	8
6542.2211	6542.2112	0.0099	9	1	9	10	8	1	8	9
6585.7647	6585.7658	-0.0011	9	0	9	9	8	0	8	8
6585.9074	6585.9072	0.0002	9	0	9	10	8	0	8	9
6585.9074	6585.9199	-0.0125	9	0	9	8	8	0	8	7
6620.8473	6620.8463	0.0010	9	5	4	10	8	5	3	9
6620.8473	6620.8363	0.0110	9	5	4	8	8	5	3	7
6620.8473	6620.8361	0.0112	9	5	5	8	8	5	4	7
6620.8473	6620.8461	0.0012	9	5	5	10	8	5	4	9
6620.9449	6620.9374	0.0075	9	5	4	9	8	5	3	8
6620.9449	6620.9372	0.0077	9	5	5	9	8	5	4	8
6621.7364	6621.7282	0.0082	9	4	6	10	8	4	5	9
6621.7364	6621.7249	0.0115	9	4	6	8	8	4	5	7
6621.7364	6621.7516	-0.0152	9	4	5	8	8	4	4	7
6621.7364	6621.7504	-0.0140	9	4	5	10	8	4	4	9
6621.7971	6621.7933	0.0038	9	4	6	9	8	4	5	8
6621.7971	6621.8141	-0.0170	9	4	5	9	8	4	4	8
6623.0753	6623.0886	-0.0133	9	3	7	9	8	3	6	8
6624.6216	6624.6182	0.0034	9	3	6	8	8	3	5	7
6624.6216	6624.6200	0.0016	9	3	6	10	8	3	5	9

6646.9006	6646.9049	-0.0043	9	2	7	9	8	2	6	8
6678.8510	6678.8489	0.0021	9	1	8	9	8	1	7	8
6678.9250	6678.9131	0.0119	9	1	8	8	8	1	7	7
7267.2318	7267.2234	0.0084	10	1	10	10	9	1	9	9
7267.2671	7267.2525	0.0146	10	1	10	9	9	1	9	8
7267.2671	7267.2625	0.0046	10	1	10	11	9	1	9	10
7309.9625	7309.9551	0.0074	10	0	10	10	9	0	9	9
7310.1085	7310.1016	0.0069	10	0	10	11	9	0	9	10
7310.1085	7310.1123	-0.0038	10	0	10	9	9	0	9	8
7359.6385	7359.6386	-0.0001	10	3	8	9	9	3	7	8
7359.6385	7359.6381	0.0004	10	3	8	11	9	3	7	10
7362.3404	7362.3304	0.0100	10	3	7	11	9	3	6	10
7362.3404	7362.3292	0.0112	10	3	7	9	9	3	6	8
7391.4409	7391.4475	-0.0066	10	2	8	10	9	2	7	9
7418.1432	7418.1398	0.0034	10	1	9	10	9	1	8	9
7418.2222	7418.2098	0.0124	10	1	9	9	9	1	8	8

Table S15: Observed and calculated rotational transitions (MHz) for the ^{13}C (atom number 2) isotopologue of the Z isomer

Observed	Calculated	Obs-Calc	J'	K_a'	K_c'	F'	J"	K_a"	K_c"	F"
2942.5721	2942.5655	0.0066	4	0	4	5	3	0	3	4
2948.7088	2948.7087	0.0001	4	2	2	5	3	2	1	4
2977.5997	2977.5969	0.0028	4	1	3	5	3	1	2	4
2977.6625	2977.6532	0.0093	4	1	3	4	3	1	2	3
2977.7367	2977.7320	0.0047	4	1	3	3	3	1	2	2
3640.1811	3640.1774	0.0037	5	1	5	4	4	1	4	3
3640.2125	3640.2124	0.0001	5	1	5	5	4	1	4	4

3640.2125	3640.2317	-0.0192	5	1	5	6	4	1	4	5
3675.7209	3675.7204	0.0005	5	0	5	5	4	0	4	4
3675.8253	3675.8377	-0.0124	5	0	5	4	4	0	4	3
3675.8253	3675.8091	0.0162	5	0	5	6	4	0	4	5
3681.4904	3681.4915	-0.0011	5	2	4	6	4	2	3	5
3681.4904	3681.4856	0.0048	5	2	4	4	4	2	3	3
3681.5625	3681.5565	0.0060	5	2	4	5	4	2	3	4
3687.9186	3687.9084	0.0102	5	2	3	6	4	2	2	5
3688.0762	3688.0675	0.0087	5	2	3	5	4	2	2	4
3721.4744	3721.4724	0.0020	5	1	4	4	4	1	3	3
4367.4145	4367.4073	0.0072	6	1	6	5	5	1	5	4
4367.4145	4367.4144	0.0001	6	1	6	6	5	1	5	5
4407.3875	4407.3921	-0.0046	6	0	6	6	5	0	5	5
4407.4827	4407.4999	-0.0172	6	0	6	7	5	0	5	6
4417.2247	4417.2101	0.0146	6	2	5	5	5	2	4	4
4417.2247	4417.2366	-0.0119	6	2	5	6	5	2	4	5
4428.3779	4428.3717	0.0062	6	2	4	5	5	2	3	4
4428.3779	4428.3793	-0.0014	6	2	4	7	5	2	3	6
4428.5200	4428.5200	-0.0000	6	2	4	6	5	2	3	5
4464.7322	4464.7239	0.0083	6	1	5	6	5	1	4	5
4464.7322	4464.7248	0.0074	6	1	5	7	5	1	4	6
5094.2052	5094.2087	-0.0035	7	1	7	7	6	1	6	6
5094.2052	5094.2211	-0.0159	7	1	7	6	6	1	6	5
5137.2976	5137.2865	0.0111	7	0	7	7	6	0	6	6
5137.4178	5137.4110	0.0068	7	0	7	8	6	0	6	7
5137.4178	5137.4292	-0.0114	7	0	7	6	6	0	6	5
5207.5258	5207.5449	-0.0191	7	1	6	6	6	1	5	5

5820.5348	5820.5219	0.0129	8	1	8	8	7	1	7	7
5820.5348	5820.5375	-0.0027	8	1	8	7	7	1	7	6
5887.6032	5887.6014	0.0018	8	2	7	8	7	2	6	7
5887.6032	5887.6110	-0.0078	8	2	7	7	7	2	6	6
5887.6032	5887.6061	-0.0029	8	2	7	9	7	2	6	8
5893.7910	5893.8039	-0.0129	8	4	5	9	7	4	4	8
5893.7910	5893.7946	-0.0036	8	4	5	7	7	4	4	6
5893.7910	5893.8082	-0.0172	8	4	4	7	7	4	3	6
5894.9465	5894.9356	0.0109	8	3	6	7	7	3	5	6
5894.9465	5894.9380	0.0085	8	3	6	9	7	3	5	8
5913.7661	5913.7573	0.0088	8	2	6	7	7	2	5	6
5913.7661	5913.7609	0.0052	8	2	6	9	7	2	5	8
5913.9029	5913.8961	0.0068	8	2	6	8	7	2	5	7
5949.5621	5949.5600	0.0021	8	1	7	8	7	1	6	7
5949.6249	5949.6224	0.0025	8	1	7	7	7	1	6	6
6546.3995	6546.3924	0.0071	9	1	9	8	8	1	8	7
6546.3995	6546.4054	-0.0059	9	1	9	10	8	1	8	9
6591.2522	6591.2555	-0.0033	9	0	9	9	8	0	8	8
6591.3969	6591.4150	-0.0181	9	0	9	8	8	0	8	7
6591.3969	6591.4021	-0.0052	9	0	9	10	8	0	8	9
6622.1967	6622.1904	0.0063	9	2	8	8	8	2	7	7
6622.1967	6622.1857	0.0110	9	2	8	10	8	2	7	9
6632.5075	6632.4978	0.0097	9	3	7	8	8	3	6	7
6632.5075	6632.4983	0.0092	9	3	7	10	8	3	6	9
6658.7579	6658.7521	0.0058	9	2	7	8	8	2	6	7
6658.7579	6658.7550	0.0029	9	2	7	10	8	2	6	9
6658.8875	6658.8891	-0.0016	9	2	7	9	8	2	6	8

6690.8348	6690.8536	-0.0188	9	1	8	10	8	1	7	9
7271.7252	7271.7369	-0.0117	10	1	10	11	9	1	9	10
7271.7252	7271.7269	-0.0017	10	1	10	9	9	1	9	8
7315.3603	7315.3619	-0.0016	10	0	10	10	9	0	9	9
7315.5331	7315.5127	0.0204	10	0	10	11	9	0	9	10
7315.5331	7315.5234	0.0097	10	0	10	9	9	0	9	8
7356.2001	7356.1934	0.0067	10	2	9	10	9	2	8	9
7356.2001	7356.2087	-0.0086	10	2	9	9	9	2	8	8
7370.2166	7370.1998	0.0168	10	3	8	9	9	3	7	8
7370.2166	7370.1993	0.0173	10	3	8	11	9	3	7	10
7370.2166	7370.2365	-0.0199	10	3	8	10	9	3	7	9
7405.1308	7405.1470	-0.0162	10	2	8	9	9	2	7	8
7405.1308	7405.1465	-0.0157	10	2	8	11	9	2	7	10
7405.2724	7405.2788	-0.0064	10	2	8	10	9	2	7	9

Table S16: Observed and calculated rotational transitions (MHz) for the ^{13}C (atom number 3) isotopologue of the Z isomer

Observed	Calculated	Obs-Calc	J'	K_a'	K_c'	F'	J"	K_a"	K_c"	F"
2186.8106	2186.8169	-0.0063	3	1	3	4	2	1	2	3
2210.6399	2210.6537	-0.0138	3	0	3	4	2	0	2	3
2236.7456	2236.7452	0.0004	3	1	2	4	2	1	1	3
2236.8815	2236.8803	0.0012	3	1	2	3	2	1	1	2
2915.3497	2915.3516	-0.0019	4	1	4	4	3	1	3	3
2915.3497	2915.3561	-0.0064	4	1	4	5	3	1	3	4
3643.5648	3643.5741	-0.0093	5	1	5	6	4	1	4	5
3643.5648	3643.5541	0.0107	5	1	5	5	4	1	4	4
3679.9165	3679.9110	0.0055	5	0	5	6	4	0	4	5

3685.9193	3685.9138	0.0055	5	2	4	4	4	2	3	3
3685.9193	3685.9196	-0.0003	5	2	4	6	4	2	3	5
3692.7103	3692.7041	0.0062	5	2	3	6	4	2	2	5
3692.8622	3692.8657	-0.0035	5	2	3	5	4	2	2	4
3726.8134	3726.8213	-0.0079	5	1	4	6	4	1	3	5
3726.9101	3726.9062	0.0039	5	1	4	4	4	1	3	3
4371.3763	4371.3786	-0.0023	6	1	6	6	5	1	5	5
4371.3763	4371.3724	0.0039	6	1	6	5	5	1	5	4
4422.4830	4422.4875	-0.0045	6	2	5	7	5	2	4	6
4422.4830	4422.4898	-0.0068	6	2	5	5	5	2	4	4
4434.2904	4434.2938	-0.0034	6	2	4	7	5	2	3	6
4434.2904	4434.2860	0.0044	6	2	4	5	5	2	3	4
4434.4323	4434.4371	-0.0048	6	2	4	6	5	2	3	5
4471.1865	4471.1882	-0.0017	6	1	5	7	5	1	4	6
4471.1865	4471.1862	0.0003	6	1	5	6	5	1	4	5
4471.2544	4471.2465	0.0079	6	1	5	5	5	1	4	4
5142.5490	5142.5461	0.0029	7	0	7	7	6	0	6	6
5142.6766	5142.6740	0.0026	7	0	7	8	6	0	6	7
5142.6766	5142.6925	-0.0159	7	0	7	6	6	0	6	5
5158.7151	5158.7091	0.0060	7	2	6	8	6	2	5	7
5158.7151	5158.7167	-0.0016	7	2	6	7	6	2	5	6
5158.7151	5158.7136	0.0015	7	2	6	6	6	2	5	5
5163.9336	5163.9303	0.0033	7	3	5	8	6	3	4	7
5163.9336	5163.9234	0.0102	7	3	5	6	6	3	4	5
5177.4028	5177.4047	-0.0019	7	2	5	8	6	2	4	7
5177.4028	5177.3998	0.0030	7	2	5	6	6	2	4	5
5177.5446	5177.5434	0.0012	7	2	5	7	6	2	4	6

5894.5334	5894.5301	0.0033	8	2	7	9	7	2	6	8
5894.5334	5894.5246	0.0088	8	2	7	8	7	2	6	7
5894.5334	5894.5351	-0.0017	8	2	7	7	7	2	6	6
5901.0703	5901.0760	-0.0057	8	4	5	7	7	4	4	6
5901.0703	5901.0854	-0.0151	8	4	5	9	7	4	4	8
5902.2601	5902.2665	-0.0064	8	3	6	7	7	3	5	6
5902.2601	5902.2689	-0.0088	8	3	6	9	7	3	5	8
5922.1247	5922.1273	-0.0026	8	2	6	9	7	2	5	8
5922.1247	5922.1237	0.0010	8	2	6	7	7	2	5	6
5922.2625	5922.2651	-0.0026	8	2	6	8	7	2	5	7
5957.9638	5957.9691	-0.0053	8	1	7	8	7	1	6	7
5958.0424	5958.0332	0.0092	8	1	7	7	7	1	6	6
6552.0924	6552.0899	0.0025	9	1	9	8	8	1	8	7
6552.0924	6552.1027	-0.0103	9	1	9	10	8	1	8	9
6597.3310	6597.3246	0.0064	9	0	9	9	8	0	8	8
6597.4812	6597.4872	-0.0060	9	0	9	8	8	0	8	7
6597.4812	6597.4743	0.0069	9	0	9	10	8	0	8	9
6629.8994	6629.9015	-0.0021	9	2	8	8	8	2	7	7
6629.8994	6629.8968	0.0026	9	2	8	10	8	2	7	9
6629.8994	6629.8818	0.0176	9	2	8	9	8	2	7	8
6638.2738	6638.2556	0.0182	9	5	4	8	8	5	3	7
6638.2738	6638.2652	0.0086	9	5	5	10	8	5	4	9
6638.2738	6638.2656	0.0082	9	5	4	10	8	5	3	9
6638.2738	6638.2552	0.0186	9	5	5	8	8	5	4	7
6639.3121	6639.3328	-0.0207	9	4	5	10	8	4	4	9
6639.3952	6639.3981	-0.0029	9	4	5	9	8	4	4	8
6642.7699	6642.7851	-0.0152	9	3	6	10	8	3	5	9

6642.7699	6642.7830	-0.0131	9	3	6	8	8	3	5	7
6642.8375	6642.8487	-0.0112	9	3	6	9	8	3	5	8
6668.4373	6668.4350	0.0023	9	2	7	10	8	2	6	9
6668.4373	6668.4321	0.0052	9	2	7	8	8	2	6	7
6668.5754	6668.5712	0.0042	9	2	7	9	8	2	6	8
6700.1346	6700.1217	0.0129	9	1	8	9	8	1	7	8
7277.9444	7277.9555	-0.0111	10	1	10	9	9	1	9	8
7277.9444	7277.9241	0.0203	10	1	10	10	9	1	9	9
7321.8965	7321.9028	-0.0063	10	0	10	9	9	0	9	8
7321.8965	7321.8921	0.0044	10	0	10	11	9	0	9	10
7416.1707	7416.1686	0.0021	10	2	8	11	9	2	7	10
7416.1707	7416.1686	0.0021	10	2	8	9	9	2	7	8
7441.2655	7441.2857	-0.0202	10	1	9	11	9	1	8	10

Table S17: Observed and calculated rotational transitions (MHz) for the ^{13}C (atom number 4) isotopologue of the Z isomer

Observed	Calculated	Obs-Calc	J'	K$_a$'	K$_c$'	F'	J"	K$_a$"	K$_c$"	F"
2207.0471	2207.0493	-0.0022	3	0	3	4	2	0	2	3
2912.1378	2912.1345	0.0033	4	1	4	4	3	1	3	3
2912.1378	2912.1382	-0.0004	4	1	4	5	3	1	3	4
2975.3576	2975.3674	-0.0098	4	1	3	5	3	1	2	4
2975.4250	2975.4240	0.0010	4	1	3	4	3	1	2	3
2975.5043	2975.5024	0.0019	4	1	3	3	3	1	2	2
3674.2425	3674.2379	0.0046	5	0	5	5	4	0	4	4
3674.3250	3674.3252	-0.0002	5	0	5	6	4	0	4	5
3674.3500	3674.3536	-0.0036	5	0	5	4	4	0	4	3
3679.7718	3679.7706	0.0012	5	2	4	6	4	2	3	5

3679.7718	3679.7646	0.0072	5	2	4	4	4	2	3	3
3685.9193	3685.9045	0.0148	5	2	3	4	4	2	2	3
3685.9193	3685.9206	-0.0013	5	2	3	6	4	2	2	5
3718.6168	3718.6277	-0.0109	5	1	4	6	4	1	3	5
3718.7178	3718.7124	0.0054	5	1	4	4	4	1	3	3
4366.7058	4366.7000	0.0058	6	1	6	6	5	1	5	5
4366.7058	4366.6925	0.0133	6	1	6	5	5	1	5	4
4405.7522	4405.7549	-0.0027	6	0	6	6	5	0	5	5
4405.8505	4405.8611	-0.0106	6	0	6	7	5	0	5	6
4425.8766	4425.8769	-0.0003	6	2	4	7	5	2	3	6
4425.8766	4425.8693	0.0073	6	2	4	5	5	2	3	4
4426.0097	4426.0162	-0.0065	6	2	4	6	5	2	3	5
4461.4462	4461.4531	-0.0069	6	1	5	6	5	1	4	5
4461.4462	4461.4536	-0.0074	6	1	5	7	5	1	4	6
4461.5125	4461.5116	0.0009	6	1	5	5	5	1	4	4
5093.4150	5093.4147	0.0003	7	1	7	7	6	1	6	6
5093.4150	5093.4243	-0.0093	7	1	7	6	6	1	6	5
5135.5746	5135.5640	0.0106	7	0	7	7	6	0	6	6
5135.6920	5135.6867	0.0053	7	0	7	8	6	0	6	7
5135.6920	5135.7048	-0.0128	7	0	7	6	6	0	6	5
5167.2305	5167.2209	0.0096	7	2	5	6	6	2	4	5
5167.2305	5167.2256	0.0049	7	2	5	8	6	2	4	7
5167.3676	5167.3600	0.0076	7	2	5	7	6	2	4	6
5203.7433	5203.7414	0.0019	7	1	6	8	6	1	5	7
5203.7433	5203.7273	0.0160	7	1	6	7	6	1	5	6
5819.6875	5819.6812	0.0063	8	1	8	8	7	1	7	7
5819.6875	5819.6985	-0.0110	8	1	8	7	7	1	7	6

5863.6564	5863.6545	0.0019	8	0	8	9	7	0	7	8
5863.6564	5863.6697	-0.0133	8	0	8	7	7	0	7	6
5910.0673	5910.0625	0.0048	8	2	6	9	7	2	5	8
5910.0673	5910.0590	0.0083	8	2	6	7	7	2	5	6
5910.1763	5910.1964	-0.0201	8	2	6	8	7	2	5	7
5945.3253	5945.3459	-0.0206	8	1	7	8	7	1	6	7
5945.3943	5945.4074	-0.0131	8	1	7	7	7	1	6	6
6545.5368	6545.5202	0.0166	9	1	9	10	8	1	8	9
6589.5812	6589.5701	0.0111	9	0	9	9	8	0	8	8
6589.7244	6589.7151	0.0093	9	0	9	10	8	0	8	9
6589.7244	6589.7280	-0.0036	9	0	9	8	8	0	8	7
6619.2917	6619.2761	0.0156	9	2	8	10	8	2	7	9
6619.2917	6619.2808	0.0109	9	2	8	8	8	2	7	7
6627.7899	6627.7972	-0.0073	9	4	6	10	8	4	5	9
6627.7899	6627.7936	-0.0037	9	4	6	8	8	4	5	7
6627.8596	6627.8628	-0.0032	9	4	6	9	8	4	5	8
6629.1724	6629.1719	0.0005	9	3	7	10	8	3	6	9
6629.1724	6629.1714	0.0010	9	3	7	8	8	3	6	7
6686.1867	6686.1800	0.0067	9	1	8	9	8	1	7	8
7270.7756	7270.7910	-0.0154	10	1	10	10	9	1	9	9
7270.8193	7270.8211	-0.0018	10	1	10	9	9	1	9	8
7270.8193	7270.8311	-0.0118	10	1	10	11	9	1	9	10
7353.1189	7353.1048	0.0141	10	2	9	10	9	2	8	9
7353.1189	7353.1225	-0.0036	10	2	9	11	9	2	8	10
7353.1189	7353.1310	-0.0121	10	2	9	9	9	2	8	8
7366.4794	7366.4795	-0.0001	10	3	8	9	9	3	7	8
7366.4794	7366.4791	0.0003	10	3	8	11	9	3	7	10

7366.5071	7366.5162	-0.0091	10	3	8	10	9	3	7	9
7426.0910	7426.0806	0.0104	10	1	9	10	9	1	8	9

Table S18: Observed and calculated rotational transitions (MHz) for the ^{13}C (atom number 9) isotopologue of the Z isomer

Observed	Calculated	Obs-Calc	J'	K_a'	K_c'	F'	J"	K_a"	K_c"	F"
2207.0488	2207.0437	0.0051	3	0	3	2	2	0	2	1
2947.4083	2947.4059	0.0024	4	2	2	4	3	2	1	3
2975.8625	2975.8616	0.0009	4	1	3	5	3	1	2	4
2975.9250	2975.9181	0.0069	4	1	3	4	3	1	2	3
2975.9875	2975.9967	-0.0092	4	1	3	3	3	1	2	2
3673.9899	3673.9915	-0.0016	5	0	5	5	4	0	4	4
3674.0738	3674.0795	-0.0057	5	0	5	6	4	0	4	5
3679.6593	3679.6607	-0.0014	5	2	4	4	4	2	3	3
3679.6593	3679.6666	-0.0073	5	2	4	6	4	2	3	5
3686.1203	3686.1348	-0.0145	5	2	3	5	4	2	2	4
3719.2133	3719.2295	-0.0162	5	1	4	6	4	1	3	5
4365.6743	4365.6933	-0.0190	6	1	6	7	5	1	5	6
4365.6743	4365.6663	0.0080	6	1	6	6	5	1	5	5
4365.6743	4365.6590	0.0153	6	1	6	5	5	1	5	4
4405.4615	4405.4812	-0.0197	6	0	6	7	5	0	5	6
4426.0091	4426.0064	0.0027	6	2	4	5	5	2	3	4
4426.0091	4426.0140	-0.0049	6	2	4	7	5	2	3	6
4426.1399	4426.1539	-0.0140	6	2	4	6	5	2	3	5
4462.1488	4462.1509	-0.0021	6	1	5	6	5	1	4	5
4462.1488	4462.1515	-0.0027	6	1	5	7	5	1	4	6
4462.2125	4462.2098	0.0027	6	1	5	5	5	1	4	4

5092.2069	5092.2113	-0.0044	7	1	7	8	6	1	6	7
5092.2069	5092.1989	0.0080	7	1	7	6	6	1	6	5
5135.1138	5135.1303	-0.0165	7	0	7	8	6	0	6	7
5150.0688	5150.0755	-0.0067	7	2	6	7	6	2	5	6
5150.0688	5150.0670	0.0018	7	2	6	8	6	2	5	7
5150.0688	5150.0714	-0.0026	7	2	6	6	6	2	5	5
5167.4813	5167.4734	0.0079	7	2	5	6	6	2	4	5
5167.4813	5167.4781	0.0032	7	2	5	8	6	2	4	7
5167.6159	5167.6133	0.0026	7	2	5	7	6	2	4	6
5204.5259	5204.5211	0.0048	7	1	6	8	6	1	5	7
5818.2394	5818.2335	0.0059	8	1	8	8	7	1	7	7
5818.2394	5818.2488	-0.0094	8	1	8	7	7	1	7	6
5862.7417	5862.7413	0.0004	8	0	8	8	7	0	7	7
5884.7342	5884.7288	0.0054	8	2	7	8	7	2	6	7
5884.7342	5884.7382	-0.0040	8	2	7	7	7	2	6	6
5884.7342	5884.7333	0.0009	8	2	7	9	7	2	6	8
5910.4623	5910.4647	-0.0024	8	2	6	9	7	2	5	8
5910.4623	5910.4612	0.0011	8	2	6	7	7	2	5	6
5910.6048	5910.5992	0.0056	8	2	6	8	7	2	5	7
5946.2427	5946.2511	-0.0084	8	1	7	7	7	1	6	6
6588.5322	6588.5310	0.0012	9	0	9	9	8	0	8	8
6588.6818	6588.6896	-0.0078	9	0	9	8	8	0	8	7
6588.6818	6588.6768	0.0050	9	0	9	10	8	0	8	9
6618.9826	6618.9766	0.0060	9	2	8	10	8	2	7	9
6618.9826	6618.9813	0.0013	9	2	8	8	8	2	7	7
6629.1724	6629.1691	0.0033	9	3	7	9	8	3	6	8
6630.9155	6630.9222	-0.0067	9	3	6	10	8	3	5	9

6630.9155	6630.9202	-0.0047	9	3	6	8	8	3	5	7
6630.9737	6630.9842	-0.0105	9	3	6	9	8	3	5	8
6654.9722	6654.9659	0.0063	9	2	7	10	8	2	6	9
6654.9722	6654.9631	0.0091	9	2	7	8	8	2	6	7
6687.0793	6687.0656	0.0137	9	1	8	9	8	1	7	8
7268.9352	7268.9354	-0.0002	10	1	10	11	9	1	9	10
7268.9352	7268.9254	0.0098	10	1	10	9	9	1	9	8
7312.4354	7312.4462	-0.0108	10	0	10	10	9	0	9	9
7312.6127	7312.6070	0.0057	10	0	10	9	9	0	9	8
7312.6127	7312.5963	0.0164	10	0	10	11	9	0	9	10
7426.9940	7426.9826	0.0114	10	1	9	10	9	1	8	9

Table S19: Observed and calculated rotational transitions (MHz) for the ^{13}C (atom number 10) isotopologue of the Z isomer

Observed	Calculated	Obs-Calc	J'	K_a'	K_c'	F'	J"	K_a"	K_c"	F"
2212.4711	2212.4562	0.0149	3	0	3	3	2	0	2	2
2951.4750	2951.4702	0.0048	4	2	3	5	3	2	2	4
2951.6133	2951.6125	0.0008	4	2	3	4	3	2	2	3
2954.7496	2954.7612	-0.0116	4	2	2	5	3	2	1	4
2954.9794	2954.9791	0.0003	4	2	2	4	3	2	1	3
2983.9198	2983.9227	-0.0029	4	1	3	5	3	1	2	4
2983.9875	2983.9788	0.0087	4	1	3	4	3	1	2	3
2984.0705	2984.0578	0.0127	4	1	3	3	3	1	2	2
3647.1751	3647.1671	0.0080	5	1	5	4	4	1	4	3
3647.2375	3647.2214	0.0161	5	1	5	6	4	1	4	5
3683.1263	3683.1281	-0.0018	5	0	5	6	4	0	4	5
3688.9458	3688.9490	-0.0032	5	2	4	6	4	2	3	5

3688.9458	3688.9432	0.0026	5	2	4	4	4	2	3	3
3695.6764	3695.6822	-0.0058	5	2	3	5	4	2	2	4
3729.2793	3729.2790	0.0003	5	1	4	6	4	1	3	5
3729.2793	3729.2993	-0.0200	5	1	4	5	4	1	3	4
4375.7921	4375.7822	0.0099	6	1	6	6	5	1	5	5
4375.7921	4375.8098	-0.0177	6	1	6	7	5	1	5	6
4375.7921	4375.7755	0.0166	6	1	6	5	5	1	5	4
4426.1397	4426.1424	-0.0027	6	2	5	7	5	2	4	6
4426.1397	4426.1447	-0.0050	6	2	5	5	5	2	4	4
4437.5783	4437.5759	0.0024	6	2	4	5	5	2	3	4
4437.5783	4437.5836	-0.0053	6	2	4	7	5	2	3	6
4474.1654	4474.1702	-0.0048	6	1	5	7	5	1	4	6
4474.1654	4474.1688	-0.0034	6	1	5	6	5	1	4	5
4474.2305	4474.2285	0.0020	6	1	5	5	5	1	4	4
5147.3375	5147.3276	0.0099	7	0	7	7	6	0	6	6
5147.4377	5147.4535	-0.0158	7	0	7	8	6	0	6	7
5162.9825	5163.0002	-0.0177	7	2	6	8	6	2	5	7
5162.9825	5163.0046	-0.0221	7	2	6	6	6	2	5	5
5181.1245	5181.1246	-0.0001	7	2	5	8	6	2	4	7
5181.1245	5181.1197	0.0048	7	2	5	6	6	2	4	5
5181.2582	5181.2616	-0.0034	7	2	5	7	6	2	4	6
5218.5369	5218.5298	0.0071	7	1	6	6	6	1	5	5
5831.6190	5831.6211	-0.0021	8	1	8	8	7	1	7	7
5831.6190	5831.6384	-0.0194	8	1	8	7	7	1	7	6
5831.6584	5831.6612	-0.0028	8	1	8	9	7	1	7	8
5876.6036	5876.6002	0.0034	8	0	8	8	7	0	7	7
5876.7511	5876.7393	0.0118	8	0	8	9	7	0	7	8

5876.7511	5876.7546	-0.0035	8	0	8	7	7	0	7	6
5899.4448	5899.4647	-0.0199	8	2	7	8	7	2	6	7
5905.8188	5905.8338	-0.0150	8	4	4	9	7	4	3	8
5905.8188	5905.8244	-0.0056	8	4	4	7	7	4	3	6
5905.8188	5905.8100	0.0088	8	4	5	7	7	4	4	6
5905.8188	5905.8194	-0.0006	8	4	5	9	7	4	4	8
5905.9289	5905.9216	0.0073	8	4	4	8	7	4	3	7
5908.0216	5908.0184	0.0032	8	3	5	7	7	3	4	6
5908.0216	5908.0221	-0.0005	8	3	5	9	7	3	4	8
5908.0995	5908.0926	0.0069	8	3	5	8	7	3	4	7
5926.2388	5926.2381	0.0007	8	2	6	9	7	2	5	8
5926.2388	5926.2345	0.0043	8	2	6	7	7	2	5	6
5926.3756	5926.3744	0.0012	8	2	6	8	7	2	5	7
5962.0563	5962.0654	-0.0091	8	1	7	8	7	1	6	7
5962.1269	5962.1285	-0.0016	8	1	7	7	7	1	6	6
6558.8109	6558.8121	-0.0012	9	1	9	9	8	1	8	8
6603.8588	6603.8696	-0.0108	9	0	9	9	8	0	8	8
6604.0303	6604.0175	0.0128	9	0	9	10	8	0	8	9
6604.0303	6604.0304	-0.0001	9	0	9	8	8	0	8	7
6635.4889	6635.4845	0.0044	9	2	8	9	8	2	7	8
6635.4889	6635.4990	-0.0101	9	2	8	10	8	2	7	9
6635.4889	6635.5038	-0.0149	9	2	8	8	8	2	7	7
6646.0639	6646.0521	0.0118	9	3	7	10	8	3	6	9
6646.0639	6646.0517	0.0122	9	3	7	8	8	3	6	7
6672.9057	6672.9065	-0.0008	9	2	7	10	8	2	6	9
6672.9057	6672.9036	0.0021	9	2	7	8	8	2	6	7
6673.0373	6673.0415	-0.0042	9	2	7	9	8	2	6	8

6704.8282	6704.8178	0.0104	9	1	8	9	8	1	7	8
7285.5186	7285.5220	-0.0034	10	1	10	11	9	1	9	10
7285.5186	7285.5121	0.0065	10	1	10	9	9	1	9	8
7329.2232	7329.2204	0.0028	10	0	10	10	9	0	9	9
7329.3826	7329.3723	0.0103	10	0	10	11	9	0	9	10
7329.3826	7329.3830	-0.0004	10	0	10	9	9	0	9	8
7385.2783	7385.2716	0.0067	10	3	8	9	9	3	7	8
7385.2783	7385.2712	0.0071	10	3	8	11	9	3	7	10
7420.9898	7420.9851	0.0047	10	2	8	11	9	2	7	10
7420.9898	7420.9852	0.0046	10	2	8	9	9	2	7	8

Table S20: Observed and calculated rotational transitions (MHz) for the ^{13}C (atom number 11) isotopologue of the Z isomer

Observed	Calculated	Obs-Calc	J'	K_a'	K_c'	F'	J"	K_a"	K_c"	F"
2189.6387	2189.6241	0.0146	3	1	3	4	2	1	2	3
2238.3072	2238.3128	-0.0056	3	1	2	4	2	1	1	3
2238.4416	2238.4482	-0.0066	3	1	2	3	2	1	1	2
2919.1145	2919.1194	-0.0049	4	1	4	5	3	1	3	4
2919.1145	2919.1154	-0.0009	4	1	4	4	3	1	3	3
2948.9623	2948.9737	-0.0114	4	0	4	4	3	0	3	3
2951.9632	2951.9729	-0.0097	4	2	3	5	3	2	2	4
2952.1014	2952.1153	-0.0139	4	2	3	4	3	2	2	3
2952.1014	2952.1149	-0.0135	4	2	3	4	3	2	2	4
2955.1713	2955.1889	-0.0176	4	2	2	5	3	2	1	4
2984.0696	2984.0860	-0.0164	4	1	3	5	3	1	2	4
2984.1416	2984.1423	-0.0007	4	1	3	4	3	1	2	3
2984.2394	2984.2211	0.0183	4	1	3	3	3	1	2	2

3648.3197	3648.3102	0.0095	5	1	5	6	4	1	4	5
3683.8045	3683.8094	-0.0049	5	0	5	5	4	0	4	4
3683.8981	3683.8982	-0.0001	5	0	5	6	4	0	4	5
3689.5841	3689.5868	-0.0027	5	2	4	6	4	2	3	5
3689.5841	3689.5809	0.0032	5	2	4	4	4	2	3	3
3696.0161	3696.0108	0.0053	5	2	3	6	4	2	2	5
4377.1186	4377.1076	0.0110	6	1	6	6	5	1	5	5
4377.1186	4377.1349	-0.0163	6	1	6	7	5	1	5	6
4377.1186	4377.1006	0.0180	6	1	6	5	5	1	5	4
4426.9215	4426.9238	-0.0023	6	2	5	5	5	2	4	4
4426.9215	4426.9216	-0.0001	6	2	5	7	5	2	4	6
4438.1000	4438.1054	-0.0054	6	2	4	7	5	2	3	6
4438.1000	4438.0977	0.0023	6	2	4	5	5	2	3	4
4438.2481	4438.2461	0.0020	6	2	4	6	5	2	3	5
4474.4567	4474.4565	0.0002	6	1	5	7	5	1	4	6
4474.4567	4474.4556	0.0011	6	1	5	6	5	1	4	5
4474.5219	4474.5148	0.0071	6	1	5	5	5	1	4	4
5105.5033	5105.5138	-0.0105	7	1	7	7	6	1	6	6
5163.9336	5163.9366	-0.0030	7	2	6	7	6	2	5	6
5163.9336	5163.9327	0.0009	7	2	6	6	6	2	5	5
5163.9336	5163.9283	0.0053	7	2	6	8	6	2	5	7
5181.6432	5181.6446	-0.0014	7	2	5	6	6	2	4	5
5181.6432	5181.6494	-0.0062	7	2	5	8	6	2	4	7
5181.7848	5181.7854	-0.0006	7	2	5	7	6	2	4	6
5833.4487	5833.4627	-0.0140	8	1	8	7	7	1	7	6
5833.4487	5833.4461	0.0026	8	1	8	8	7	1	7	7
5907.8920	5907.8953	-0.0033	8	3	6	9	7	3	5	8

5907.8920	5907.8929	-0.0009	8	3	6	7	7	3	5	6
5907.9639	5907.9539	0.0100	8	3	6	8	7	3	5	7
5908.9821	5908.9764	0.0057	8	3	5	8	7	3	4	7
5926.7358	5926.7345	0.0013	8	2	6	7	7	2	5	6
5926.7358	5926.7381	-0.0023	8	2	6	9	7	2	5	8
5926.8767	5926.8733	0.0034	8	2	6	8	7	2	5	7
5962.5459	5962.5315	0.0144	8	1	7	8	7	1	6	7
5962.5459	5962.5601	-0.0142	8	1	7	9	7	1	6	8
5962.5931	5962.5939	-0.0008	8	1	7	7	7	1	6	6
6560.9257	6560.9405	-0.0148	9	1	9	10	8	1	8	9
6560.9257	6560.9276	-0.0019	9	1	9	8	8	1	8	7
6605.7971	6605.7906	0.0065	9	0	9	9	8	0	8	8
6605.9199	6605.9373	-0.0174	9	0	9	10	8	0	8	9
6647.0869	6647.0758	0.0111	9	3	7	10	8	3	6	9
6647.0869	6647.0753	0.0116	9	3	7	8	8	3	6	7
6648.9367	6648.9219	0.0148	9	3	6	8	8	3	5	7
6648.9367	6648.9238	0.0129	9	3	6	10	8	3	5	9
6673.3535	6673.3565	-0.0030	9	2	7	8	8	2	6	7
6673.3535	6673.3594	-0.0059	9	2	7	10	8	2	6	9
6705.4252	6705.4436	-0.0184	9	1	8	10	8	1	7	9
7287.8375	7287.8445	-0.0070	10	1	10	10	9	1	9	9
7287.8836	7287.8851	-0.0015	10	1	10	11	9	1	9	10
7287.8836	7287.8751	0.0085	10	1	10	9	9	1	9	8
7331.5125	7331.5053	0.0072	10	0	10	10	9	0	9	9
7331.6688	7331.6561	0.0127	10	0	10	11	9	0	9	10
7331.6688	7331.6668	0.0020	10	0	10	9	9	0	9	8
7421.3828	7421.3787	0.0041	10	2	8	9	9	2	7	8

7421.3828	7421.3785	0.0043	10	2	8	11	9	2	7	10
7421.5056	7421.5106	-0.0050	10	2	8	10	9	2	7	9

Table S21: Observed and calculated rotational transitions (MHz) for the ^{13}C (atom number 13) isotopologue of the Z isomer

Observed	Calculated	Obs-Calc	J'	K_a'	K_c'	F'	J"	K_a"	K_c"	F"
2194.5213	2194.5409	-0.0196	3	0	3	4	2	0	2	3
2894.5625	2894.5574	0.0051	4	1	4	3	3	1	3	2
2894.6590	2894.6534	0.0056	4	1	4	4	3	1	3	3
2894.6590	2894.6573	0.0017	4	1	4	5	3	1	3	4
2924.4918	2924.4947	-0.0029	4	0	4	4	3	0	3	3
2927.4600	2927.4511	0.0089	4	2	3	3	3	2	2	2
2927.6248	2927.6307	-0.0059	4	2	3	4	3	2	2	3
2930.6850	2930.6981	-0.0131	4	2	2	5	3	2	1	4
2959.5681	2959.5803	-0.0122	4	1	3	5	3	1	2	4
2959.6401	2959.6366	0.0035	4	1	3	4	3	1	2	3
2959.6981	2959.7153	-0.0172	4	1	3	3	3	1	2	2
3653.2082	3653.2152	-0.0070	5	0	5	5	4	0	4	4
3653.3015	3653.3039	-0.0024	5	0	5	6	4	0	4	5
3658.9795	3658.9758	0.0037	5	2	4	4	4	2	3	3
3658.9795	3658.9817	-0.0022	5	2	4	6	4	2	3	5
3659.0500	3659.0468	0.0032	5	2	4	5	4	2	3	4
4380.3877	4380.3885	-0.0008	6	0	6	6	5	0	5	5
4380.4860	4380.4963	-0.0103	6	0	6	7	5	0	5	6
4390.2003	4390.1966	0.0037	6	2	5	7	5	2	4	6
4390.2003	4390.1989	0.0014	6	2	5	5	5	2	4	4
4437.7026	4437.7004	0.0022	6	1	5	6	5	1	4	5

4437.7026	4437.7013	0.0013	6	1	5	7	5	1	4	6
5062.6831	5062.6793	0.0038	7	1	7	6	6	1	6	5
5062.6831	5062.6856	-0.0025	7	1	7	7	6	1	6	6
5105.7875	5105.7858	0.0017	7	0	7	7	6	0	6	6
5105.9150	5105.9284	-0.0134	7	0	7	6	6	0	6	5
5105.9150	5105.9102	0.0048	7	0	7	8	6	0	6	7
5121.0907	5121.0924	-0.0017	7	2	6	7	6	2	5	6
5121.0907	5121.0840	0.0067	7	2	6	8	6	2	5	7
5121.0907	5121.0885	0.0022	7	2	6	6	6	2	5	5
5138.7705	5138.7724	-0.0019	7	2	5	8	6	2	4	7
5138.7705	5138.7676	0.0029	7	2	5	6	6	2	4	5
5138.9111	5138.9082	0.0029	7	2	5	7	6	2	4	6
5175.9852	5175.9732	0.0120	7	1	6	8	6	1	5	7
5784.5532	5784.5705	-0.0173	8	1	8	9	7	1	7	8
5784.5532	5784.5551	-0.0019	8	1	8	8	7	1	7	7
5829.2571	5829.2590	-0.0019	8	0	8	8	7	0	7	7
5829.4025	5829.3966	0.0059	8	0	8	9	7	0	7	8
5829.4025	5829.4119	-0.0094	8	0	8	7	7	0	7	6
5851.5927	5851.5976	-0.0049	8	2	7	7	7	2	6	6
5851.5927	5851.5880	0.0047	8	2	7	8	7	2	6	7
5851.5927	5851.5927	-0.0000	8	2	7	9	7	2	6	8
5857.7947	5857.7899	0.0048	8	4	4	7	7	4	3	6
5857.7947	5857.7856	0.0091	8	4	5	9	7	4	4	8
5857.7947	5857.7992	-0.0045	8	4	4	9	7	4	3	8
5857.8686	5857.8730	-0.0044	8	4	5	8	7	4	4	7
5857.8686	5857.8869	-0.0183	8	4	4	8	7	4	3	7
5858.9297	5858.9167	0.0130	8	3	6	7	7	3	5	6

5858.9297	5858.9191	0.0106	8	3	6	9	7	3	5	8
5859.9230	5859.9233	-0.0003	8	3	5	7	7	3	4	6
5859.9230	5859.9270	-0.0040	8	3	5	9	7	3	4	8
5859.9793	5859.9970	-0.0177	8	3	5	8	7	3	4	7
5877.7320	5877.7277	0.0043	8	2	6	9	7	2	5	8
5877.7320	5877.7242	0.0078	8	2	6	7	7	2	5	6
6550.9255	6550.9105	0.0150	9	0	9	10	8	0	8	9
6550.9255	6550.9233	0.0022	9	0	9	8	8	0	8	7
6581.6697	6581.6764	-0.0067	9	2	8	8	8	2	7	7
6581.6697	6581.6717	-0.0020	9	2	8	10	8	2	7	9
6581.6697	6581.6576	0.0121	9	2	8	9	8	2	7	8
6591.9818	6591.9766	0.0052	9	3	7	10	8	3	6	9
6591.9818	6591.9761	0.0057	9	3	7	8	8	3	6	7
6593.8161	6593.8170	-0.0009	9	3	6	8	8	3	5	7
6593.8161	6593.8189	-0.0028	9	3	6	10	8	3	5	9
6593.8625	6593.8812	-0.0187	9	3	6	9	8	3	5	8
6618.2143	6618.2141	0.0002	9	2	7	10	8	2	6	9
6618.2143	6618.2113	0.0030	9	2	7	8	8	2	6	7
6618.3469	6618.3482	-0.0013	9	2	7	9	8	2	6	8
7226.7592	7226.7469	0.0123	10	1	10	11	9	1	9	10
7360.1021	7360.0989	0.0032	10	2	8	9	9	2	7	8
7360.1021	7360.0977	0.0044	10	2	8	11	9	2	7	10
7360.2281	7360.2304	-0.0023	10	2	8	10	9	2	7	9

Table S22: Observed and calculated rotational transitions (MHz) for the ^{13}C (atom number 17) isotopologue of the Z isomer

Observed	Calculated	Obs-Calc	J'	K_a'	K_c'	F'	J"	K_a"	K_c"	F"

2205.9805	2205.9952	-0.0147	3	0	3	4	2	0	2	3
2942.5710	2942.5572	0.0138	4	2	3	5	3	2	2	4
2942.6877	2942.7002	-0.0125	4	2	3	4	3	2	2	3
2942.6932	2942.6998	-0.0066	4	2	3	4	3	2	2	4
2945.2596	2945.2740	-0.0144	4	2	2	3	3	2	1	2
2945.3132	2945.3211	-0.0079	4	2	2	5	3	2	1	4
2972.1889	2972.1944	-0.0055	4	1	3	5	3	1	2	4
2972.2530	2972.2519	0.0011	4	1	3	4	3	1	2	3
3639.8126	3639.7999	0.0127	5	1	5	4	4	1	4	3
3672.9008	3672.9013	-0.0005	5	0	5	5	4	0	4	4
3672.9774	3672.9839	-0.0065	5	0	5	6	4	0	4	5
3677.8674	3677.8680	-0.0006	5	2	4	4	4	2	3	3
3677.8674	3677.8741	-0.0067	5	2	4	6	4	2	3	5
3677.9407	3677.9401	0.0006	5	2	4	5	4	2	3	4
3714.7068	3714.7246	-0.0178	5	1	4	6	4	1	3	5
3714.8106	3714.8091	0.0015	5	1	4	4	4	1	3	3
4367.0780	4367.0667	0.0113	6	1	6	5	5	1	5	4
4367.0780	4367.0756	0.0024	6	1	6	6	5	1	5	5
4422.5752	4422.5698	0.0054	6	2	4	5	5	2	3	4
4422.5752	4422.5770	-0.0018	6	2	4	7	5	2	3	6
4422.7122	4422.7118	0.0004	6	2	4	6	5	2	3	5
4456.8669	4456.8671	-0.0002	6	1	5	6	5	1	4	5
4456.8669	4456.8658	0.0011	6	1	5	7	5	1	4	6
4456.9278	4456.9237	0.0041	6	1	5	5	5	1	4	4
5093.9521	5093.9641	-0.0120	7	1	7	6	6	1	6	5
5093.9521	5093.9561	-0.0040	7	1	7	7	6	1	6	6

5134.5172	5134.5166	0.0006	7	0	7	7	6	0	6	6
5134.6241	5134.6335	-0.0094	7	0	7	8	6	0	6	7
5147.7489	5147.7468	0.0021	7	2	6	6	6	2	5	5
5147.7489	5147.7523	-0.0034	7	2	6	7	6	2	5	6
5147.7489	5147.7425	0.0064	7	2	6	8	6	2	5	7
5163.1327	5163.1485	-0.0158	7	2	5	7	6	2	4	6
5198.5263	5198.5147	0.0116	7	1	6	7	6	1	5	6
5198.5263	5198.5267	-0.0004	7	1	6	8	6	1	5	7
5820.4083	5820.4298	-0.0215	8	1	8	8	7	1	7	7
5882.2057	5882.2133	-0.0076	8	2	7	7	7	2	6	6
5882.2057	5882.2055	0.0002	8	2	7	8	7	2	6	7
5882.2057	5882.2085	-0.0028	8	2	7	9	7	2	6	8
5904.8268	5904.8202	0.0066	8	2	6	7	7	2	5	6
5904.8268	5904.8235	0.0033	8	2	6	9	7	2	5	8
5904.9633	5904.9529	0.0104	8	2	6	8	7	2	5	7
5939.6219	5939.6372	-0.0153	8	1	7	7	7	1	6	6
6546.5185	6546.5022	0.0163	9	1	9	8	8	1	8	7
6546.5185	6546.5152	0.0033	9	1	9	10	8	1	8	9
6616.2993	6616.3036	-0.0043	9	2	8	10	8	2	7	9
6616.2993	6616.3082	-0.0089	9	2	8	8	8	2	7	7
6616.2993	6616.2914	0.0079	9	2	8	9	8	2	7	8
6679.9502	6679.9455	0.0047	9	1	8	9	8	1	7	8
7272.1111	7272.1155	-0.0044	10	1	10	11	9	1	9	10
7272.1111	7272.1054	0.0057	10	1	10	9	9	1	9	8
7314.4624	7314.4682	-0.0058	10	0	10	9	9	0	9	8
7314.4624	7314.4576	0.0048	10	0	10	11	9	0	9	10
7362.0472	7362.0438	0.0034	10	3	8	11	9	3	7	10

7362.0472	7362.0442	0.0030	10	3	8	9	9	3	7	8
7364.5905	7364.5755	0.0150	10	3	7	11	9	3	6	10
7364.5905	7364.5744	0.0161	10	3	7	9	9	3	6	8
7392.4727	7392.4836	-0.0109	10	2	8	9	9	2	7	8
7392.4727	7392.4838	-0.0111	10	2	8	11	9	2	7	10
7392.6163	7392.6120	0.0043	10	2	8	10	9	2	7	9
7419.4982	7419.4847	0.0135	10	1	9	10	9	1	8	9

Table S23: Observed and calculated rotational transitions (MHz) for the ^{13}C (atom number 21) isotopologue of the Z isomer

Observed	Calculated	Obs-Calc	J'	K_a'	K_c'	F'	J"	K_a"	K_c"	F"
2199.6413	2199.6406	0.0007	3	0	3	3	2	0	2	2
2199.6849	2199.6863	-0.0014	3	0	3	4	2	0	2	3
2900.0728	2900.0724	0.0004	4	1	4	3	3	1	3	2
2900.1696	2900.1673	0.0023	4	1	4	4	3	1	3	3
2900.1696	2900.1721	-0.0025	4	1	4	5	3	1	3	4
2931.2809	2931.2710	0.0099	4	0	4	5	3	0	3	4
2934.4815	2934.4947	-0.0132	4	2	3	5	3	2	2	4
2934.6344	2934.6367	-0.0023	4	2	3	4	3	2	2	3
3624.5212	3624.5166	0.0046	5	1	5	4	4	1	4	3
3624.5781	3624.5708	0.0073	5	1	5	6	4	1	4	5
3661.4619	3661.4568	0.0051	5	0	5	6	4	0	4	5
3667.6947	3667.6937	0.0010	5	2	4	4	4	2	3	3
3667.6947	3667.6995	-0.0048	5	2	4	6	4	2	3	5
3667.7708	3667.7638	0.0070	5	2	4	5	4	2	3	4
3674.7534	3674.7478	0.0056	5	2	3	6	4	2	2	5
3674.9060	3674.9110	-0.0050	5	2	3	5	4	2	2	4

3709.3137	3709.3267	-0.0130	5	1	4	6	4	1	3	5
3709.4164	3709.4116	0.0048	5	1	4	4	4	1	3	3
4348.5359	4348.5413	-0.0054	6	1	6	6	5	1	5	5
4348.5359	4348.5356	0.0003	6	1	6	5	5	1	5	4
4389.8300	4389.8310	-0.0010	6	0	6	6	5	0	5	5
4389.9310	4389.9443	-0.0133	6	0	6	7	5	0	5	6
4400.5941	4400.5989	-0.0048	6	2	5	7	5	2	4	6
4400.5941	4400.6012	-0.0071	6	2	5	5	5	2	4	4
4412.8480	4412.8605	-0.0125	6	2	4	7	5	2	3	6
4412.8480	4412.8526	-0.0046	6	2	4	5	5	2	3	4
4450.1516	4450.1537	-0.0021	6	1	5	7	5	1	4	6
4450.1516	4450.1509	0.0007	6	1	5	6	5	1	4	5
4450.2177	4450.2120	0.0057	6	1	5	5	5	1	4	4
5116.3641	5116.3645	-0.0004	7	0	7	7	6	0	6	6
5116.4881	5116.4947	-0.0066	7	0	7	8	6	0	6	7
5116.5125	5116.5133	-0.0008	7	0	7	6	6	0	6	5
5133.1450	5133.1431	0.0019	7	2	6	6	6	2	5	5
5133.1450	5133.1385	0.0065	7	2	6	8	6	2	5	7
5133.1450	5133.1457	-0.0007	7	2	6	7	6	2	5	6
5152.5408	5152.5414	-0.0006	7	2	5	6	6	2	4	5
5152.5408	5152.5465	-0.0057	7	2	5	8	6	2	4	7
5190.3371	5190.3419	-0.0048	7	1	6	7	6	1	5	6
5190.3847	5190.4044	-0.0197	7	1	6	6	6	1	5	5
5795.1404	5795.1310	0.0094	8	1	8	7	7	1	7	6
5795.1404	5795.1562	-0.0158	8	1	8	9	7	1	7	8
5840.8234	5840.8184	0.0050	8	0	8	8	7	0	7	7
5840.9676	5840.9617	0.0059	8	0	8	9	7	0	7	8

5840.9676	5840.9773	-0.0097	8	0	8	7	7	0	7	6
5865.2785	5865.2674	0.0111	8	2	7	7	7	2	6	6
5865.2785	5865.2623	0.0162	8	2	7	9	7	2	6	8
5929.7809	5929.7728	0.0081	8	1	7	8	7	1	6	7
5929.8365	5929.8380	-0.0015	8	1	7	7	7	1	6	6
6517.6201	6517.6285	-0.0084	9	1	9	9	8	1	8	8
6517.6632	6517.6557	0.0075	9	1	9	8	8	1	8	7
6517.6632	6517.6686	-0.0054	9	1	9	10	8	1	8	9
6563.1643	6563.1690	-0.0047	9	0	9	9	8	0	8	8
6563.3232	6563.3208	0.0024	9	0	9	10	8	0	8	9
6563.3232	6563.3338	-0.0106	9	0	9	8	8	0	8	7
6596.9170	6596.8986	0.0184	9	2	8	9	8	2	7	8
6596.9170	6596.9142	0.0028	9	2	8	10	8	2	7	9
6596.9170	6596.9190	-0.0020	9	2	8	8	8	2	7	7
7239.6290	7239.6327	-0.0037	10	1	10	11	9	1	9	10
7239.6290	7239.6228	0.0062	10	1	10	9	9	1	9	8
7283.6927	7283.6946	-0.0019	10	0	10	9	9	0	9	8
7283.6927	7283.6839	0.0088	10	0	10	11	9	0	9	10
7381.2743	7381.2727	0.0016	10	2	8	11	9	2	7	10
7381.2743	7381.2729	0.0014	10	2	8	9	9	2	7	8
7381.4082	7381.4070	0.0012	10	2	8	10	9	2	7	9
7405.7295	7405.7217	0.0078	10	1	9	10	9	1	8	9

Table S24: Observed and calculated rotational transitions (MHz) for the ^{13}C (atom number 27) isotopologue of the Z isomer

Observed	Calculated	Obs-Calc	J'	K_a'	K_c'	F'	J"	K_a"	K_c"	F"
2200.5149	2200.5184	-0.0035	3	0	3	3	2	0	2	2

2200.6303	2200.6383	-0.0080	3	0	3	2	2	0	2	1
2225.5858	2225.5876	-0.0018	3	1	2	4	2	1	1	3
2225.7150	2225.7233	-0.0083	3	1	2	3	2	1	1	2
2903.0893	2903.0882	0.0011	4	1	4	4	3	1	3	3
2903.0893	2903.0918	-0.0025	4	1	4	5	3	1	3	4
2932.5831	2932.5672	0.0159	4	0	4	4	3	0	3	3
2932.6642	2932.6776	-0.0134	4	0	4	3	3	0	3	2
2935.4610	2935.4692	-0.0082	4	2	3	5	3	2	2	4
3628.2465	3628.2396	0.0069	5	1	5	4	4	1	4	3
3663.3792	3663.3779	0.0013	5	0	5	5	4	0	4	4
3663.4683	3663.4933	-0.0250	5	0	5	4	4	0	4	3
3663.4683	3663.4650	0.0033	5	0	5	6	4	0	4	5
3668.9641	3668.9643	-0.0002	5	2	4	4	4	2	3	3
3668.9641	3668.9703	-0.0062	5	2	4	6	4	2	3	5
3675.1825	3675.1877	-0.0052	5	2	3	6	4	2	2	5
3675.1826	3675.1717	0.0109	5	2	3	4	4	2	2	3
3675.3452	3675.3455	-0.0003	5	2	3	5	4	2	2	4
3708.3105	3708.3266	-0.0161	5	1	4	6	4	1	3	5
3708.4138	3708.4113	0.0025	5	1	4	4	4	1	3	3
4353.1029	4353.1069	-0.0040	6	1	6	5	5	1	5	4
4353.1029	4353.1145	-0.0116	6	1	6	6	5	1	5	5
4449.0810	4449.0821	-0.0011	6	1	5	6	5	1	4	5
4449.0810	4449.0824	-0.0014	6	1	5	7	5	1	4	6
4449.1492	4449.1407	0.0085	6	1	5	5	5	1	4	4
5135.1135	5135.1183	-0.0048	7	2	6	6	6	2	5	5
5135.1135	5135.1226	-0.0091	7	2	6	7	6	2	5	6
5135.1135	5135.1139	-0.0004	7	2	6	8	6	2	5	7

5152.4069	5152.4104	-0.0035	7	2	5	7	6	2	4	6
5189.2958	5189.2804	0.0154	7	1	6	7	6	1	5	6
5189.2958	5189.2945	0.0013	7	1	6	8	6	1	5	7
5189.3334	5189.3397	-0.0063	7	1	6	6	6	1	5	5
5845.9762	5845.9732	0.0030	8	0	8	8	7	0	7	7
5846.1238	5846.1240	-0.0002	8	0	8	7	7	0	7	6
5846.1238	5846.1089	0.0149	8	0	8	9	7	0	7	8
5867.6564	5867.6596	-0.0032	8	2	7	9	7	2	6	8
5867.6564	5867.6645	-0.0081	8	2	7	7	7	2	6	6
5867.6564	5867.6553	0.0011	8	2	7	8	7	2	6	7
5893.0319	5893.0264	0.0055	8	2	6	7	7	2	5	6
5893.0319	5893.0299	0.0020	8	2	6	9	7	2	5	8
5928.8048	5928.8164	-0.0116	8	1	7	8	7	1	6	7
5928.8770	5928.8778	-0.0008	8	1	7	7	7	1	6	6
6525.0500	6525.0529	-0.0029	9	1	9	9	8	1	8	8
6525.0860	6525.0910	-0.0050	9	1	9	10	8	1	8	9
6525.0890	6525.0780	0.0110	9	1	9	8	8	1	8	7
6569.7420	6569.7530	-0.0110	9	0	9	9	8	0	8	8
6569.8911	6569.8978	-0.0067	9	0	9	10	8	0	8	9
6569.8911	6569.9106	-0.0195	9	0	9	8	8	0	8	7
6599.7938	6599.7931	0.0007	9	2	8	8	8	2	7	7
6599.7938	6599.7884	0.0054	9	2	8	10	8	2	7	9
6599.7938	6599.7748	0.0190	9	2	8	9	8	2	7	8
6609.8054	6609.7937	0.0117	9	3	7	10	8	3	6	9
6609.8073	6609.7932	0.0141	9	3	7	8	8	3	6	7
6611.5387	6611.5480	-0.0093	9	3	6	8	8	3	5	7
6611.5387	6611.5499	-0.0112	9	3	6	10	8	3	5	9

6611.6093	6611.6115	-0.0022	9	3	6	9	8	3	5	8
6635.2827	6635.2828	-0.0001	9	2	7	8	8	2	6	7
6635.2827	6635.2856	-0.0029	9	2	7	10	8	2	6	9
6667.5582	6667.5597	-0.0015	9	1	8	9	8	1	7	8
7248.0720	7248.0697	0.0023	10	1	10	10	9	1	9	9
7248.1006	7248.1098	-0.0092	10	1	10	11	9	1	9	10
7248.1006	7248.0997	0.0009	10	1	10	9	9	1	9	8
7291.6643	7291.6718	-0.0075	10	0	10	10	9	0	9	9
7291.8568	7291.8318	0.0250	10	0	10	9	9	0	9	8
7331.4667	7331.4517	0.0150	10	2	9	9	9	2	8	8
7331.4667	7331.4516	0.0151	10	2	9	11	9	2	8	10

Table S25: Observed and calculated rotational transitions (MHz) for the ^{13}C (atom number 28) isotopologue of the Z isomer

Observed	Calculated	Obs-Calc	J'	K_a'	K_c'	F'	J"	K_a"	K_c"	F"
2221.9900	2221.9867	0.0033	3	1	2	4	2	1	1	3
2899.2349	2899.2383	-0.0034	4	1	4	3	3	1	3	2
2899.3227	2899.3382	-0.0155	4	1	4	5	3	1	3	4
2899.3227	2899.3348	-0.0121	4	1	4	4	3	1	3	3
2928.3649	2928.3631	0.0018	4	0	4	4	3	0	3	3
2928.4303	2928.4261	0.0042	4	0	4	5	3	0	3	4
2962.3422	2962.3409	0.0013	4	1	3	5	3	1	2	4
2962.4000	2962.3978	0.0022	4	1	3	4	3	1	2	3
2962.4756	2962.4759	-0.0003	4	1	3	3	3	1	2	2
3623.5612	3623.5625	-0.0013	5	1	5	4	4	1	4	3
3623.6229	3623.6170	0.0059	5	1	5	6	4	1	4	5
3658.1866	3658.1850	0.0016	5	0	5	5	4	0	4	4

3658.2592	3658.2711	-0.0119	5	0	5	6	4	0	4	5
3663.6217	3663.6217	0.0000	5	2	4	4	4	2	3	3
3663.6217	3663.6277	-0.0060	5	2	4	6	4	2	3	5
3663.7000	3663.6931	0.0069	5	2	4	5	4	2	3	4
3669.6862	3669.6776	0.0086	5	2	3	6	4	2	2	5
3669.8295	3669.8345	-0.0050	5	2	3	5	4	2	2	4
4347.5345	4347.5500	-0.0155	6	1	6	7	5	1	5	6
4347.5345	4347.5234	0.0111	6	1	6	6	5	1	5	5
4347.5345	4347.5156	0.0189	6	1	6	5	5	1	5	4
4386.5467	4386.5439	0.0028	6	0	6	6	5	0	5	5
4406.3321	4406.3421	-0.0100	6	2	4	7	5	2	3	6
4406.3321	4406.3346	-0.0025	6	2	4	5	5	2	3	4
4406.4726	4406.4803	-0.0077	6	2	4	6	5	2	3	5
4441.9357	4441.9419	-0.0062	6	1	5	7	5	1	4	6
4441.9357	4441.9420	-0.0063	6	1	5	6	5	1	4	5
5113.2249	5113.2192	0.0057	7	0	7	7	6	0	6	6
5113.3483	5113.3585	-0.0102	7	0	7	6	6	0	6	5
5113.3483	5113.3404	0.0079	7	0	7	8	6	0	6	7
5127.6776	5127.6737	0.0039	7	2	6	8	6	2	5	7
5127.6776	5127.6827	-0.0051	7	2	6	7	6	2	5	6
5127.6776	5127.6781	-0.0005	7	2	6	6	6	2	5	5
5131.5436	5131.5379	0.0057	7	4	3	8	6	4	2	7
5131.5436	5131.5335	0.0101	7	4	4	8	6	4	3	7
5131.6654	5131.6560	0.0094	7	4	4	7	6	4	3	6
5131.6654	5131.6605	0.0049	7	4	3	7	6	4	2	6
5132.3414	5132.3395	0.0019	7	3	5	8	6	3	4	7
5132.3414	5132.3327	0.0087	7	3	5	6	6	3	4	5

5132.4228	5132.4182	0.0046	7	3	5	7	6	3	4	6
5181.0124	5181.0006	0.0118	7	1	6	8	6	1	5	7
5794.1609	5794.1525	0.0084	8	1	8	7	7	1	7	6
5838.0711	5838.0631	0.0080	8	0	8	8	7	0	7	7
5838.2021	5838.2126	-0.0105	8	0	8	7	7	0	7	6
5838.2021	5838.1975	0.0046	8	0	8	9	7	0	7	8
5859.1832	5859.1806	0.0026	8	2	7	8	7	2	6	7
5859.1832	5859.1846	-0.0014	8	2	7	9	7	2	6	8
5859.1832	5859.1895	-0.0063	8	2	7	7	7	2	6	6
5883.8834	5883.8887	-0.0053	8	2	6	9	7	2	5	8
5883.8834	5883.8852	-0.0018	8	2	6	7	7	2	5	6
5884.0177	5884.0214	-0.0037	8	2	6	8	7	2	5	7
5919.4419	5919.4494	-0.0075	8	1	7	7	7	1	6	6
6516.8042	6516.8213	-0.0171	9	1	9	10	8	1	8	9
6516.8042	6516.7835	0.0207	9	1	9	9	8	1	8	8
6516.8042	6516.8083	-0.0041	9	1	9	8	8	1	8	7
6561.0136	6561.0226	-0.0090	9	0	9	9	8	0	8	8
6561.1567	6561.1663	-0.0096	9	0	9	10	8	0	8	9
6590.2876	6590.2898	-0.0022	9	2	8	10	8	2	7	9
6590.2876	6590.2765	0.0111	9	2	8	9	8	2	7	8
6590.2876	6590.2944	-0.0068	9	2	8	8	8	2	7	7
6624.8605	6624.8694	-0.0089	9	2	7	10	8	2	6	9
6624.8605	6624.8666	-0.0061	9	2	7	8	8	2	6	7
6624.9993	6625.0015	-0.0022	9	2	7	9	8	2	6	8
7238.9610	7238.9598	0.0012	10	1	10	9	9	1	9	8
7238.9610	7238.9699	-0.0089	10	1	10	11	9	1	9	10
7282.1480	7282.1554	-0.0074	10	0	10	10	9	0	9	9

7282.3086	7282.3038	0.0048	10	0	10	11	9	0	9	10
7282.3086	7282.3145	-0.0059	10	0	10	9	9	0	9	8
7320.9531	7320.9417	0.0114	10	2	9	11	9	2	8	10
7320.9531	7320.9365	0.0166	10	2	9	9	9	2	8	8
7336.9819	7336.9820	-0.0001	10	3	7	11	9	3	6	10
7336.9819	7336.9808	0.0011	10	3	7	9	9	3	6	8
7337.0453	7337.0405	0.0048	10	3	7	10	9	3	6	9

Table S26: Observed and calculated rotational transitions (MHz) for the ^{13}C (atom number 32) isotopologue of the Z isomer

Observed	Calculated	Obs-Calc	J'	K_a'	K_c'	F'	J"	K_a"	K_c"	F"
2949.8420	2949.8356	0.0064	4	1	3	3	3	1	2	2
3605.6765	3605.6803	-0.0038	5	1	5	4	4	1	4	3
3641.1120	3641.1016	0.0104	5	0	5	5	4	0	4	4
3641.1862	3641.1897	-0.0035	5	0	5	6	4	0	4	5
3646.7968	3646.8033	-0.0065	5	2	4	6	4	2	3	5
3646.7968	3646.7973	-0.0005	5	2	4	4	4	2	3	3
3646.8697	3646.8684	0.0013	5	2	4	5	4	2	3	4
3653.1454	3653.1266	0.0188	5	2	3	4	4	2	2	3
3653.1454	3653.1427	0.0027	5	2	3	6	4	2	2	5
3653.3015	3653.3013	0.0002	5	2	3	5	4	2	2	4
3686.5150	3686.5251	-0.0101	5	1	4	6	4	1	3	5
3686.6113	3686.6099	0.0014	5	1	4	4	4	1	3	3
4326.0388	4326.0206	0.0182	6	1	6	5	5	1	5	4
4326.0388	4326.0278	0.0110	6	1	6	6	5	1	5	5
4375.5864	4375.5914	-0.0050	6	2	5	5	5	2	4	4
4375.5864	4375.5892	-0.0028	6	2	5	7	5	2	4	6

4375.6125	4375.6181	-0.0056	6	2	5	6	5	2	4	5
4386.6222	4386.6270	-0.0048	6	2	4	7	5	2	3	6
4386.6222	4386.6194	0.0028	6	2	4	5	5	2	3	4
4386.7724	4386.7670	0.0054	6	2	4	6	5	2	3	5
4422.9001	4422.9013	-0.0012	6	1	5	6	5	1	4	5
4422.9001	4422.9019	-0.0018	6	1	5	7	5	1	4	6
5045.9161	5045.9312	-0.0151	7	1	7	6	6	1	6	5
5045.9161	5045.9252	-0.0091	7	1	7	7	6	1	6	6
5045.9625	5045.9613	0.0012	7	1	7	8	6	1	6	7
5088.9354	5088.9209	0.0145	7	0	7	7	6	0	6	6
5089.0397	5089.0445	-0.0048	7	0	7	8	6	0	6	7
5104.0595	5104.0557	0.0038	7	2	6	6	6	2	5	5
5104.0595	5104.0513	0.0082	7	2	6	8	6	2	5	7
5104.0595	5104.0598	-0.0003	7	2	6	7	6	2	5	6
5121.5424	5121.5394	0.0030	7	2	5	6	6	2	4	5
5121.5424	5121.5442	-0.0018	7	2	5	8	6	2	4	7
5121.6751	5121.6794	-0.0043	7	2	5	7	6	2	4	6
5765.3717	5765.3817	-0.0100	8	1	8	8	7	1	7	7
5765.4139	5765.4124	0.0015	8	1	8	9	7	1	7	8
5765.4139	5765.4030	0.0109	8	1	8	7	7	1	7	6
5810.0434	5810.0449	-0.0015	8	0	8	8	7	0	7	7
5810.1804	5810.1969	-0.0165	8	0	8	7	7	0	7	6
5810.1804	5810.1817	-0.0013	8	0	8	9	7	0	7	8
5832.1390	5832.1344	0.0046	8	2	7	8	7	2	6	7
5832.1390	5832.1389	0.0001	8	2	7	9	7	2	6	8
5832.1390	5832.1438	-0.0048	8	2	7	7	7	2	6	6
5838.2467	5838.2613	-0.0146	8	4	5	9	7	4	4	8

5838.2467	5838.2520	-0.0053	8	4	5	7	7	4	4	6
5838.2467	5838.2653	-0.0186	8	4	4	7	7	4	3	6
5838.3573	5838.3486	0.0087	8	4	5	8	7	4	4	7
5838.3573	5838.3622	-0.0049	8	4	4	8	7	4	3	7
5839.3878	5839.3854	0.0024	8	3	6	9	7	3	5	8
5839.3878	5839.3829	0.0049	8	3	6	7	7	3	5	6
5839.4497	5839.4439	0.0058	8	3	6	8	7	3	5	7
5840.3703	5840.3748	-0.0045	8	3	5	9	7	3	4	8
5840.3703	5840.3712	-0.0009	8	3	5	7	7	3	4	6
5840.4432	5840.4446	-0.0014	8	3	5	8	7	3	4	7
5857.9922	5857.9871	0.0051	8	2	6	7	7	2	5	6
5857.9922	5857.9906	0.0016	8	2	6	9	7	2	5	8
5858.1257	5858.1251	0.0006	8	2	6	8	7	2	5	7
6484.3715	6484.3646	0.0069	9	1	9	8	8	1	8	7
6484.3715	6484.3775	-0.0060	9	1	9	10	8	1	8	9
6529.2174	6529.2155	0.0019	9	0	9	9	8	0	8	8
6529.3662	6529.3614	0.0048	9	0	9	10	8	0	8	9
6529.3662	6529.3742	-0.0080	9	0	9	8	8	0	8	7
6559.7943	6559.8016	-0.0073	9	2	8	10	8	2	7	9
6559.7943	6559.7878	0.0065	9	2	8	9	8	2	7	8
6559.7943	6559.8063	-0.0120	9	2	8	8	8	2	7	7
6569.9936	6569.9951	-0.0015	9	3	7	8	8	3	6	7
6569.9936	6569.9956	-0.0020	9	3	7	10	8	3	6	9
6570.0500	6570.0414	0.0086	9	3	7	9	8	3	6	8
6571.8066	6571.8023	0.0043	9	3	6	8	8	3	5	7
6571.8066	6571.8042	0.0024	9	3	6	10	8	3	5	9
6571.8526	6571.8663	-0.0137	9	3	6	9	8	3	5	8

6595.9612	6595.9581	0.0031	9	2	7	10	8	2	6	9
6595.9612	6595.9553	0.0059	9	2	7	8	8	2	6	7
6628.1691	6628.1616	0.0075	9	1	8	9	8	1	7	8
7246.5061	7246.5039	0.0022	10	0	10	10	9	0	9	9
7246.6665	7246.6647	0.0018	10	0	10	9	9	0	9	8
7246.6665	7246.6540	0.0125	10	0	10	11	9	0	9	10
7335.3191	7335.3181	0.0010	10	2	8	11	9	2	7	10
7335.3191	7335.3215	-0.0024	10	2	8	9	9	2	7	8

Table S27: Observed and calculated rotational transitions (MHz) for the ^{13}C (atom number 36) isotopologue of the Z isomer

Observed	Calculated	Obs-Calc	J'	K_a'	K_c'	F'	J"	K_a"	K_c"	F"
2901.0454	2901.0387	0.0067	4	1	4	4	3	1	3	3
2901.0454	2901.0420	0.0034	4	1	4	5	3	1	3	4
2929.9068	2929.8990	0.0078	4	0	4	5	3	0	3	4
2963.5088	2963.5066	0.0022	4	1	3	5	3	1	2	4
2963.5702	2963.5635	0.0067	4	1	3	4	3	1	2	3
2963.6488	2963.6415	0.0073	4	1	3	3	3	1	2	2
3660.0624	3660.0621	0.0003	5	0	5	5	4	0	4	4
3660.1429	3660.1476	-0.0047	5	0	5	6	4	0	4	5
3671.3437	3671.3586	-0.0149	5	2	3	4	4	2	2	3
3671.5336	3671.5309	0.0027	5	2	3	5	4	2	2	4
3703.8131	3703.8215	-0.0084	5	1	4	6	4	1	3	5
3703.9109	3703.9061	0.0048	5	1	4	4	4	1	3	3
4350.0893	4350.0934	-0.0041	6	1	6	5	5	1	5	4
4350.0893	4350.1014	-0.0121	6	1	6	6	5	1	5	5
4350.1250	4350.1278	-0.0028	6	1	6	7	5	1	5	6

4388.8444	4388.8470	-0.0026	6	0	6	6	5	0	5	5
4388.9433	4388.9511	-0.0078	6	0	6	7	5	0	5	6
4397.9648	4397.9668	-0.0020	6	2	5	5	5	2	4	4
4397.9648	4397.9646	0.0002	6	2	5	7	5	2	4	6
4408.3321	4408.3367	-0.0046	6	2	4	7	5	2	3	6
4408.3321	4408.3294	0.0027	6	2	4	5	5	2	3	4
4408.4584	4408.4743	-0.0159	6	2	4	6	5	2	3	5
4443.7117	4443.7168	-0.0051	6	1	5	6	5	1	4	5
4443.7117	4443.7165	-0.0048	6	1	5	7	5	1	4	6
4443.7578	4443.7747	-0.0169	6	1	5	5	5	1	4	4
5115.9766	5115.9726	0.0040	7	0	7	7	6	0	6	6
5116.0881	5116.0931	-0.0050	7	0	7	8	6	0	6	7
5130.2063	5130.2094	-0.0031	7	2	6	6	6	2	5	5
5130.2063	5130.2050	0.0013	7	2	6	8	6	2	5	7
5130.2063	5130.2141	-0.0078	7	2	6	7	6	2	5	6
5146.6483	5146.6525	-0.0042	7	2	5	8	6	2	4	7
5146.6483	5146.6479	0.0004	7	2	5	6	6	2	4	5
5146.7764	5146.7852	-0.0088	7	2	5	7	6	2	4	6
5183.1007	5183.0918	0.0089	7	1	6	8	6	1	5	7
5797.6287	5797.6351	-0.0064	8	1	8	7	7	1	7	6
5797.6287	5797.6206	0.0081	8	1	8	8	7	1	7	7
5797.6513	5797.6622	-0.0109	8	1	8	9	7	1	7	8
5841.2964	5841.2918	0.0046	8	0	8	8	7	0	7	7
5841.4329	5841.4254	0.0075	8	0	8	9	7	0	7	8
5841.4329	5841.4405	-0.0076	8	0	8	7	7	0	7	6
5862.0986	5862.0897	0.0089	8	2	7	8	7	2	6	7
5862.0986	5862.0984	0.0002	8	2	7	7	7	2	6	6

5862.0986	5862.0935	0.0051	8	2	7	9	7	2	6	8
5886.4301	5886.4168	0.0133	8	2	6	7	7	2	5	6
5886.4301	5886.4202	0.0099	8	2	6	9	7	2	5	8
5921.8567	5921.8681	-0.0114	8	1	7	7	7	1	6	6
6520.7000	6520.7166	-0.0166	9	1	9	9	8	1	8	8
6520.7575	6520.7541	0.0034	9	1	9	10	8	1	8	9
6520.7575	6520.7411	0.0164	9	1	9	8	8	1	8	7
6564.7541	6564.7495	0.0046	9	0	9	9	8	0	8	8
6564.8941	6564.9052	-0.0111	9	0	9	8	8	0	8	7
6564.8941	6564.8924	0.0017	9	0	9	10	8	0	8	9
6593.5765	6593.5872	-0.0107	9	2	8	8	8	2	7	7
6593.5765	6593.5695	0.0070	9	2	8	9	8	2	7	8
6593.5765	6593.5826	-0.0061	9	2	8	10	8	2	7	9
6627.6398	6627.6407	-0.0009	9	2	7	8	8	2	6	7
6627.6398	6627.6435	-0.0037	9	2	7	10	8	2	6	9
6627.7866	6627.7750	0.0116	9	2	7	9	8	2	6	8
6659.7875	6659.7806	0.0069	9	1	8	9	8	1	7	8
7243.3295	7243.3275	0.0020	10	1	10	10	9	1	9	9
7243.3625	7243.3570	0.0055	10	1	10	9	9	1	9	8
7243.3625	7243.3670	-0.0045	10	1	10	11	9	1	9	10
7286.5599	7286.5467	0.0132	10	0	10	11	9	0	9	10
7286.5599	7286.5574	0.0025	10	0	10	9	9	0	9	8
7340.4087	7340.4011	0.0076	10	3	7	9	9	3	6	8
7340.4087	7340.4023	0.0064	10	3	7	11	9	3	6	10
7370.2157	7370.2161	-0.0004	10	2	8	9	9	2	7	8
7370.2157	7370.2154	0.0003	10	2	8	11	9	2	7	10

^{18}O Isotopologues of 1-w-Z_N, 1-w-Z_O, 1-w-E and 2-w-E

Table S28: Observed and calculated rotational transitions (MHz) for 1-w(^{18}O)-Z_N

Observed	Calculated	Obs-Calc	J'	K_a'	K_c'	F'	J"	K_a"	K_c"	F"
2063.4194	2063.4145	0.0049	3	1	2	2	2	1	1	1
2063.1748	2063.1862	-0.0114	3	1	2	3	2	1	1	2
2063.1748	2063.1674	0.0074	3	1	2	4	2	1	1	3
2541.0131	2541.0054	0.0077	4	0	4	3	3	0	3	2
2540.5949	2540.5918	0.0031	4	0	4	4	3	0	3	3
2540.9436	2540.9451	-0.0015	4	0	4	5	3	0	3	4
2737.3655	2737.3617	0.0038	4	1	3	3	3	1	2	2
2737.1352	2737.1298	0.0054	4	1	3	4	3	1	2	3
2737.2287	2737.2214	0.0073	4	1	3	5	3	1	2	4
2479.1502	2479.1657	-0.0155	4	1	4	3	3	1	3	2
2479.1502	2479.1501	0.0001	4	1	4	4	3	1	3	3
2479.2429	2479.2417	0.0012	4	1	4	5	3	1	3	4
5473.3055	5473.3089	-0.0034	9	1	9	10	8	1	8	9
2707.8251	2707.8403	-0.0152	4	2	2	4	3	2	1	3
2707.4437	2707.4480	-0.0043	4	2	2	5	3	2	1	4
5489.4039	5489.3940	0.0099	8	2	6	8	7	2	5	7
2620.4050	2620.3981	0.0069	4	2	3	5	3	2	2	4
3132.5991	3132.6170	-0.0179	5	0	5	4	4	0	4	3
3132.2949	3132.2792	0.0157	5	0	5	5	4	0	4	4
3132.5991	3132.5901	0.0090	5	0	5	6	4	0	4	5
3397.0028	3396.9968	0.0060	5	1	4	4	4	1	3	3
3396.7292	3396.7187	0.0105	5	1	4	5	4	1	3	4
3396.9072	3396.8984	0.0088	5	1	4	6	4	1	3	5

4889.3477	4889.3554	-0.0077	8	0	8	8	7	0	7	7
3085.9506	3085.9527	-0.0021	5	1	5	5	4	1	4	4
3086.0480	3086.0566	-0.0086	5	1	5	6	4	1	4	5
3415.2891	3415.2830	0.0061	5	2	3	4	4	2	2	3
3415.5516	3415.5549	-0.0033	5	2	3	5	4	2	2	4
3415.2891	3415.2873	0.0018	5	2	3	6	4	2	2	5
3263.3596	3263.3723	-0.0127	5	2	4	4	4	2	3	3
6359.2250	6359.2422	-0.0172	10	2	9	10	9	2	8	9
3263.3596	3263.3622	-0.0026	5	2	4	6	4	2	3	5
3328.5827	3328.5800	0.0027	5	3	2	4	4	3	1	3
3328.8960	3328.8977	-0.0017	5	3	2	5	4	3	1	4
3328.6299	3328.6279	0.0020	5	3	2	6	4	3	1	5
5449.1482	5449.1499	-0.0017	8	3	5	8	7	3	4	7
3310.3266	3310.3235	0.0031	5	3	3	5	4	3	2	4
3310.1783	3310.1658	0.0125	5	3	3	6	4	3	2	5
3717.6785	3717.6839	-0.0054	6	0	6	5	5	0	5	4
3717.4408	3717.4429	-0.0021	6	0	6	6	5	0	5	5
3717.6785	3717.6788	-0.0003	6	0	6	7	5	0	5	6
4036.6233	4036.6138	0.0095	6	1	5	5	5	1	4	4
4036.2874	4036.2767	0.0107	6	1	5	6	5	1	4	5
4036.5430	4036.5359	0.0071	6	1	5	7	5	1	4	6
5489.4910	5489.5040	-0.0130	8	2	6	7	7	2	5	6
3687.5830	3687.5827	0.0003	6	1	6	6	5	1	5	5
3687.6854	3687.6880	-0.0026	6	1	6	7	5	1	5	6
4120.5981	4120.6176	-0.0195	6	2	4	5	5	2	3	4
4120.7430	4120.7366	0.0064	6	2	4	6	5	2	3	5
4120.5981	4120.5984	-0.0003	6	2	4	7	5	2	3	6

3898.6984	3898.7094	-0.0110	6	2	5	5	5	2	4	4
3898.6129	3898.6117	0.0012	6	2	5	6	5	2	4	5
3898.6984	3898.6962	0.0022	6	2	5	7	5	2	4	6
3974.1957	3974.1849	0.0108	6	3	4	5	5	3	3	4
3974.2715	3974.2755	-0.0040	6	3	4	6	5	3	3	5
3974.1957	3974.1911	0.0046	6	3	4	7	5	3	3	6
6142.3509	6142.3654	-0.0145	9	2	7	8	8	2	6	7
3977.2943	3977.3066	-0.0123	6	4	2	6	5	4	1	5
3977.1167	3977.1120	0.0047	6	4	2	7	5	4	1	6
6359.3679	6359.3800	-0.0121	10	2	9	9	9	2	8	8
3974.7572	3974.7722	-0.0150	6	4	3	6	5	4	2	5
3974.5883	3974.5934	-0.0051	6	4	3	7	5	4	2	6
4302.6334	4302.6288	0.0046	7	0	7	6	6	0	6	5
4302.4672	4302.4657	0.0015	7	0	7	7	6	0	6	6
4302.6334	4302.6348	-0.0014	7	0	7	8	6	0	6	7
4644.9488	4644.9577	-0.0089	7	4	4	7	6	4	3	6
4285.2183	4285.2133	0.0050	7	1	7	7	6	1	6	6
4285.3233	4285.3105	0.0128	7	1	7	8	6	1	6	7
4652.5357	4652.5213	0.0144	7	1	6	6	6	1	5	5
4728.7428	4728.7444	-0.0016	7	3	4	7	6	3	3	6
4728.4938	4728.4755	0.0183	7	3	4	8	6	3	3	7
4634.8333	4634.8412	-0.0079	7	3	5	6	6	3	4	5
6065.4526	6065.4427	0.0099	10	1	10	9	9	1	9	8
4634.8333	4634.8381	-0.0048	7	3	5	8	6	3	4	7
4813.9875	4813.9945	-0.0070	7	2	5	8	6	2	4	7
5246.5514	5246.5360	0.0154	8	1	7	8	7	1	6	7
4814.0410	4814.0243	0.0167	7	2	5	6	6	2	4	5

4898.7105	4898.7097	0.0008	8	1	8	7	7	0	7	6
4898.5625	4898.5627	-0.0002	8	1	8	8	7	0	7	7
4898.7105	4898.7160	-0.0055	8	1	8	9	7	0	7	8
5289.7506	5289.7546	-0.0040	8	3	6	7	7	3	5	6
5289.7506	5289.7359	0.0147	8	3	6	8	7	3	5	7
5289.7506	5289.7453	0.0053	8	3	6	9	7	3	5	8
5337.9105	5337.9013	0.0092	8	4	4	7	7	4	3	6
5338.0770	5338.0860	-0.0090	8	4	4	8	7	4	3	7
5337.9105	5337.9159	-0.0054	8	4	4	9	7	4	3	8
4525.6779	4525.6753	0.0026	7	2	6	8	6	2	5	7
5828.0293	5828.0258	0.0035	9	1	8	9	8	1	7	8
5828.3291	5828.3186	0.0105	9	1	8	10	8	1	7	9
6067.8051	6067.7962	0.0089	10	0	10	9	9	0	9	8
6067.7293	6067.7351	-0.0058	10	0	10	10	9	0	9	9
6067.8051	6067.8072	-0.0021	10	0	10	11	9	0	9	10
4652.1573	4652.1429	0.0144	7	1	6	7	6	1	5	6
6065.3896	6065.3903	-0.0007	10	1	10	10	9	1	9	9
6065.4526	6065.4546	-0.0020	10	1	10	11	9	1	9	10
5478.0540	5478.0580	-0.0040	9	0	9	8	8	0	8	7
4020.7372	4020.7440	-0.0068	6	3	3	6	5	3	2	5
4052.8140	4052.8102	0.0038	19	7	13	20	19	6	14	20
4052.8140	4052.8253	-0.0113	19	7	13	19	19	6	14	19
4052.8140	4052.8092	0.0048	19	7	13	18	19	6	14	18
4266.7506	4266.7661	-0.0155	7	0	7	8	6	1	6	7
4266.7506	4266.7359	0.0147	7	0	7	7	6	1	6	6
4266.7506	4266.7366	0.0140	7	0	7	6	6	1	6	5
4336.9378	4336.9282	0.0096	15	1	14	15	15	0	15	15

4377.1106	4377.1060	0.0046	8	2	6	8	7	3	5	7
4525.5631	4525.5656	-0.0025	7	2	6	7	6	2	5	6
5489.4910	5489.4694	0.0216	8	2	6	9	7	2	5	8
4525.6779	4525.6900	-0.0121	7	2	6	6	6	2	5	5
4587.2878	4587.3006	-0.0128	19	4	16	19	19	3	17	19
4644.8239	4644.8278	-0.0039	7	4	4	8	6	4	3	7
4644.8239	4644.8135	0.0104	7	4	4	6	6	4	3	5
6142.1838	6142.1683	0.0155	9	2	7	9	8	2	6	8
6359.3679	6359.3722	-0.0043	10	2	9	11	9	2	8	10
4652.4653	4652.4581	0.0072	7	1	6	8	6	1	5	7
5316.6037	5316.6165	-0.0128	8	4	5	8	7	4	4	7
4653.1412	4653.1527	-0.0115	7	4	3	7	6	4	2	6
4652.9940	4652.9849	0.0091	7	4	3	8	6	4	2	7
4652.9502	4652.9656	-0.0154	7	4	3	6	6	4	2	5
6142.3509	6142.3291	0.0218	9	2	7	10	8	2	6	9
5477.9622	5477.9773	-0.0151	9	0	9	9	8	0	8	8
5478.0540	5478.0693	-0.0153	9	0	9	10	8	0	8	9
5144.2471	5144.2410	0.0061	8	2	7	9	7	2	6	8
5144.2471	5144.2529	-0.0058	8	2	7	7	7	2	6	6
4880.0815	4880.0852	-0.0037	8	1	8	8	7	1	7	7
4880.1697	4880.1716	-0.0019	8	1	8	9	7	1	7	8
4880.1697	4880.1556	0.0141	8	1	8	7	7	1	7	6
5987.9959	5988.0044	-0.0085	9	4	6	8	8	4	5	7
4889.4756	4889.4778	-0.0022	8	0	8	9	7	0	7	8
4889.4756	4889.4675	0.0081	8	0	8	7	7	0	7	6
5144.1097	5144.1155	-0.0058	8	2	7	8	7	2	6	7
5754.9257	5754.9259	-0.0002	9	2	8	9	8	2	7	8

5755.0599	5755.0573	0.0026	9	2	8	10	8	2	7	9
5755.0599	5755.0672	-0.0073	9	2	8	8	8	2	7	7
5316.5159	5316.5202	-0.0043	8	4	5	9	7	4	4	8
5987.9959	5988.0062	-0.0103	9	4	6	10	8	4	5	9
5473.2298	5473.2339	-0.0041	9	1	9	9	8	1	8	8
5448.9065	5448.9090	-0.0025	8	3	5	9	7	3	4	8
5448.9065	5448.8964	0.0101	8	3	5	7	7	3	4	6
5988.0730	5988.0734	-0.0004	9	4	6	9	8	4	5	8
4020.4713	4020.4785	-0.0072	6	3	3	7	5	3	2	6
3651.8172	3651.8194	-0.0022	6	0	6	7	5	1	5	6
5936.9345	5936.9499	-0.0154	9	3	7	10	8	3	6	9
5473.3055	5473.2952	0.0103	9	1	9	8	8	1	8	7
3913.9513	3913.9518	-0.0005	21	2	19	21	20	5	16	20
3753.1731	3753.1727	0.0004	6	1	6	6	5	0	5	5

Table S29: Observed and calculated rotational transitions (MHz) for 1-w(^{18}O)-Z_O

Observed	Calculated	Obs-Calc	J'	K_a'	K_c'	F'	J"	K_a"	K_c"	F"
3264.9514	3264.9460	0.0054	5	2	4	4	4	2	3	3
3264.9514	3264.9515	-0.0001	5	2	4	6	4	2	3	5
3265.0172	3265.0174	-0.0002	5	2	4	5	4	2	3	4
3392.0300	3392.0246	0.0054	5	1	4	5	4	1	3	4
3392.0300	3392.0456	-0.0156	5	1	4	6	4	1	3	5
3392.0662	3392.0712	-0.0050	5	1	4	4	4	1	3	3
3393.0166	3393.0248	-0.0082	5	3	2	6	4	3	1	5
3487.3147	3487.3062	0.0085	5	2	3	4	4	2	2	3
3487.3147	3487.3090	0.0057	5	2	3	6	4	2	2	5
3487.4042	3487.4004	0.0038	5	2	3	5	4	2	2	4

3645.2485	3645.2527	-0.0042	6	1	6	5	5	1	5	4
3645.2485	3645.2471	0.0014	6	1	6	6	5	1	5	5
3645.2485	3645.2457	0.0028	6	1	6	7	5	1	5	6
3656.9125	3656.9079	0.0046	6	0	6	5	5	0	5	4
3656.9125	3656.8865	0.0260	6	0	6	6	5	0	5	5
3656.9125	3656.8980	0.0145	6	0	6	7	5	0	5	6
3995.7361	3995.7448	-0.0087	6	1	5	6	5	1	4	5
3995.7625	3995.7827	-0.0202	6	1	5	7	5	1	4	6
3995.7875	3995.8023	-0.0148	6	1	5	5	5	1	4	4
4008.0829	4008.0695	0.0134	6	3	4	5	5	3	3	4
4008.0829	4008.0843	-0.0014	6	3	4	7	5	3	3	6
4008.1818	4008.1875	-0.0057	6	3	4	6	5	3	3	5
4185.2749	4185.2742	0.0007	6	2	4	5	5	2	3	4
4185.2749	4185.3024	-0.0275	6	2	4	6	5	2	3	5
4185.2749	4185.2667	0.0082	6	2	4	7	5	2	3	6
4231.8523	4231.8510	0.0013	7	1	7	6	6	1	6	5
4231.8523	4231.8448	0.0075	7	1	7	7	6	1	6	6
4231.8523	4231.8457	0.0066	7	1	7	8	6	1	6	7
4236.9155	4236.9273	-0.0118	7	0	7	6	6	0	6	5
4236.9155	4236.9153	0.0002	7	0	7	7	6	0	6	6
4236.9155	4236.9212	-0.0057	7	0	7	8	6	0	6	7
4498.3274	4498.3225	0.0049	7	2	6	6	6	2	5	5
4498.3274	4498.3279	-0.0005	7	2	6	7	6	2	5	6
4498.3274	4498.3180	0.0094	7	2	6	8	6	2	5	7
4573.2574	4573.2620	-0.0046	7	1	6	7	6	1	5	6
4573.3044	4573.2987	0.0057	7	1	6	8	6	1	5	7
4573.3065	4573.3124	-0.0059	7	1	6	6	6	1	5	5

4816.7445	4816.7441	0.0004	8	1	8	7	7	1	7	6
4816.7445	4816.7387	0.0058	8	1	8	8	7	1	7	7
4816.7445	4816.7401	0.0044	8	1	8	9	7	1	7	8
4818.8216	4818.8257	-0.0041	8	0	8	7	7	0	7	6
4818.8216	4818.8182	0.0034	8	0	8	8	7	0	7	7
4818.8216	4818.8215	0.0001	8	0	8	9	7	0	7	8
4853.6195	4853.6327	-0.0132	7	2	5	6	6	2	4	5
4853.6195	4853.6230	-0.0035	7	2	5	7	6	2	4	6
4853.6195	4853.6224	-0.0029	7	2	5	8	6	2	4	7
4860.3550	4860.3463	0.0087	7	3	4	6	6	3	3	5
4860.3550	4860.3531	0.0019	7	3	4	8	6	3	3	7
4860.4666	4860.4472	0.0194	7	3	4	7	6	3	3	6
5142.0962	5142.0945	0.0017	8	1	7	8	7	1	6	7
5142.1324	5142.1296	0.0028	8	1	7	7	7	1	6	6
5142.1324	5142.1208	0.0116	8	1	7	9	7	1	6	8
5304.3453	5304.3505	-0.0052	8	3	6	7	7	3	5	6
5304.3453	5304.3501	-0.0048	8	3	6	9	7	3	5	8
5400.8451	5400.8483	-0.0032	9	1	9	8	8	1	8	7
5400.8451	5400.8438	0.0013	9	1	9	9	8	1	8	8
5400.8451	5400.8453	-0.0002	9	1	9	10	8	1	8	9
5401.6630	5401.6676	-0.0046	9	0	9	8	8	0	8	7
5401.6630	5401.6624	0.0006	9	0	9	9	8	0	8	8
5401.6630	5401.6644	-0.0014	9	0	9	10	8	0	8	9
5484.8275	5484.8426	-0.0151	8	2	6	7	7	2	5	6
5484.8275	5484.8319	-0.0044	8	2	6	9	7	2	5	8
5691.3908	5691.3945	-0.0037	9	2	8	8	8	2	7	7
5691.3908	5691.3874	0.0034	9	2	8	9	8	2	7	8

5691.3908	5691.3906	0.0002	9	2	8	10	8	2	7	9
5713.5715	5713.5720	-0.0005	9	1	8	9	8	1	7	8
5713.5968	5713.5943	0.0025	9	1	8	8	8	1	7	7
5713.5968	5713.5885	0.0083	9	1	8	10	8	1	7	9
5931.6746	5931.6703	0.0043	9	3	7	8	8	3	6	7
5931.6746	5931.6819	-0.0073	9	3	7	9	8	3	6	8
5931.6746	5931.6681	0.0065	9	3	7	10	8	3	6	9
5984.6095	5984.6171	-0.0076	10	1	10	9	9	1	9	8
5984.6095	5984.6135	-0.0040	10	1	10	10	9	1	9	9
5984.6095	5984.6147	-0.0052	10	1	10	11	9	1	9	10
5984.9219	5984.9301	-0.0082	10	0	10	9	9	0	9	8
5984.9219	5984.9262	-0.0043	10	0	10	10	9	0	9	9
5984.9219	5984.9276	-0.0057	10	0	10	11	9	0	9	10
6050.7504	6050.7350	0.0154	9	4	6	8	8	4	5	7
6050.7504	6050.7383	0.0121	9	4	6	10	8	4	5	9
6050.7934	6050.7920	0.0014	9	4	6	9	8	4	5	8
6082.8949	6082.8948	0.0001	9	5	4	8	8	5	3	7
6082.8949	6082.9061	-0.0112	9	5	4	10	8	5	3	9
6209.3393	6209.3424	-0.0031	9	4	5	8	8	4	4	7
6209.3393	6209.3488	-0.0095	9	4	5	10	8	4	4	9
6209.4417	6209.4381	0.0036	9	4	5	9	8	4	4	8
6289.8942	6289.8882	0.0060	10	1	9	9	9	1	8	8
6289.8942	6289.8738	0.0204	10	1	9	10	9	1	8	9
6289.8942	6289.8842	0.0100	10	1	9	11	9	1	8	10
6292.3171	6292.3065	0.0106	9	3	6	8	8	3	5	7
6292.3171	6292.3226	-0.0055	9	3	6	9	8	3	5	8
6292.3171	6292.3027	0.0144	9	3	6	10	8	3	5	9

6545.3559	6545.3551	0.0008	10	3	8	9	9	3	7	8
6545.3559	6545.3557	0.0002	10	3	8	10	9	3	7	9
6545.3559	6545.3522	0.0037	10	3	8	11	9	3	7	10
6568.2505	6568.2520	-0.0015	11	1	11	10	10	1	10	9
6568.2505	6568.2490	0.0015	11	1	11	11	10	1	10	10
6568.2505	6568.2501	0.0004	11	1	11	12	10	1	10	11
6568.3602	6568.3689	-0.0087	11	0	11	10	10	0	10	9
6568.3602	6568.3658	-0.0056	11	0	11	11	10	0	10	10
6568.3602	6568.3669	-0.0067	11	0	11	12	10	0	10	11
6957.4683	6957.4735	-0.0052	10	3	7	9	9	3	6	8
6957.4683	6957.4626	0.0057	10	3	7	10	9	3	6	9
6957.4683	6957.4680	0.0003	10	3	7	11	9	3	6	10
6962.9045	6962.9093	-0.0048	10	4	6	9	9	4	5	8
6962.9045	6962.9119	-0.0074	10	4	6	11	9	4	5	10
7354.2652	7354.2616	0.0036	11	4	8	10	10	4	7	9
7354.2652	7354.2787	-0.0135	11	4	8	11	10	4	7	10
7354.2652	7354.2607	0.0045	11	4	8	12	10	4	7	11

Table S30: Observed and calculated rotational transitions (MHz) for 1-w(^{18}O)-E

Observed	Calculated	Obs-Calc	J'	K_a'	K_c'	F'	J"	K_a"	K_c"	F"
2566.2714	2566.2712	0.0002	4	0	4	4	3	0	3	3
2687.5709	2687.5877	-0.0168	4	2	2	5	3	2	1	4
2687.9023	2687.8951	0.0072	4	2	2	4	3	2	1	3
2704.8666	2704.8463	0.0203	4	1	4	4	3	0	3	3
2898.8818	2898.8739	0.0079	3	2	2	3	2	1	1	2
2899.8994	2899.9187	-0.0193	3	2	2	4	2	1	1	3
2900.4818	2900.4995	-0.0177	3	2	2	2	2	1	1	1

3033.8818	3033.8700	0.0118	5	0	5	4	4	1	4	3
3033.9821	3033.9685	0.0136	5	0	5	6	4	1	4	5
3034.1989	3034.1941	0.0048	5	0	5	5	4	1	4	4
3099.7338	3099.7492	-0.0154	3	2	1	2	2	1	2	1
3100.3686	3100.3763	-0.0077	3	2	1	4	2	1	2	3
3101.6418	3101.6518	-0.0100	3	2	1	3	2	1	2	2
3122.8159	3122.8186	-0.0027	5	1	5	6	4	1	4	5
3261.3287	3261.3133	0.0154	5	1	5	5	4	0	4	4
3261.8945	3261.8807	0.0138	5	1	5	6	4	0	4	5
3261.9814	3261.9705	0.0109	5	1	5	4	4	0	4	3
3271.9488	3271.9412	0.0076	5	2	4	6	4	2	3	5
3271.9488	3271.9480	0.0008	5	2	4	4	4	2	3	3
3316.5988	3316.5997	-0.0009	5	3	2	6	4	3	1	5
3316.7855	3316.7907	-0.0052	5	3	2	5	4	3	1	4
3385.8575	3385.8514	0.0061	5	2	3	6	4	2	2	5
3385.8575	3385.8408	0.0167	5	2	3	4	4	2	2	3
3386.0820	3386.0867	-0.0047	5	2	3	5	4	2	2	4
3390.6527	3390.6535	-0.0008	5	1	4	6	4	1	3	5
3390.7313	3390.7311	0.0002	5	1	4	4	4	1	3	3
3471.9606	3471.9473	0.0133	4	2	3	4	3	1	2	3
3661.8890	3661.8891	-0.0001	3	3	0	4	2	2	1	3
3661.8890	3661.8973	-0.0083	3	3	0	3	2	2	1	2
3735.2875	3735.2718	0.0157	6	1	6	6	5	1	5	5
3771.0784	3771.0662	0.0122	6	0	6	6	5	0	5	5
3771.2978	3771.2805	0.0173	6	0	6	7	5	0	5	6
3771.2978	3771.2902	0.0076	6	0	6	5	5	0	5	4
3823.8333	3823.8159	0.0174	6	1	6	6	5	0	5	5

3824.2345	3824.2447	-0.0102	6	1	6	5	5	0	5	4
3913.9516	3913.9535	-0.0019	6	2	5	6	5	2	4	5
3914.0215	3914.0219	-0.0004	6	2	5	5	5	2	4	4
3914.0215	3914.0122	0.0093	6	2	5	7	5	2	4	6
3967.7005	3967.7190	-0.0185	6	4	3	6	5	4	2	5
3968.9305	3968.9454	-0.0149	6	4	2	6	5	4	1	5
3969.3575	3969.3560	0.0015	6	3	4	7	5	3	3	6
3969.3575	3969.3500	0.0075	6	3	4	5	5	3	3	4
3969.4322	3969.4278	0.0044	6	3	4	6	5	3	3	5
3997.3883	3997.3897	-0.0014	6	3	3	6	5	3	2	5
4041.7996	4041.7908	0.0088	6	1	5	6	5	1	4	5
4041.9728	4041.9648	0.0080	6	1	5	7	5	1	4	6
4042.0366	4042.0254	0.0112	6	1	5	5	5	1	4	4
4086.1741	4086.1608	0.0133	6	2	4	7	5	2	3	6
4086.3192	4086.3108	0.0084	6	2	4	6	5	2	3	5
4175.5659	4175.5585	0.0074	7	1	6	8	6	2	5	7
4176.1096	4176.1122	-0.0026	7	1	6	7	6	2	5	6
4297.0882	4297.0814	0.0068	4	3	2	4	3	2	1	3
4297.4219	4297.4259	-0.0040	4	3	2	5	3	2	1	4
4297.5427	4297.5511	-0.0084	4	3	2	3	3	2	1	2
4314.4103	4314.3951	0.0152	7	0	7	8	6	1	6	7
4314.4103	4314.4089	0.0014	7	0	7	7	6	1	6	6
4334.7805	4334.7737	0.0068	4	3	1	5	3	2	2	4
4334.8699	4334.8675	0.0024	4	3	1	4	3	2	2	4
4344.2385	4344.2305	0.0080	7	1	7	7	6	1	6	6
4367.1771	4367.1586	0.0185	7	0	7	7	6	0	6	6
4367.3282	4367.3199	0.0083	7	0	7	8	6	0	6	7

4396.9991	4396.9802	0.0189	7	1	7	7	6	0	6	6
4397.2600	4397.2477	0.0123	7	1	7	6	6	0	6	5
4541.5517	4541.5450	0.0067	6	2	5	6	5	1	4	5
4542.5031	4542.4966	0.0065	6	2	5	7	5	1	4	6
4542.6878	4542.6882	-0.0004	6	2	5	5	5	1	4	4
4549.8374	4549.8342	0.0032	7	2	6	7	6	2	5	6
4549.9255	4549.9134	0.0121	7	2	6	8	6	2	5	7
4549.9255	4549.9241	0.0014	7	2	6	6	6	2	5	5
4631.3702	4631.3733	-0.0031	7	3	5	8	6	3	4	7
4634.7615	4634.7596	0.0019	7	4	4	8	6	4	3	7
4638.7425	4638.7326	0.0099	7	4	3	6	6	4	2	5
4638.7425	4638.7463	-0.0038	7	4	3	8	6	4	2	7
4675.8729	4675.8664	0.0065	7	1	6	7	6	1	5	6
4676.1080	4676.0903	0.0177	7	1	6	8	6	1	5	7
4780.6858	4780.6819	0.0039	7	2	5	8	6	2	4	7
4780.7411	4780.7461	-0.0050	7	2	5	7	6	2	4	6
4915.0352	4915.0305	0.0047	5	3	3	5	4	2	2	4
4915.5556	4915.5587	-0.0031	5	3	3	6	4	2	2	5
4915.6954	4915.7038	-0.0084	5	3	3	4	4	2	2	3
4917.8932	4917.8882	0.0050	8	1	7	8	7	2	6	7
4934.2588	4934.2553	0.0035	8	0	8	8	7	1	7	7
4934.2588	4934.2612	-0.0024	8	0	8	7	7	1	7	6
4950.4940	4950.4819	0.0121	8	1	8	8	7	1	7	7
4950.5631	4950.5548	0.0083	8	1	8	9	7	1	7	8
4964.0880	4964.0769	0.0111	8	0	8	8	7	0	7	7
4964.2048	4964.1956	0.0092	8	0	8	9	7	0	7	8
4980.3182	4980.3035	0.0147	8	1	8	8	7	0	7	7

4980.4863	4980.4699	0.0164	8	1	8	7	7	0	7	6
4980.4863	4980.4697	0.0166	8	1	8	9	7	0	7	8
5026.9632	5026.9828	-0.0196	5	3	2	6	4	2	3	5
5027.2306	5027.2365	-0.0059	5	3	2	5	4	2	3	4
5049.5987	5049.5884	0.0103	7	2	6	7	6	1	5	6
5050.4507	5050.4452	0.0055	7	2	6	8	6	1	5	7
5050.5859	5050.5869	-0.0010	7	2	6	6	6	1	5	5
5179.3191	5179.3225	-0.0034	8	2	7	9	7	2	6	8
5285.4627	5285.4691	-0.0064	8	6	3	9	7	6	2	8
5285.5683	5285.5870	-0.0187	8	6	3	8	7	6	2	7
5290.0815	5290.0778	0.0037	8	3	6	9	7	3	5	8
5291.6149	5291.6102	0.0047	8	1	7	8	7	1	6	7
5291.8843	5291.9046	-0.0203	8	1	7	7	7	1	6	6
5293.1429	5293.1481	-0.0052	8	5	4	9	7	5	3	8
5293.2376	5293.2460	-0.0084	8	5	4	8	7	5	3	7
5293.5684	5293.5783	-0.0099	8	5	3	9	7	5	2	8
5293.6712	5293.6778	-0.0066	8	5	3	8	7	5	2	7
5303.5465	5303.5481	-0.0016	8	4	5	9	7	4	4	8
5303.6254	5303.6250	0.0004	8	4	5	8	7	4	4	7
5314.1816	5314.1760	0.0056	8	4	4	9	7	4	3	8
5314.2961	5314.2906	0.0055	8	4	4	8	7	4	3	7
5394.3526	5394.3382	0.0144	8	3	5	9	7	3	4	8
5394.5421	5394.5361	0.0060	8	3	5	8	7	3	4	7
5463.9797	5463.9878	-0.0081	8	2	6	9	7	2	5	8
5498.3795	5498.3716	0.0079	6	3	4	6	5	2	3	5
5499.0612	5499.0633	-0.0021	6	3	4	7	5	2	3	6
5499.2088	5499.2129	-0.0041	6	3	4	5	5	2	3	4

5546.3142	5546.3044	0.0098	9	0	9	8	8	1	8	7
5546.3142	5546.3183	-0.0041	9	0	9	10	8	1	8	9
5552.9580	5552.9513	0.0067	8	2	7	8	7	1	6	7
5553.6597	5553.6774	-0.0177	8	2	7	9	7	1	6	8
5553.7706	5553.7781	-0.0075	8	2	7	7	7	1	6	6
5554.9250	5554.9241	0.0009	9	1	9	10	8	1	8	9
5562.6104	5562.5925	0.0179	9	0	9	10	8	0	8	9
5571.1042	5571.0858	0.0184	9	1	9	9	8	0	8	8
5571.2134	5571.1982	0.0152	9	1	9	10	8	0	8	9
5646.6036	5646.5876	0.0160	5	4	2	5	4	3	1	4
5650.6996	5650.7023	-0.0027	5	4	1	6	4	3	2	5
5650.6996	5650.7158	-0.0162	5	4	1	4	4	3	2	3
5752.1655	5752.1672	-0.0017	6	3	3	5	5	2	4	4
5752.2461	5752.2457	0.0004	6	3	3	7	5	2	4	6
5752.7158	5752.7112	0.0046	6	3	3	6	5	2	4	5
5802.2696	5802.2682	0.0014	9	2	8	9	8	2	7	8
5802.3741	5802.3690	0.0051	9	2	8	10	8	2	7	9
5892.3017	5892.3043	-0.0026	9	1	8	9	8	1	7	8
5892.5515	5892.5559	-0.0044	9	1	8	10	8	1	7	9
5943.8921	5943.8866	0.0055	9	3	7	10	8	3	6	9
5944.5822	5944.5923	-0.0101	9	7	2	8	8	7	1	7
5944.5822	5944.5917	-0.0095	9	7	3	8	8	7	2	7
5973.1269	5973.1164	0.0105	9	4	6	10	8	4	5	9
5973.1269	5973.1141	0.0128	9	4	6	8	8	4	5	7
5973.1732	5973.1751	-0.0019	9	4	6	9	8	4	5	8
5997.4253	5997.4313	-0.0060	9	4	5	10	8	4	4	9
5997.5497	5997.5557	-0.0060	9	4	5	9	8	4	4	8

6043.4717	6043.4693	0.0024	7	3	5	7	6	2	4	6
6044.2701	6044.2758	-0.0057	7	3	5	8	6	2	4	7
6044.4262	6044.4186	0.0076	7	3	5	6	6	2	4	5
6063.6100	6063.6092	0.0008	9	2	8	9	8	1	7	8
6064.1769	6064.1828	-0.0059	9	2	8	10	8	1	7	9
6064.2476	6064.2505	-0.0029	9	2	8	8	8	1	7	7
6106.6481	6106.6347	0.0134	9	3	6	8	8	3	5	7
6106.6481	6106.6421	0.0060	9	3	6	10	8	3	5	9
6106.8222	6106.8140	0.0082	9	3	6	9	8	3	5	8
6132.2569	6132.2562	0.0007	9	2	7	9	8	2	6	8
6132.3220	6132.3330	-0.0110	9	2	7	10	8	2	6	9
6153.6264	6153.6258	0.0006	10	0	10	9	9	1	9	8
6153.6264	6153.6368	-0.0104	10	0	10	11	9	1	9	10
6158.0857	6158.0801	0.0056	10	1	10	9	9	1	9	8
6158.0857	6158.0898	-0.0041	10	1	10	11	9	1	9	10
6166.6966	6166.6885	0.0081	10	1	10	9	9	0	9	8
6166.6966	6166.6956	0.0010	10	1	10	11	9	0	9	10
6297.5306	6297.5159	0.0147	6	4	3	6	5	3	2	5
6297.6869	6297.7068	-0.0199	6	4	3	5	5	3	2	4
6297.6869	6297.6708	0.0161	6	4	3	7	5	3	2	6
6313.2835	6313.2852	-0.0017	10	1	9	11	9	2	8	10
6313.2835	6313.2715	0.0120	10	1	9	9	9	2	8	8
6313.3831	6313.3836	-0.0005	10	1	9	10	9	2	8	9
6313.7883	6313.7848	0.0035	6	4	2	7	5	3	3	6
6313.7883	6313.7970	-0.0087	6	4	2	5	5	3	3	4
6419.5198	6419.5276	-0.0078	10	2	9	10	9	2	8	9
6419.6259	6419.6308	-0.0049	10	2	9	11	9	2	8	10

6484.6838	6484.6885	-0.0047	10	1	9	10	9	1	8	9
6527.8644	6527.8511	0.0133	7	3	4	6	6	2	5	5
6528.6600	6528.6769	-0.0169	7	3	4	7	6	2	5	6
6590.8246	6590.8325	-0.0079	10	2	9	10	9	1	8	9
6591.2454	6591.2577	-0.0123	10	2	9	11	9	1	8	10
6591.5125	6591.5266	-0.0141	10	3	8	11	9	3	7	10
6609.6706	6609.6913	-0.0207	10	7	3	9	9	7	2	8
6642.1687	6642.1747	-0.0060	10	4	7	9	9	4	6	8
6642.1687	6642.1823	-0.0136	10	4	7	11	9	4	6	10
6691.2113	6691.2122	-0.0009	10	4	6	11	9	4	5	10
6691.3550	6691.3550	0.0000	10	4	6	10	9	4	5	9
6758.2593	6758.2639	-0.0046	11	0	11	12	10	1	10	11
6758.2593	6758.2548	0.0045	11	0	11	10	10	1	10	9
6760.5082	6760.5204	-0.0122	11	1	11	10	10	1	10	9
6762.6982	6762.7090	-0.0108	11	0	11	10	10	0	10	9
6762.6982	6762.7170	-0.0188	11	0	11	12	10	0	10	11
6764.9644	6764.9821	-0.0177	11	1	11	12	10	0	10	11
6764.9644	6764.9747	-0.0103	11	1	11	10	10	0	10	9
6782.3995	6782.3934	0.0061	10	2	8	10	9	2	7	9
6782.5129	6782.5284	-0.0155	10	2	8	11	9	2	7	10
6818.4848	6818.4781	0.0067	10	3	7	11	9	3	6	10
6818.6069	6818.5997	0.0072	10	3	7	10	9	3	6	9
6934.9879	6934.9852	0.0027	7	4	4	7	6	3	3	6
6935.2406	6935.2263	0.0143	7	4	4	8	6	3	3	7
6969.0394	6969.0526	-0.0132	11	1	10	10	10	2	9	9
6969.0394	6969.0582	-0.0188	11	1	10	12	10	2	9	11
6983.1878	6983.1751	0.0127	7	4	3	8	6	3	4	7

6983.1878	6983.1858	0.0020	7	4	3	7	6	3	4	6
6983.1878	6983.1797	0.0081	7	4	3	6	6	3	4	5
7031.9081	7031.9247	-0.0166	11	2	10	11	10	2	9	10
7032.0160	7032.0261	-0.0101	11	2	10	12	10	2	9	11
7032.6991	7032.6957	0.0034	9	3	7	9	8	2	6	8
7033.5542	7033.5706	-0.0164	9	3	7	10	8	2	6	9
7033.6696	7033.6847	-0.0151	9	3	7	8	8	2	6	7
7138.0522	7138.0687	-0.0165	11	2	10	11	10	1	9	10
7138.4009	7138.3973	0.0036	11	2	10	10	10	1	9	9
7232.0883	7232.0982	-0.0099	11	3	9	11	10	3	8	10
7232.1515	7232.1596	-0.0081	11	3	9	12	10	3	8	11
7276.2308	7276.2520	-0.0212	11	7	5	12	10	7	4	11
7305.5028	7305.5025	0.0003	11	5	7	10	10	5	6	9
7305.5028	7305.5053	-0.0025	11	5	7	12	10	5	6	11
7314.5437	7314.5499	-0.0062	11	5	6	12	10	5	5	11
7314.6404	7314.6337	0.0067	11	5	6	11	10	5	5	10
7361.4176	7361.4133	0.0043	12	0	12	13	11	1	11	12
7361.4176	7361.4053	0.0123	12	0	12	11	11	1	11	10
7362.5570	7362.5496	0.0074	12	1	12	13	11	1	11	12
7362.5570	7362.5419	0.0151	12	1	12	11	11	1	11	10
7363.6763	7363.6710	0.0053	12	0	12	11	11	0	11	10
7363.6763	7363.6784	-0.0021	12	0	12	13	11	0	11	12
7364.8095	7364.8148	-0.0053	12	1	12	13	11	0	11	12
7364.8095	7364.8076	0.0019	12	1	12	11	11	0	11	10
7372.2465	7372.2530	-0.0065	8	3	5	7	7	2	6	6
7372.3787	7372.3809	-0.0022	8	3	5	9	7	2	6	8
7397.4153	7397.4162	-0.0009	11	4	7	12	10	4	6	11

7397.4153	7397.4069	0.0084	11	4	7	10	10	4	6	9
7397.5882	7397.5748	0.0134	11	4	7	11	10	4	6	10
7412.1075	7412.0997	0.0078	11	2	9	11	10	2	8	10
7412.2764	7412.2828	-0.0064	11	2	9	12	10	2	8	11
7548.6927	7548.6910	0.0017	8	4	5	8	7	3	4	7
7549.0706	7549.0518	0.0188	8	4	5	9	7	3	4	8
7604.6226	7604.6178	0.0048	12	1	11	12	11	2	10	11
7640.6152	7640.6321	-0.0169	12	2	11	13	11	2	10	12
7665.9768	7665.9778	-0.0010	8	4	4	9	7	3	5	8
7665.9768	7665.9717	0.0051	8	4	4	7	7	3	5	6
7666.0748	7666.0679	0.0069	8	4	4	8	7	3	5	7
7667.4475	7667.4675	-0.0200	12	1	11	12	11	1	10	11
7963.7635	7963.7656	-0.0021	13	0	13	12	12	1	12	11
7963.7635	7963.7727	-0.0092	13	0	13	14	12	1	12	13
7965.4578	7965.4726	-0.0148	13	1	13	14	12	0	12	13
7965.4578	7965.4659	-0.0081	13	1	13	12	12	0	12	11

Table S31: Observed and calculated rotational transitions (MHz) for 2-w(^{18}O,^{16}O)-E

Observed	Calculated	Obs-Calc	J'	K_a'	K_c'	F'	J"	K_a"	K_c"	F"
4461.9025	4461.8988	0.0037	7	2	5	6	6	2	4	5
4461.9025	4461.9098	-0.0073	7	2	5	8	6	2	4	7
4609.7466	4609.7665	-0.0199	8	0	8	8	7	1	7	7
4609.7466	4609.7463	0.0003	8	0	8	7	7	1	7	6
4609.7466	4609.7331	0.0135	8	0	8	9	7	1	7	8
4614.7329	4614.7164	0.0165	8	1	8	9	7	1	7	8
4614.7329	4614.7285	0.0044	8	1	8	7	7	1	7	6
4620.1921	4620.1754	0.0167	8	0	8	9	7	0	7	8

4620.1921	4620.1859	0.0062	8	0	8	7	7	0	7	6
4625.1759	4625.1587	0.0172	8	1	8	9	7	0	7	8
4625.1763	4625.1681	0.0082	8	1	8	7	7	0	7	6
4707.1778	4707.1688	0.0090	5	4	2	6	4	3	1	5
4707.8211	4707.8092	0.0119	5	4	1	4	4	3	1	3
4707.8211	4707.8130	0.0081	5	4	1	6	4	3	1	5
4713.0182	4713.0331	-0.0149	5	4	2	4	4	3	2	3
4713.0182	4713.0293	-0.0111	5	4	2	6	4	3	2	5
4713.6814	4713.6780	0.0034	5	4	1	4	4	3	2	3
4713.6814	4713.6735	0.0079	5	4	1	6	4	3	2	5
4934.0158	4934.0141	0.0017	8	3	6	7	7	3	5	6
4934.0158	4934.0192	-0.0034	8	3	6	9	7	3	5	8
5336.5584	5336.5760	-0.0176	6	4	2	7	5	3	3	6
5740.8779	5740.8816	-0.0037	10	0	10	9	9	1	9	8
5740.8779	5740.8729	0.0050	10	0	10	11	9	1	9	10
5743.2006	5743.1959	0.0047	10	0	10	9	9	0	9	8
5743.2006	5743.1876	0.0130	10	0	10	11	9	0	9	10
5743.2006	5743.2195	-0.0189	10	0	10	10	9	0	9	9
5744.2393	5744.2488	-0.0095	10	1	10	9	9	0	9	8
5744.2393	5744.2407	-0.0014	10	1	10	11	9	0	9	10
5931.3028	5931.3065	-0.0037	10	1	9	9	9	2	8	8
5931.3047	5931.3035	0.0012	10	1	9	11	9	2	8	10
5963.5889	5963.5728	0.0161	10	2	9	9	9	2	8	8
5963.5889	5963.5734	0.0155	10	2	9	11	9	2	8	10
5970.7147	5970.7216	-0.0069	7	4	3	8	6	3	4	7
6134.0168	6134.0204	-0.0036	10	3	8	11	9	3	7	10
6134.0168	6134.0165	0.0003	10	3	8	9	9	3	7	8

6258.8403	6258.8322	0.0081	6	6	1	7	5	5	0	6
6258.8403	6258.8195	0.0208	6	6	0	6	5	5	1	5
6258.8403	6258.8385	0.0018	6	6	0	7	5	5	1	6
6292.6872	6292.6798	0.0074	10	4	6	10	9	4	5	9
6304.4174	6304.4184	-0.0010	11	0	11	12	10	1	10	11
6304.4174	6304.4259	-0.0085	11	0	11	10	10	1	10	9
6304.8981	6304.8897	0.0084	11	1	11	12	10	1	10	11
6304.8981	6304.8971	0.0010	11	1	11	10	10	1	10	9
6304.8981	6304.9150	-0.0169	11	1	11	11	10	1	10	10
6305.4750	6305.4977	-0.0227	11	0	11	11	10	0	10	10
6305.4750	6305.4715	0.0035	11	0	11	12	10	0	10	11
6305.4750	6305.4788	-0.0038	11	0	11	10	10	0	10	9
6305.9485	6305.9500	-0.0015	11	1	11	10	10	0	10	9
6305.9485	6305.9428	0.0057	11	1	11	12	10	0	10	11
6305.9485	6305.9696	-0.0211	11	1	11	11	10	0	10	10
6405.9951	6405.9907	0.0044	7	5	3	6	6	4	2	5
6405.9951	6405.9904	0.0047	7	5	3	8	6	4	2	7
6409.5022	6409.5129	-0.0107	7	5	2	8	6	4	3	7
6409.5022	6409.5153	-0.0131	7	5	2	6	6	4	3	5
6514.7139	6514.7110	0.0029	11	1	10	10	10	2	9	9
6514.7139	6514.7094	0.0045	11	1	10	12	10	2	9	11
6595.2352	6595.2433	-0.0081	8	4	5	9	7	3	5	8
7013.9742	7013.9817	-0.0075	8	5	4	8	7	4	3	7
7013.9742	7013.9861	-0.0119	8	5	4	9	7	4	3	8
7013.9742	7013.9846	-0.0104	8	5	4	7	7	4	3	6
7015.5132	7015.5040	0.0092	8	5	3	8	7	4	3	7
7015.5132	7015.5114	0.0018	8	5	3	7	7	4	3	6

7015.5132	7015.5124	0.0008	8	5	3	9	7	4	3	8
7025.2408	7025.2518	-0.0110	8	5	4	9	7	4	4	8
7025.2408	7025.2176	0.0232	8	5	4	8	7	4	4	7
7025.2408	7025.2546	-0.0138	8	5	4	7	7	4	4	6
7026.7493	7026.7399	0.0094	8	5	3	8	7	4	4	7
7026.7805	7026.7814	-0.0009	8	5	3	7	7	4	4	6
7026.7805	7026.7781	0.0024	8	5	3	9	7	4	4	8
7340.7605	7340.7681	-0.0076	7	7	1	8	6	6	0	7
7340.7605	7340.7685	-0.0080	7	7	0	8	6	6	1	7
7340.7605	7340.7534	0.0071	7	7	1	7	6	6	0	6
7340.7605	7340.7538	0.0067	7	7	0	7	6	6	1	6
7648.0746	7648.0722	0.0024	9	5	4	10	8	4	5	9
7648.0746	7648.0765	-0.0019	9	5	4	8	8	4	5	7

Table S32: Observed and calculated rotational transitions (MHz) for 2-w(^{16}O,^{18}O)-E

Observed	Calculated	Obs-Calc	J'	K_a'	K_c'	F'	J"	K_a"	K_c"	F"
4320.0732	4320.0773	-0.0041	7	4	4	8	6	4	3	7
4320.0732	4320.0675	0.0057	7	4	4	6	6	4	3	5
4449.6489	4449.6401	0.0088	7	2	5	6	6	2	4	5
4449.6489	4449.6512	-0.0023	7	2	5	8	6	2	4	7
4450.1458	4450.1456	0.0002	13	10	3	13	13	9	4	13
4450.1458	4450.1465	-0.0007	13	10	4	13	13	9	5	13
4450.2096	4450.2116	-0.0020	13	10	4	12	13	9	5	12
4450.2096	4450.2108	-0.0012	13	10	3	12	13	9	4	12
4450.2096	4450.2070	0.0026	13	10	4	14	13	9	5	14
4588.8963	4588.8888	0.0075	8	0	8	9	7	1	7	8
4588.8963	4588.9019	-0.0056	8	0	8	7	7	1	7	6

4593.8936	4593.8990	-0.0054	8	1	8	7	7	1	7	6
4593.8936	4593.8869	0.0067	8	1	8	9	7	1	7	8
4599.3951	4599.3864	0.0087	8	0	8	7	7	0	7	6
4599.3951	4599.3759	0.0192	8	0	8	9	7	0	7	8
4604.3993	4604.3835	0.0158	8	1	8	7	7	0	7	6
4715.3694	4715.3655	0.0039	5	4	2	6	4	3	1	5
4715.3694	4715.3610	0.0084	5	4	2	4	4	3	1	3
4716.0058	4716.0236	-0.0178	5	4	1	6	4	3	1	5
4716.0058	4715.9937	0.0121	5	4	1	5	4	3	1	4
4716.0058	4716.0197	-0.0139	5	4	1	4	4	3	1	3
4721.3375	4721.3407	-0.0032	5	4	2	4	4	3	2	3
4721.3375	4721.3369	0.0006	5	4	2	6	4	3	2	5
4721.9874	4721.9950	-0.0076	5	4	1	6	4	3	2	5
4721.9874	4721.9995	-0.0121	5	4	1	4	4	3	2	3
4917.6993	4917.6827	0.0166	8	3	6	7	7	3	5	6
4917.6993	4917.6878	0.0115	8	3	6	9	7	3	5	8
4943.5140	4943.5223	-0.0083	8	4	5	7	7	4	4	6
4943.5140	4943.5275	-0.0135	8	4	5	9	7	4	4	8
5342.9902	5342.9975	-0.0073	6	4	2	7	5	3	3	6
5342.9902	5343.0081	-0.0179	6	4	2	5	5	3	3	4
5714.4800	5714.4812	-0.0012	10	0	10	11	9	1	9	10
5714.4800	5714.4900	-0.0100	10	0	10	9	9	1	9	8
5716.8020	5716.8082	-0.0062	10	0	10	9	9	0	9	8
5716.8020	5716.7999	0.0021	10	0	10	11	9	0	9	10
5717.9032	5717.8868	0.0164	10	1	10	10	9	0	9	9
5907.7624	5907.7562	0.0062	10	1	9	10	9	2	8	9
5909.8719	5909.8569	0.0150	7	4	3	6	6	3	3	5

5909.8719	5909.8700	0.0019	7	4	3	8	6	3	3	7
6186.0777	6186.0860	-0.0083	10	5	6	9	9	5	5	8
6186.0777	6186.0886	-0.0109	10	5	6	11	9	5	5	10
6275.2355	6275.2497	-0.0142	11	0	11	10	10	1	10	9
6275.2355	6275.2422	-0.0067	11	0	11	12	10	1	10	11
6275.6098	6275.6172	-0.0074	6	6	0	7	5	5	1	6
6275.6098	6275.5983	0.0115	6	6	0	6	5	5	1	5
6275.6098	6275.6107	-0.0009	6	6	1	7	5	5	0	6
6275.6098	6275.5917	0.0181	6	6	1	6	5	5	0	5
6275.7115	6275.7131	-0.0016	11	1	11	12	10	1	10	11
6275.7115	6275.7205	-0.0090	11	1	11	10	10	1	10	9
6276.2906	6276.2958	-0.0052	11	0	11	12	10	0	10	11
6276.2906	6276.3030	-0.0124	11	0	11	10	10	0	10	9
6276.7684	6276.7666	0.0018	11	1	11	12	10	0	10	11
6276.7684	6276.7738	-0.0054	11	1	11	10	10	0	10	9
6367.2837	6367.2808	0.0029	10	3	7	10	9	3	6	9
6367.2837	6367.2802	0.0035	10	3	7	9	9	3	6	8
6367.2837	6367.2835	0.0002	10	3	7	11	9	3	6	10
6415.4026	6415.3974	0.0052	7	5	3	8	6	4	2	7
6415.4026	6415.3977	0.0049	7	5	3	6	6	4	2	5
6418.9934	6418.9989	-0.0055	7	5	2	6	6	4	3	5
6418.9934	6418.9965	-0.0031	7	5	2	8	6	4	3	7
6488.4531	6488.4483	0.0048	11	1	10	10	10	2	9	9
6488.4531	6488.4467	0.0064	11	1	10	12	10	2	9	11
7021.1945	7021.1955	-0.0010	8	5	4	7	7	4	3	6
7021.1945	7021.1927	0.0018	8	5	4	8	7	4	3	7
7021.1945	7021.1970	-0.0025	8	5	4	9	7	4	3	8

7022.7674	7022.7590	0.0084	8	5	3	7	7	4	3	6
7022.7674	7022.7517	0.0157	8	5	3	8	7	4	3	7
7022.7674	7022.7600	0.0074	8	5	3	9	7	4	3	8
7032.6991	7032.7029	-0.0038	8	5	4	9	7	4	4	8
7032.6991	7032.7057	-0.0066	8	5	4	7	7	4	4	6
7034.2673	7034.2691	-0.0018	8	5	3	7	7	4	4	6
7034.2673	7034.2659	0.0014	8	5	3	9	7	4	4	8
7360.7717	7360.7819	-0.0102	7	7	1	8	6	6	0	7
7360.7717	7360.7676	0.0041	7	7	0	7	6	6	1	6
7360.7717	7360.7823	-0.0106	7	7	0	8	6	6	1	7
7360.7717	7360.7672	0.0045	7	7	1	7	6	6	0	6
7653.6675	7653.6694	-0.0019	9	5	4	8	8	4	5	7
7653.6675	7653.6652	0.0023	9	5	4	10	8	4	5	9

References

(S1) Greb, L.; Eichhöfer, A.; Lehn, J.-M. Synthetic Molecular Motors: Thermal N Inversion and Directional Photoinduced C=N Bond Rotation of Camphorquinone Imines. *Angewandte Chemie International Edition* **2015**, *54*, 14345–14348.

(S2) Schmitz, D.; Shubert, V. A.; Betz, T.; Schnell, M. Multi-resonance effects within a single chirp in broadband rotational spectroscopy: The rapid adiabatic passage regime for benzonitrile. *Journal of Molecular Spectroscopy* **2012**, *280*, 77–84.

(S3) Brown, G. G.; Dian, B. C.; Douglass, K. O.; Geyer, S. M.; Shipman, S. T.; Pate, B. H. A broadband Fourier transform microwave spectrometer based on chirped pulse excitation. *Review of Scientific Instruments* **2008**, *79*.

(S4) Kraitchman, J. Determination of Molecular Structure from Microwave Spectroscopic Data. *American Journal of Physics* **1953**, *21*, 17–24.

(S5) Kisiel, Z. *Spectroscopy from Space*; Springer, 2001; pp 91–106.

(S6) Pracht, P.; Bohle, F.; Grimme, S. Automated exploration of the low-energy chemical space with fast quantum chemical methods. *Physical Chemistry Chemical Physics* **2020**, *22*, 7169–7192.

(S7) Bannwarth, C.; Caldeweyher, E.; Ehlert, S.; Hansen, A.; Pracht, P.; Seibert, J.; Spicher, S.; Grimme, S. Extended tight-binding quantum chemistry methods. *Wiley Interdisciplinary Reviews: Computational Molecular Science* **2021**, *11*, e1493.

(S8) Grimme, S. Exploration of chemical compound, conformer, and reaction space with meta-dynamics simulations based on tight-binding quantum chemical calculations. *Journal of chemical theory and computation* **2019**, *15*, 2847–2862.

(S9) Bannwarth, C.; Ehlert, S.; Grimme, S. GFN2-xTB—An accurate and broadly parametrized self-consistent tight-binding quantum chemical method with multipole electrostatics and density-dependent dispersion contributions. *Journal of chemical theory and computation* **2019**, *15*, 1652–1671.

(S10) Neese, F. The ORCA program system. *WIREs Computational Molecular Science* **2012**, *2*, 73–78.

(S11) Neese, F. Software update: the ORCA program system, version 4.0. *WIREs Computational Molecular Science* **2018**, *8*, e1327.

(S12) Neese, F. Software update: The ORCA program system—Version 5.0. *WIREs Computational Molecular Science* **2022**, *12*, e1606.

(S13) Becke, A. D. Density-functional thermochemistry. III. The role of exact exchange. *The Journal of chemical physics* **1993**, *98*, 5648–5652.

(S14) Lee, C.; Yang, W.; Parr, R. G. Development of the Colle-Salvetti correlation-energy formula into a functional of the electron density. *Physical review B* **1988**, *37*, 785.

(S15) Vosko, S. H.; Wilk, L.; Nusair, M. Accurate spin-dependent electron liquid correlation energies for local spin density calculations: a critical analysis. *Canadian Journal of physics* **1980**, *58*, 1200–1211.

(S16) Stephens, P. J.; Devlin, F. J.; Chabalowski, C. F.; Frisch, M. J. Ab initio calculation of vibrational absorption and circular dichroism spectra using density functional force fields. *The Journal of physical chemistry* **1994**, *98*, 11623–11627.

(S17) Grimme, S.; Antony, J.; Ehrlich, S.; Krieg, H. A consistent and accurate ab initio parametrization of density functional dispersion correction (DFT-D) for the 94 elements H-Pu. *The Journal of chemical physics* **2010**, *132*.

(S18) Grimme, S.; Ehrlich, S.; Goerigk, L. Effect of the damping function in dispersion corrected density functional theory. *Journal of computational chemistry* **2011**, *32*, 1456–1465.

(S19) Weigend, F.; Ahlrichs, R. Balanced basis sets of split valence, triple zeta valence and quadruple zeta valence quality for H to Rn: Design and assessment of accuracy. *Physical Chemistry Chemical Physics* **2005**, *7*, 3297–3305.

(S20) Weigend, F. Accurate Coulomb-fitting basis sets for H to Rn. *Physical Chemistry Chemical Physics* **2006**, *8*, 1057–1065.
